# Novel Fluorophenylated Dihydroimidazotriazinones as Potential Anticancer Agents: Design, Synthesis, In Vitro, Ex Vivo and In Silico Characterisation

**DOI:** 10.3390/ijms27188388

**Published:** 2026-09-20

**Authors:** Małgorzata Sztanke, Jolanta Rzymowska, Małgorzata Janicka, Weronika Sofińska-Chmiel, Krzysztof Sztanke

**Affiliations:** 1 Department of Medical Chemistry, Medical University of Lublin, 4A Chodźki Street, 20-093 Lublin, Poland; 2 Department of Biology and Genetics, Medical University of Lublin, 4A Chodźki Street, 20-093 Lublin, Poland; 3 Department of Physical Chemistry, Institute of Chemical Science, Faculty of Chemistry, Maria Curie-Skłodowska University, Maria Curie-Skłodowska Sq. 3, 20-031 Lublin, Poland; 4 Analytical Laboratory, Institute of Chemical Science, Faculty of Chemistry, Maria Curie-Skłodowska University, Maria Curie-Skłodowska Sq. 3, 20-031 Lublin, Poland; 5 Laboratory of Bioorganic Compounds Synthesis and Analysis, Medical University of Lublin, 4A Chodźki Street, 20-093 Lublin, Poland

**Keywords:** fluorophenylated dihydroimidazotriazinones, fluorine–hydrogen isosterism, drug candidates, in vitro anticancer activity, apoptotic caspase levels, molecular target, adenosine A_2A_ and A_2B_ receptor antagonism, ex vivo haemolytic and antihaemolytic activity, ADME profiling, lipophilicity and in silico descriptors

## Abstract

This article describes the synthesis, structure confirmation, and evaluation of physico-chemical, pharmacological, and pharmacokinetic properties of new *para*-fluorophenylated dihydroimidazotriazinones. Based on the strategy of heteroannulation, and the concept of fluorine–hydrogen isosterism, an efficient and simple synthetic route was developed. The target heterobicycles (**10**–**18**) were obtained by the reaction of nucleophilic building blocks, i.e., 1-(R-phenyl)-2-hydrazinylideneimidazolidine hydroiodides (**1**–**9**), with an electrophilic two-carbon synthon, i.e., 2-(4-fluorophenyl)-2-oxoacetic acid. The structures of the new compounds were confirmed by spectroscopic data. The spectrum of anticancer activity, selectivity profile, and effect on caspase levels, as well as the haemolytic and antihaemolytic properties of the molecules were assessed in in vitro and ex vivo studies. Most compounds exhibited antiproliferative activity against human solid tumour and leukaemic cells (superior/comparable to anticancer drugs) with low toxicity to normal cells. Moreover, they revealed a broader spectrum of anticancer activity than previously obtained isosteres without fluorine substitution, suggesting that the isosteric replacement of hydrogen with fluorine was a fruitful modification. The most selective molecules were able to increase the levels of apoptotic caspases in lung, cervical, and breast cancer cells. Molecular docking results revealed that although all fluorophenylated fused triazinones exhibited affinity for the adenosine A_2A_ and A_2B_ receptors, ligands **13**, **16**, and **17** emerged as promising dual-target candidates due to their superior interaction profiles and stronger binding affinities for both receptor subtypes. All the compounds proved to be safe for red blood cells, as they did not induce any haemolytic effects. Additionally, most of them revealed protective effects on oxidatively stressed erythrocytes, and their antihaemolytic activity was better or comparable to that of antioxidant standards. In silico ADME (absorption, distribution, metabolism, excretion) profiling demonstrated favourable drug-like and pharmacokinetic properties of compounds. Lipophilicity of the molecules was measured by RP-HPLC on an ODS-2 column using an appropriately selected mobile phase. The retention factors, logs *k*, for most compounds showed a strong correlation with both in silico log *P* (logarithmic *n*-octanol/water partition coefficient) and log *P*_HSA_ (logarithmic human serum albumin/water partition coefficient) values. In summary, among the nine original fluorophenylated fused 1,2,4-triazinones studied, five molecules (**11**, **13**, **14**, **15**, and **17**) seem to be the most promising anticancer drug candidates suitable for further development.

## 1. Introduction

Isosteric replacement applies to both classical and non-classical isosteres. In the case of the former, it involves the exchange of atoms (univalent, divalent, trivalent) or atom groups (with similar effects) or aromatic/heterocyclic systems in the lead compound with atoms/groups of atoms/ring equivalents, respectively, that have a comparable steric arrangement and number of outer-shell electrons. In the case of the latter, however, this term refers to the replacement of groups in molecules with other groups that have physical (e.g., boiling point, density, thermal conductivity, viscosity) or chemical (e.g., reactivity) similarities, leading to essentially the same pharmacological activity profile [1,2]. Replacing hydrogen in biologically active molecules with isosteric fluorine (whose size is similar and virtually the same) is one of the strategies used in medicinal chemistry to design fluorine-substituted isosteres that can be further developed as potential drugs [3]. This tactic is intended, on the one hand, to improve the efficacy, selectivity, pharmacokinetic profile (in relation to ADME), or metabolic stability, and/or, on the other hand, to reduce the toxicity, undesirable effects, etc., of a lead structure requiring further optimisation. A large number of fluorine-containing pharmaceutics found application in human and veterinary medicine due to their therapeutic utility and favourable pharmacokinetic properties [3,4,5,6,7]. Fluorophenylated molecules are of great interest to medicinal chemists, biochemists and pharmacologists because replacing the enzymatically labile, electron-rich phenyl ring in a pharmacologically active molecule with the fluorine-substituted phenyl moiety (having an extremely stable C–F bond) often increases the biological effect, metabolic stability and bioavailability, and sometimes reduces the toxicity of isosteric structure [5]. The tactic of using fluorine as a hydrogen isostere proved to be successful, for example, in the development of gefitinib (an anticancer agent belonging to the class of kinase inhibitors that act on the EGF receptor domain) and flufenisal (an experimental non-steroidal anti-inflammatory drug). Having in mind that in the aromatic phenyl ring the *para* position is favoured for oxidation, in their molecules the fluorine atom was intentionally placed at this position to prevent metabolic hydroxylation. The positioning of this fluoro group was important since it acted as a metabolism blocker, preventing oxidation. Obstructive halogenation resulted in increased activity of gefitinib in vivo, prolonged the duration of action of flufenisal, and rendered both molecules more resistant to metabolic degradation [1,2]. Therefore, molecular variations based on isosteric replacement (including the replacement of phenyl with *para*-fluorophenyl) may prove to be a useful tactic in the design of innovative drug candidates.

So far, our research group has synthesised and studied numerous fused triazinones containing a common drug-like scaffold of 7,8-dihydroimidazo[2,1-*c*][1,2,4]triazin-4(6*H*)-one, some of which were selected as potential antimetabolite-type anticancer agents with an interesting spectrum of in vitro antiproliferative activity and reduced toxicity to normal cells, as well as being safe for early life stages of zebrafish (*Danio rerio*) [8,9,10,11,12]. However, none of these molecules possessed a fluorophenyl moiety attached to a heterobicyclic core. This reveals a research gap, as the synthesis, pharmacological properties, and pharmacokinetic profiles of dihydroimidazotriazinones bearing a *para*-fluorophenyl moiety remain unknown. Therefore, focusing on the structural design of novel fluorinated isosteres containing this privileged scaffold represents a highly promising strategy. Replacing hydrogen with fluorine in the phenyl moiety at *C*4′ is aimed at enhancing antiproliferative activity, improving bioavailability, broadening the spectrum of anticancer activity, or reducing toxicity compared to structurally related non-fluorinated analogues.

In search of innovative small molecules of potential anticancer agents, which may prove effective in treating human cancers, we decided to design—based on the concept of fluorine–hydrogen isosterism—novel heterobicycles with the *para*-fluorophenyl at *C*3 and nine different substituents at *N*8, i.e., 8-(R-phenyl)-3-(4-fluorophenyl)-7,8-dihydroimidazo[2,1-*c*][1,2,4]triazin-4(6*H*)-ones (**10**–**18**), the synthesis and characterisation of which have not been known so far. Their spectrum of anticancer activity will be assessed in comparison to that of previously synthesised isosteres lacking a fluorine substituent at the phenyl moiety [9]. Certain structural variations and modifications will be considered during the rational design of these target compounds.

As a result of isosteric replacement, we will be able to obtain, for the first time, highly conjugated fused asymmetrical-triazinones (*as*-triazinones) with a carbon–fluorine bond in the phenyl moiety. We suspect that the introduction of a fluorine atom (which mimics an atom of hydrogen from the steric point of view) in the *para* position of the phenyl ring will make this moiety less electron-rich, and will enhance both the metabolic stability (due to the presence of a particularly stable C–F bond) and the passage through biological barriers of the target compounds (by reducing their binding to plasma proteins and increasing their unbound, i.e., active, fraction), which will consequently enhance their bioavailability.

Drugs such as cytarabine and gemcitabine—nucleoside analogues of cytosine approved by the Food and Drug Administration for the treatment of cancer—are known to undergo rapid enzymatic deamination in vivo due to the attendance of a primary amino group, resulting in their unfavourable pharmacokinetics and rapid degradation to inactive metabolites [13].

**Figure 1:**
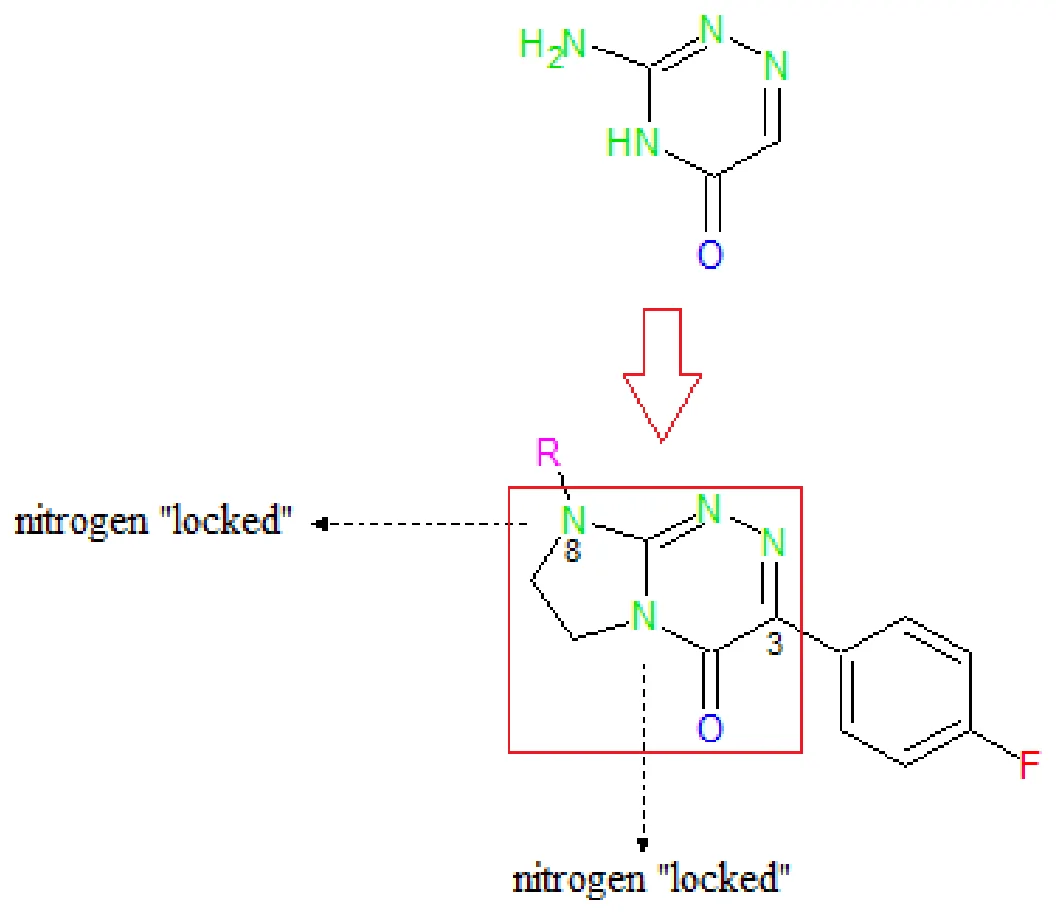
3-Amino-1,2,4-triazin-5(4*H*)-one—a tautomeric form of azaisocytosine present in the structure of difunctionalised 7,8-dihydroimidazo[2,1-*c*][1,2,4]triazin-4(6*H*)-ones.

Our newly designed compounds are related in structure to condensed nitrogen-enriched cytosine isosteres. To prevent metabolic inactivation of these fluorophenylated fused azaisocytosine-containing congeners by the enzyme cytidine deaminase and to prolong their duration time, some rational structural modifications will be considered. The azaisocytosine template will be incorporated into the bicyclic scaffold of target compounds (Figure 1). The nitrogen atom of exocyclic amino group as well as the *N*4 atom of endocyclic imino group of azaisocytosine (in its tautomeric form, i.e., 3-amino-1,2,4-triazin-5(4*H*)-one) will be rationally locked up into the imidazolidine ring. The closure of the nitrogen atom of the amino group into a ring will prevent in vivo deamination, thereby increasing the chemical stability of the molecule.

Therefore, the main aim of this study is to obtain—as a result of fruitful isosteric replacement and structural modifications—new more stable and bioavailable isosteres with enhanced potency and/or broader spectrum of antitumour activity, and/or reduced toxicity compared to previously disclosed congeners without fluorine substitution in the phenyl moiety at *C*3, exhibiting antiproliferative activity [9]. Confirmation of the more favourable pharmacological/pharmacokinetic profile of *para*-fluorinated fused *as*-triazinones compared to non-fluorinated analogues will be a significant contribution to the development of potential fluorinated drugs. To accomplish this, the following experimental protocol was applied: (1) development of an efficient and straightforward method for the synthesis of new condensed nitrogen-enriched cytosine isosteres—containing the *para*-fluorophenyl moiety at *C*3, and nine different substituents at *N*8—which, after optimisation, would be suitable for industrial-scale production, (2) determination of the structure and purity of the synthesised small molecules, (3) establishment of the spectrum of anticancer activity and toxicity to normal cells of the target compounds, (4) insight into the molecular mechanism of antiproliferative activity of the most selective molecules by investigating their ability to increase the levels of apoptotic caspases in tumour/normal cells, (5) evaluation of the haemolytic and antihaemolytic effects of the compounds, (6) in silico estimation of the risk of adverse side effects of molecules, (7) in silico ADME profiling of the compounds to assess their pharmacokinetic parameters and drug-likeness, (8) determination of lipophilicity indices of the molecules using a rapid and economical RP-HPLC method (with an ODS-2 column and an appropriately selected mobile phase), and (9) establishment of a quantitative retention–activity relationship (QRAR) between the logarithmic human serum albumin/water partition coefficients (logs *P*_HSA_)—modelled in silico—and the lipophilicity indices (logs *k*) of compounds—derived experimentally.

## 2. Results and Discussion

### 2.1. Design and Synthesis of Fluorophenylated Fused 1,2,4-Triazinones (***10**–**18***)

**Scheme 1:**
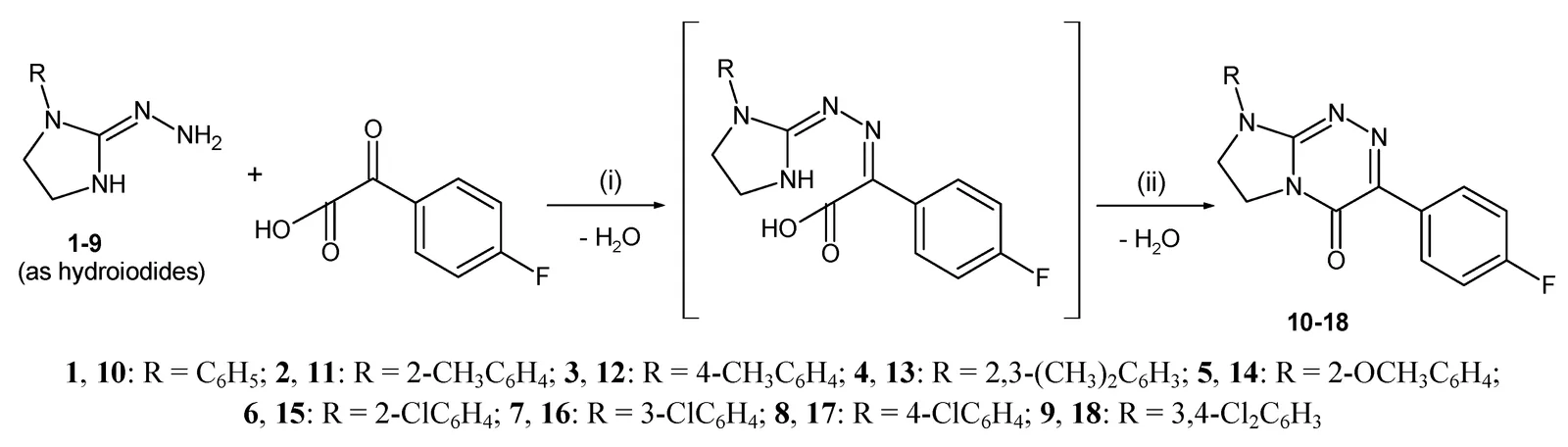
Synthesis of 7,8-dihydroimidazo[2,1-*c*][1,2,4]triazin-4(6*H*)-ones (**10**–**18**) with the *para*-fluorophenyl moiety at *C*3 and different substitution patterns at *N*8. Reagents and conditions: (i) Et_3_N, MeOH, r.t.; (ii) Et_3_N, MeOH/DMF mixture, reflux.

This is the first publication describing the design and synthesis of novel 3-(4-fluorophenyl)-8-(R-phenyl)-7,8-dihydroimidazo[2,1-*c*][1,2,4]triazin-4(6*H*)-ones (**10**–**18**). They were prepared by the reaction of 1-(R-phenyl)-2-hydrazinylideneimidazolidine hydroiodides (**1**–**9**)—building blocks synthesised in our laboratory according to the previous patent [14]—with 2-(4-fluorophenyl)-2-oxoacetic acid. All newly developed compounds mimic nitrogen bridgehead analogues of innovative bicyclic nucleobases. In their bicyclic core, a 3-amino-1,2,4-triazine-5(4*H*)-one template (a tautomeric form of azaisocytosine with an exocyclic amino group and an endocyclic imino group locked up to an imidazolidine skeleton) can be seen (Figure 1). The chemical stability of molecules **10**–**18** is enhanced by enclosing the nitrogen atom of the amino group in a ring, which prevents deamination in vivo. Simultaneously, the title molecules feature a 7,8-dihydroimidazo[2,1-*c*][1,2,4]triazin-4(6*H*)-one as the central template. This heterobicyclic scaffold is substituted by the *para*-fluorophenyl moiety at *C*3 as well as a phenyl or R-substituted phenyl at *N*8. Having in mind that in the phenyl ring, the *para* position is favoured for metabolic oxidation [2], to protect this aromatic ring from metabolism we applied an appropriately substituted 2-ketoacid as reagent that allowed the introduction of a 4-fluorophenyl moiety into the heterocyclic scaffold at *C*3. Taking into account that the presence of different substitution patterns at *N*8 can be beneficial from the perspective of finding a relationship between the compound structure and its activity/selectivity, we chose—as a basis for the synthesis of the title molecules—nine variously substituted imidazolidin-2-one hydrazone hydroiodides (**1**–**9**), bearing phenyl or phenyl substituted with electron-donating (i.e., 2-methylphenyl, 4-methylphenyl, 2,3-dimethylphenyl, 2-methoxyphenyl) or electron-withdrawing (i.e., 2-chlorophenyl, 3-chlorophenyl, 4-chlorophenyl, 3,4-dichlorophenyl) groups. These materials—due to the enhanced nucleophilicity of the terminal nitrogen atom in the =N–NH_2_ functional group—exhibit high reactivity towards electrophilic reagents. Therefore, our research team has frequently applied them as highly useful building blocks in many syntheses of heterocyclic small molecules of pharmacological importance [8,9]. The original synthetic route—leading to a novel class of fluorinated molecules, i.e., 3-(4-fluorophenyl)-8-(R-phenyl)-7,8-dihydroimidazo[2,1-*c*][1,2,4]triazin-4(6*H*)-ones (**10**–**18**)—which was developed in our research laboratory, and then patented [15,16], is outlined in Scheme 1.

When designing the target compounds, some rational strategies and molecular replacements were considered to obtain new fluorophenylated molecules with antiproliferative activity, which should be resistant to metabolic deamination (due to the closure of the free amino group of azaisocytosine in a heterocyclic ring) and in vivo degradation via *para*-hydroxylation (due to the attendance of a stable carbon–fluorine bond in the phenyl moiety at *C*4′). To achieve this, the concept of fluorine–hydrogen isosterism was taken into account, and an experimental protocol based on the heteroannulation strategy—optimised for all necessary operations—was developed. Attempts to construct the target compounds (**10**–**18**) using highly reactive nucleophiles, i.e., 1-(R-phenyl)-2-hydrazinylideneimidazolidines (**1**–**9**), were successful when an excellent annulation reagent, i.e., 2-(4-fluorophenyl)-2-oxoacetic acid, was used. The sp^2^-hybridised electrophilic centre of this reagent was prone to electrophilic attack primarily due to the presence of the *C*2 carbonyl, which was activated by the adjacent electron-withdrawing carboxylic acid group. In this case, the desired 1,2,4-triazinone template could be annulated onto the already existing hydrazinylideneimidazolidine fragment (N–C–N–N) using the above-mentioned *para*-fluorophenyl-substituted α-oxoacid as the two-carbon synthon (C–C). In this way, the heterobicyclic scaffold with a bridgehead nitrogen atom was built according to the [4 + 2] pattern for annulation. Following this method, nine new fluorophenylated fused *as*-triazinones (**10**–**18**)—including the unsubstituted parent compound (**10**) as well as its derivatives with electron-donating (**11**–**14**) and electron-withdrawing (**15**–**18**) substituents at the phenyl moiety—could be readily prepared from appropriately substituted hydrazinylideneimidazolidines in good overall yields under relatively mild reaction conditions. This suggests that the general synthesis method is effective and relatively straightforward, and therefore has the potential for future production-scale application.

The synthesis of the new products (**10**–**18**) was shown as a two-stage route, with ketimine as an intermediate, proceeding via condensation and heterocyclisation processes. In the first step, when both starting materials were stirred at room temperature using methanol (containing triethylamine) as solvent, loss of a water molecule must have led to the formation of 2-(4-fluorophenyl)-{2-[1-(R-phenyl)imidazolidin-2-ylidene]hydrazinylidene}acetic acid with a characteristic C=N bond (shown in square brackets in Scheme 1) as the condensation product. It was hypothesised that the condensation step was preceded by the formation of a hemiaminal (nucleophilic addition intermediate) that dehydrated to form a ketimine. Since efficient imine synthesis—accomplished by dehydration—often requires a catalytic amount of base [17], this process, carried out at room temperature in a methanolic medium, most likely had to be facilitated by the presence of a Lewis base, i.e., Et_3_N. In the second step, upon heating in organic solvents under reflux conditions, the non-isolated ketimine-type intermediates could be converted in situ to highly conjugated, and therefore stable, fluorophenylated fused 1,2,4-triazinones (**10**–**18**). Thus, the core bicyclic scaffold (with a bridgehead nitrogen atom) of the target products was assembled via intramolecular heterocyclisation, which ended with the junction of *C*4-*N*5 atoms and the closure of the *as*-triazinone ring. While studying this cyclocondensation step, it was found that this process proceeds most readily on boiling in a mixture of polar protic/polar aprotic (MeOH/DMF) solvents. In this case, there was no doubt that under established reaction conditions a water molecule was released from each intermediate ketimine to form the final product. This was confirmed by successful heterocyclisation to the fluorophenylated fused 1,2,4-triazinone **10** under azeotropic reflux with water removal.

During the synthesis of compounds **10**–**18**, hydrogen iodide (from each starting hydrazinylidenemidazolidine) reacted with triethylamine to form triethylammonium iodide (Et_3_NH^+^I^−^) as an alkylation side product. The presence of Et_3_NH^+^I^−^ was confirmed by melting point analysis of this unwanted side-product isolated from the reaction mother liquors (decomposition at m.p. 173 °C [18]). Therefore, it was especially important to obtain uncontaminated final products. For this purpose, differences in the solubility of all end products and the side-product were investigated. Due to the significantly lower solubility (during filtration, washing and recrystallisation processes) of the main products compared to that of the side-product, target molecules **10**–**18** of high purity were isolated. The purity of each new compound (>99.7%) was confirmed by the presence of a single, symmetrical peak in the HPLC chromatogram (Figures S1–S9 in the Supplementary Material). In addition, an undesirable side-product could be successfully removed, avoiding the chromatographic separation process, and cutting down the number of necessary operations throughout the synthesis of the desired products.

The presented efficient and straightforward general synthesis protocol—with a limited number of necessary operations and a simple isolation procedure—allows accessing many unknown disubstituted (with the *para*-fluorophenyl at *C*3 and various substituents at *N*8) derivatives of 7,8-dihydroimidazo[2,1-c][1,2,4]triazin-4(6*H*)-one, which are essential for further studies.

All obtained compounds are small molecules with molecular weight below 380 Da. They proved to be stable in organic solvents under air atmosphere. This could be explained due to a high degree of conjugation of double bonds within the *as*-triazinone template and the presence of a highly stable C–F bond in the fluorophenyl moiety.

The structures of compounds **10**–**18** were resolved based on ^1^H NMR, ^13^C NMR and ATR-IR spectra, which are consistent with their assigned structural formulae. Full spectral data are presented in the experimental section. Copies of the spectra of all compounds are provided in the Supplementary Material (Figures S10–S36).

It is worth noting that the attendance—in the dihydroimidazotriazinone skeleton—of two endocyclic methylene bridges (with secondary carbon atoms) and three quaternary carbon atoms incorporated is a common feature of all new molecules. Therefore, the ^1^H NMR spectra of compounds’ solutions in DMSO-*d*_6_ showed signals that can be attributed to the presence of ethylene protons (–C(6)H_2_–C(7)H_2_–). Furthermore, in the ^13^C NMR spectra of all molecules, the signals of quaternary carbon atoms, such as C4 (endocyclic triazinone carbonyl), C8a (C=N bond at the junction of rings building the heterobicyclic template), and C3, appeared at approximately 152.7, 152.3, and 146.9 ppm, respectively. The protons of the two methylene bridges exhibited different chemical shifts and a characteristic splitting pattern in the ^1^H NMR spectra of the investigated compounds. For molecules **10**, **12**, **16**, **17**, and **18**, these aliphatic protons showed nearly identical chemical shifts, appearing as a singlet (4H) at ca. δ 4.22 ppm. In turn, for compounds **11**, **13**, **14**, and **15**—which contain a 2-methyl, 2,3-dimethyl, 2-methoxy, and 2-chloro group in the phenyl moiety, respectively—the corresponding signals manifested as two apparent triplets (2H and 2H) in the aliphatic region (ca. δ 4.30 and 4.06 ppm) with *J* ~ 8.8 Hz. The discrepancies in the chemical shifts and multiplicity of the proton signals for the C(6)H_2_ and C(7)H_2_ groups (a singlet vs. two well-resolved pseudo-triplets (Δδ = 0.24 ppm)) can be rationalised by the absence or presence of steric hindrance, which likely either allows or restricts free rotation around the single bond (*C*_aryl_-N(8)) connecting the phenyl ring to the dihydroimidazo[2,1-*c*][1,2,4]triazine core [19]. The aryl moiety lacking an *ortho* substituent in compounds **10**, **12**, **16**, **17**, and **18** can rotate freely and rapidly around this bond, presumably due to the absence of steric clash. This rapid rotation establishes a time-averaged plane of symmetry, causing the C(6)H_2_ and C(7)H_2_ protons to resonate at the same chemical shift and appear as a sharp singlet [20,21]. In contrast, introducing a substituent at the *ortho* position of the phenyl ring in compounds **11**, **13**, **14**, and **15** likely induces significant steric hindrance (particularly with the adjacent C(7)H_2_ group and the carbonyl oxygen C(4)=O)) [22]. When this rotation is blocked by an *ortho* substituent, the molecules adopt an asymmetric conformation on the NMR timescale. In this conformationally restricted state, the magnetic anisotropy (ring-current effect) of the phenyl ring affects the C(6)H_2_ and C(7)H_2_ protons unequally [23]. Consequently, the protons of these two structurally adjacent methylene groups create a chemically non-equivalent but magnetically coupled spin system, splitting the resonances into a characteristic pair of apparent triplets [20]. The non-equivalence of the two methylene bridges (C(6)H_2_ and C(7)H_2_) was inferred from their ^13^C NMR spectra, in which higher chemical shift values of the C7 atom (at δ between 45.6 and 48.6 ppm) and lower shift values of the C6 atom (at δ between 40.9 and 42.2 ppm) were clearly observed. This was to be expected based on our previous crystallographic studies carried out on the structurally related disubstituted fused *as*-triazinone. At that time, it was found that in 8-(4-methylphenyl)-3-(propan-2-yl)-7,8-dihydroimidazo[2,1-*c*][1,2,4]triazin-4(6*H*)-one the five-membered imidazolidine ring is able to adopt an envelope conformation with one of the CH_2_ groups (i.e., C(7)H_2_) as a flap [24].

Another common feature of the studied molecules is the attendance of a *para*-fluorophenyl moiety at *C*3. Since the nuclear spin of fluorine is ½ and fluorine is capable of coupling with nearby carbon atoms, key ^13^C–^19^F couplings were observed in the ^13^C NMR spectra: one-bond coupling, two-bond coupling, and three-bond coupling. The ipso-carbon (C4′–F) signals were detected in the most downfield shifted region at δ ca. 164.1 and 162.2 ppm as doublets with a coupling constant ^1^*J_C–F_* = 246.2–247.4 Hz. The C3′ and C5′ carbon atoms—located *ortho* to the fluorine atom—appeared as doublets at δ ca. 115.5 and 115.4 ppm with a coupling constant of ^2^*J_C–F_* = 21.2–21.6 Hz. In turn, the C2′ and C6′ carbon atoms—placed *meta* to the fluorine atom—were recorded as doublets at δ ca. 130.6 and 130.5 with a coupling constant of ^3^*J_C–F_* = 8.2–8.3 Hz. It can be seen that the magnitude of the splitting decreases as the number of bonds between the carbon and fluorine increases.

In turn, in the ATR-IR spectrum of each compound, the most characteristic were two strong absorption bands: the first in the range of 1664–1680 cm^−1^ (which could be assigned to the attendance of the endocyclic carbonyl function (triazine-C=O)) and the second in the range of 1542–1564 cm^−1^ (which clearly indicated the presence of azomethine (C=N) groups). Normally one would expect the carbonyl group to absorb at a somewhat higher frequency, but most likely the coupling with the C=N bond lowered this absorption band. In the present study, the difference between the frequencies of the carbonyl and azomethine group (at the junction of both heterocyclic rings) was found to be in the range of 113 to 138 cm^−1^. Le Count and Taylor reported a similar relationship in the infrared spectrum of a structurally related congener with a completely conjugated triazinone system (i.e., 3-methyl-8-phenyl-7,8-dihydroimidazo[2,1-*c*][1,2,4]triazin-4(6*H*)-one) [25]. Another characteristic strong absorption band in the range from 1309 to 1323 cm^−1^ could be attributed to carbon–fluorine (C–F) stretching vibrations, resulting from a common structural feature of fluorophenylated molecules **10**–**18**.

### 2.2. Anticancer Activity of Fluorophenylated Fused 1,2,4-Triazinones (***10**–**18***)

The anticancer activity of fluorophenylated fused *as*-triazinones (**10**–**18**) was assessed in six cell lines (A549—human Caucasian lung carcinoma cell line; HeLa—human Negroid cervix epithelioid carcinoma cell line; T47D—human breast carcinoma cell line, MM.1R—human multiple myeloma cell line; Jurkat E6-1—human acute T-lymphocytic leukaemia cell line; and HL-60—human acute promyelocytic leukaemia cell line). In turn, their cytotoxicity to non-tumour cells was evaluated in the GMK (African green monkey kidney) cell line. Anticancer agents such as pemetrexed and gemcitabine were used for comparative purposes. Results of preliminary screening of cytotoxicity (expressed as the percentage of growth inhibition of GMK, A549, HeLa and T47D cells and as the percentage of dead MM.1R, Jurkat E6-1 and HL-60 cells) of molecules **10**–**18** and standard drugs are presented in Tables S1 and S2 in the Supplementary Material. However, half-maximal inhibitory concentration (IC_50_) values (calculated from dose/response curves) and selectivity indices (SIs) (calculated from the ratio of the IC_50_ value of the compound in the non-cancer cell line to the IC_50_ value of the compound in the cancer cell line) are given in Table 1.

As seen in Table 1, the studied compounds showed the highest antiproliferative activity against acute promyelocytic leukaemia cells (HL-60), as well as cervical (HeLa), lung (A549), and breast (T47D) cancer cells. The lowest IC_50_ values were observed in these cell lines. However, lower anticancer activity of the molecules was demonstrated in a human multiple myeloma cell line (MM.1R), and the lowest in a human acute T-lymphocytic leukaemia cell line (Jurkat E6-1).

The half-maximal inhibitory concentration values in tumour epithelial (A549, HeLa, and T47D) cell lines (after three incubation periods) of all the molecules studied were shown to be lower than those of clinically used anticancer drug pemetrexed. Moreover, the activity of most fluorinated compounds was higher than that of another anticancer drug, gemcitabine. Thus, lower IC_50_ values than for gemcitabine were observed in the A549 cell line for heterocycles **12** and **18** (after all incubation periods) and for **16** (after 24 h of incubation), in the HeLa cell line for compounds **10**, **11**, **12**, **16**, **17** and **18** (after 24–72-h incubation periods), in the T47D cell line for molecules **10** and **12** (after all incubation periods), for **16**, **17** and **18** (after 48 and 72 h of incubation) and for **11** (after a 72-h incubation period), while they were observed in the HL-60 cell line for almost all compounds (except for **10** and **16** after 24 and 48 h of incubation).

In the normal GMK cell line, the IC_50_ values for dihydroimidazotriazinones **11**, **13**, **14**, **15** and **17** were comparable to those for pemetrexed. Simultaneously, they were higher (after a 72-h incubation period) or similar (after 24 and 48 h of incubation) to the ICs_50_ for gemcitabine. This indicates that compounds **11**, **13**, **14**, **15** and **17** are the least toxic to normal cells.

The majority of fluorinated fused *as*-triazinones displayed favourable selectivity indices (SIs) (Table 1). Most compounds showed higher selectivity indices than standard anticancer drugs such as pemetrexed and gemcitabine. For A549 cells, molecules **13** (after a 72-h incubation period), **14**, **15**, **17** (after 48 and 72 h of incubation), **16** (after 24 and 48 h of incubation) and **18** (after all incubation periods) were less toxic than pemetrexed, while **16** and **18** (after a 24-h incubation period) were less toxic than gemcitabine. In the HeLa cell line, heterocycles **10**, **11**, **14**, **16**, **17**, **18** (after all incubation periods), **12**, **13** (after 72 h of incubation) and **15** (after 48 and 72 h of incubation) showed higher selectivity indices than those for pemetrexed, while molecules **11**, **17** (after all incubation periods), **10**, **16** (after 24 and 48 h of incubation), **14** and **18** (after a 24-h incubation period) showed higher selectivity than those for gemcitabine. In turn, for T47D cells, compounds **10** (after all incubation periods), **11**, **12** (after a 72-h incubation period), **16** (after 48 h of incubation), **17** and **18** (after 48- and 72-h incubation periods) were found to be safer than both pemetrexed and gemcitabine.

It is worth noting that some fluorinated dihydroimidazotriazinones were several fold (up to almost 8-fold) less toxic to normal cells than to cancer cells. The highest SIs were observed for molecules **11** and **17**, which were 7.9- and >7.5-fold, respectively, less toxic to GMK cells than to HeLa (in the case of **11** and **17**) and T47D (in the case of **17**) cells after 72 h of incubation. Molecule **17** was also more than 6.5-fold more toxic to Hela cells after a 48-h incubation period. Therefore, these compounds will be more effective in destroying cancer cells than normal cells. Moreover, they will cause less damage to proliferating epithelial cells, such as those of the gastrointestinal epithelium, which is highly desirable and important for further research into the development of these promising drug candidates.

Interestingly, the developed fluorinated fused *as*-triazinones showed a broader spectrum of anticancer activity than the compounds mentioned earlier, i.e., their isosteres without fluorine substitution at the phenyl moiety, which were only able to cause a decrease in the viability of peripheral blood myeloma (RPMI 8226) cells or inhibit the motility of cervical cancer (HeLa B) cells [9]. Noteworthy is that isosteric replacement of a hydrogen atom with a fluorine atom in the phenyl ring at *C*4′ in all synthesised molecules proved to be a fruitful modification. Novel fluorinated dihydroimidazotriazinones revealed an enhanced activity spectrum against human tumour cells of the lung, cervix and breast, human multiple myeloma cells as well as leukaemic cells: human acute T-lymphocytic leukaemia cells and human acute promyelocytic leukaemia cells.

### 2.3. Structure–Activity Relationships

The anticancer activity of fluorinated fused *as*-triazinones (**10**–**18**) depended on the incubation time and the substitution pattern in the phenyl ring at *N*8 (Table 1).

Among the molecules in this class, dihydroimidazotriazinones **11**, **13**, **14**, **15** and **17**, containing the *ortho*-methyl, 2,3-dimethyl, *ortho*-methoxy, *ortho*-chloro or *para*-chloro substituent at the phenyl moiety, respectively, were found to be the least toxic to non-tumour cells. These represent the best substitution patterns in terms of toxicity to normal cells.

It was proven that the parent structure (**10**) reveals the strongest antiproliferative activity in HeLa and T47D cells (after all incubation periods) among the compounds tested. This molecule was also effective against MM.1R cells (after 72 h of incubation). Introducing a methyl group *ortho* to the phenyl moiety reduced the antiproliferative activity of molecule **11** against T47D and HeLa cells (after 24 and 48 h of incubation), but maintained the activity against MM.1R cells. However, in terms of toxicity to normal cells, this modification proved to be fully beneficial, because derivative **11** is one of the least toxic compounds for GMK cells. Placing an electron-donating methyl group *para* to the phenyl ring significantly enhanced the antiproliferative activity of molecule **12** against A549 and HL-60 cells (after all incubation periods), retained the activity against HeLa, T47D and MM.1R cells, but caused an unwanted increase in cytotoxicity towards normal GMK cells. Considering the toxicity of both positional isomers with methyl group (**11** and **12**) to normal cells, it was noted that only the *ortho*-methyl substitution in **11** is advantageous due to the significant reduction in this cytotoxicity compared to the parent structure. Introducing an additional methyl group at position 3 to the 2-methyl derivative (**11**) resulted in an enhanced antiproliferative potential of 2,3-dimethyl-substituted compound **13** towards A549 (after 72 h of incubation) and HL-60 (after all incubation periods) cells, but a decreased activity in HeLa and T47D cells. At the same time, this molecule proved to be as non-toxic to normal cells as compound **11**. Replacing an *ortho-*methyl group (**11**) at the phenyl moiety with an *ortho-*methoxy group (**14**) clearly enhanced the antiproliferative potential against HL-60, MM.1R (after all incubation periods) and A549 (after 72 h of incubation) cells, but resulted in decreased cytotoxicity against HeLa and T47D cells (after 72 h of incubation). Both substitution patterns (with the *ortho*-methyl and *ortho*-methoxy group) were favourable in terms of selectivity, as they resulted in more selective molecules (**11** and **14**)—showing a decrease in cytotoxicity towards normal cells (after all incubation periods)—compared to **10**. The isosteric replacement of a methyl group placed at an *ortho* position of the phenyl moiety in **11** with a chloro substituent proved effective in the HL-60 cell line, resulting in a molecule (**15**) with the highest activity against these cells among compounds of this class, while being nontoxic to normal cells. On the other hand, this modification was responsible for the reduced cytotoxicity in HeLa, T47D and MM.1R cells. The replacement of a methyl group located at a *para* position of the phenyl ring in **12** with a chloro substituent was beneficial on the one hand, as it led to a significant reduction in the cytotoxicity of analogue **17** towards normal cells and an increase in antiproliferative activity against MM.1R and Jurkat E6-1 cells (especially after 72 h of incubation), but on the other hand, it was ineffective, as it resulted in a reduction in the antiproliferative potential against A549 (after all incubation periods) and T47D (after 24 and 48 h of incubation) cells. Moving a chloro substituent from the *para* to the *ortho* or *meta* position of the phenyl moiety allowed the production of structures **15** and **16**. The *ortho*-chloro-substituted compound (**15**) demonstrated no toxicity to normal GMK cells, significantly increased antiproliferative activity in HL-60 cells, but decreased activity in other cell lines compared to molecule **17**. In turn, the *meta*-chloro isomer (**16**) showed increased or similar antiproliferative effect to **17** only in A549 cells. Introducing an additional chloro group to the *meta*-chloro (**16**) or *para*-chloro (**17**) derivative enhanced the antiproliferative effect of 3,4-dichloro-substituted counterpart (**18**), especially in A549, HL-60 (after all incubation periods), HeLa (after 24 and 48 h of incubation) and T47D (after 48 and 72 h of incubation) cells. Compound **18** showed the strongest cytotoxicity against A549 cells among all the studied molecules.

In summary, substituents such as *ortho*-methyl, *ortho*-methoxy, *ortho*-chloro, 2,3-dimethyl and *para*-chloro in the phenyl ring were found to be the best substitution patterns, as their presence in the molecules resulted in increased selectivity of these five fluorinated fused 1,2,4-triazinones. Thus, special attention should be focused on small molecules **11**, **13**, **14**, **15** and **17** with an established spectrum of antiproliferative activity, which reveal markedly lower cytotoxicity towards non-tumour cells. Accordingly, these compounds—as possible innovative nucleobases—will be utilised in the rational design of less toxic anticancer agents.

### 2.4. The Effect of the Selected Fluorophenylated Fused 1,2,4-Triazinones on Proapoptotic Caspases in Normal and Tumour Cells

Mammalian caspases 3, 7 and 9 are apoptotic caspases, belonging to the cysteine-aspartic proteases. These proteolytic enzymes—capable of degrading proteins in tumour cells—are known to play a crucial role in programmed cell death, both in its initiation and execution. Caspase-9 is an initiator caspase that is activated via the extrinsic or intrinsic pathway of apoptosis, whereas caspases 3 and 7 are effector caspases that are synthesised as procaspases—inactive zymogens, and mediate the cleavage of cellular components during apoptosis. It was proved experimentally that the initiator caspase-9 mediates the activation of effector caspases 3 and 7 [26,27,28]. Without caspases, cells could be more susceptible to uncontrolled growth, leading to the development of cancer. Apoptotic caspases are regarded by medicinal chemists as potentially useful drug targets [2]. Since apoptosis is mediated by caspases, it is important to search for novel more effective anticancer agents that should inhibit cell proliferation in tumour cells, and also activate apoptosis-inducing caspases to increase their anticancer potential [27,28,29,30].

The most selective fluorophenylated fused *as*-triazinones, i.e., **11**, **13**, **14**, **15**, **17**, were chosen to examine their ability to increase the levels of apoptotic caspases (caspase-3, -7 and -9) in tumour cells of the lung (A549), cervix (HeLa) and breast (T47D), as well as in normal (GMK) cells (Table 2). In this case, three human caspase ELISA kits (CASP3, CASP7, CASP9) were applied with antibodies specifically recognising caspase-3, caspase-7 and caspase-9. The measured levels of these caspases are reported herein in comparison with the untreated control.

The results shown in Table 2 indicate that the cytotoxic activity of fluorophenylated small molecules can be attributed to their ability to increase the levels of apoptotic caspases in human tumour cells of epithelial origin. Caspase-3, -7 and -9 levels were significantly enhanced in human tumour cells of the lung, cervix and breast after exposure to the majority of tested compounds. The most potent activators of these apoptotic caspases were molecules **15** and **17**, which significantly increased each caspase level in each cell line used. It was found that caspase-3 levels are increased after incubation with **15** and **17** in A549 cells by 67 and 69%, respectively, and in HeLa cells by 97 and 83%, respectively. In T47D cells, the levels of caspase-3 were elevated by 83–87% after incubation with **13**, **14**, **15** and **17**. It was shown that in A549 cells caspase-7 levels are increased by 97–98% after incubation with **13**, **14**, **15** and **17**. In turn, in HeLa cells the levels of caspase-7 were found to be enhanced by 104–106% after incubation with each compound tested, while in T47D cells they were increased by 102 and 104% after incubation with **11** and **13**, respectively, and by 203% after incubation with **14**, **15** and **17**. It was proven that caspase-9 levels are increased in A549 cells by 97% after incubation with **11** and by 191–193% after incubation with **13**, **14**, **15** and **17**, in HeLa cells by 193–196% after incubation with each compound tested, and in T47D cells by 49% after incubation with **11** and **13** as well as by 98–99% after incubation with **14**, **15** and **17**. In turn, none of the tested molecules (**11**, **13**, **14**, **15**, and **17**) had any effect on the levels of caspases 3 and 9 in normal GMK cells. Only compounds **14**, **15**, and **17** enhanced caspase-7 level in GMK cells.

In conclusion, fluorophenylated fused *as*-triazinones **11**, **13**, **14**, **15** and **17** significantly elevate apoptotic caspase levels in human lung, cervical, and breast cancer cells. Nevertheless, these preliminary findings do not provide direct evidence that the antiproliferative effects of these compounds are directly related to caspase activation. Therefore, confirming apoptosis requires comprehensive mechanistic assays, such as Annexin V/PI flow cytometry or TUNEL staining.

### 2.5. Molecular Targets for Fluorophenylated Fused 1,2,4-Triazinones (***10**–**18***)

According to the SwissSimilarity web tool (https://www.swisssimilarity.ch/; accessed on 20 August 2026), none of the evaluated fluorophenylated fused 1,2,4-triazinones (**10**–**18**) exhibited a Tanimoto coefficient > 0.7, indicating the lack structural similarity to existing approved or investigational drugs. This suggests that their mode of action would be mechanistically unrelated to that of currently known agents.

Adenosine, a purine nucleoside, regulates diverse processes by signalling through adenosine receptors (ARs), which belong to the G-protein-coupled receptor (GPCR) superfamily. Tumour-derived adenosine can accumulate to immunosuppressive levels within the surrounding microenvironment. This drastic concentration increase—from nanomolar to micromolar levels—is due to the activation of the CD39 (nucleoside triphosphate dephosphorylase) and CD73 (ecto-5′-nucleotidase) enzymes. Consequently, elevated extracellular adenosine within the tumour microenvironment promotes tumour progression by signalling through the adenosine A_2A_ and A_2B_ receptors, which represent primary targets in cancer immunotherapy. In solid tumours, this signalling molecule acts as an immunosuppressive shield via two distinct pathways: the high-affinity A_2A_ receptor suppresses T cells, natural killer cells, and dendritic cells, whereas the low-affinity A_2B_ receptor promotes angiogenesis, myeloid-derived suppressor cell recruitment, and metastasis. Therefore, dual targeting of these receptors represents a promising therapeutic strategy, as blocking these pathways restores the anti-tumour immune response [31,32,33,34].

**Figure 2:**
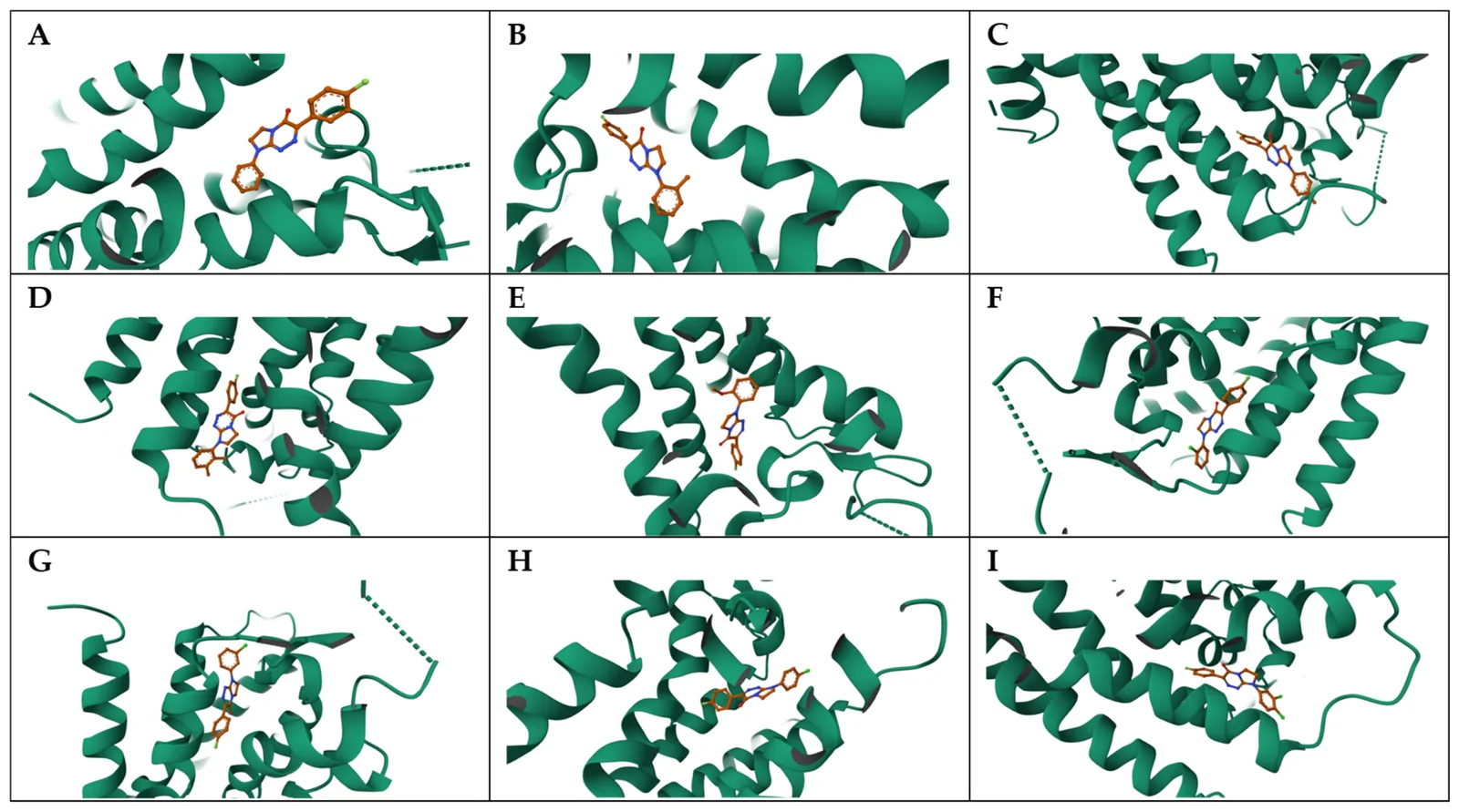
Three-dimensional (3D) binding poses (**A**–**I**) of fluorophenylated fused 1,2,4-triazinones (**10**–**18**) within the binding pocket of the adenosine A_2A_ receptor (PDB ID: 3EML).

**Figure 3:**
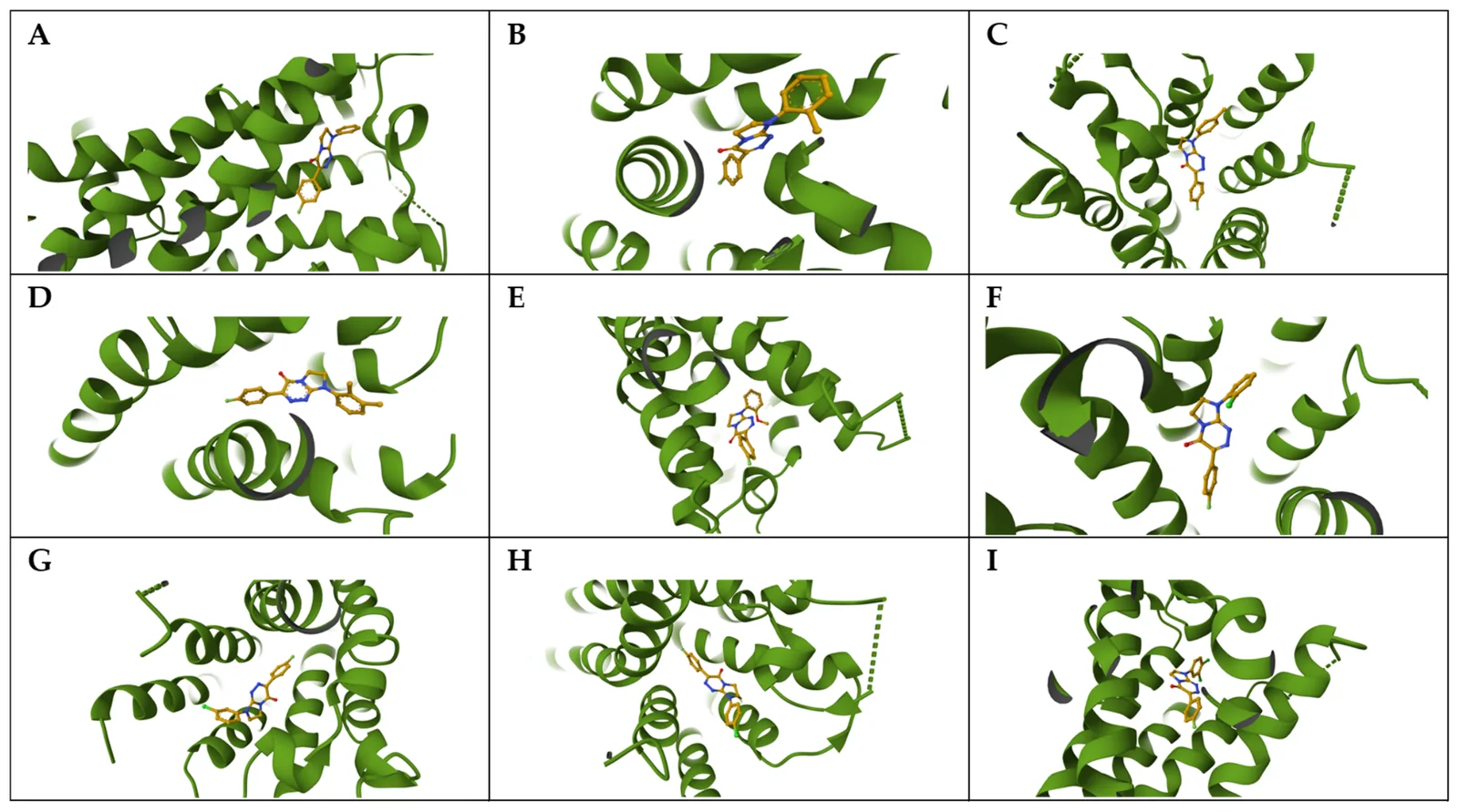
Three-dimensional (3D) binding poses (**A**–**I**) of fluorophenylated fused 1,2,4-triazinones (**10**–**18**) within the binding pocket of the adenosine A_2B_ receptor (PDB ID: 8HDO).

Previous results of extensive biological studies allowed for the identification of both 7,8-dihydroimidazo[2,1-*c*][1,2,4]triazin-4(6*H*)-one, described by Szuster-Ciesielska et al. [35], and benzo[*d*]imidazo[2,1-*c*][1,2,4]triazin-4(6*H*)-one—systematically named as [1,2,4]triazino[4,3-*a*]benzimidazol-4(10*H*)-one by Taliani et al. [36]—as novel substituted privileged scaffolds capable of interacting with human adenosine A_2A_ and A_2B_ receptors, respectively. These findings provide a strong rationale for the in silico molecular docking of biologically active fluorophenylated dihydroimidazotriazinones with modified substituents at the *N*8. Sharing structural similarity with the aforementioned A_2A_ and A_2B_ AR antagonists based on dihydroimidazotriazinone and benzo[*d*]imidazotriazinone cores [35,36], these molecules may potentially occupy the binding sites of both adenosine receptors, and thereby block endogenous adenosine signalling. Additionally, the SwissTargetPrediction web tool (https://www.swisstargetprediction.ch/; accessed on 20 August 2026) identified class A G-protein-coupled receptors, including the A_2A_ and A_2B_ ARs, as molecular targets for these compounds. Therefore, a series of novel heterocyclic small molecules (**10**–**18**)—featuring a drug-like 3-(4-fluorophenyl)-7,8-dihydroimidazo[2,1-*c*][1,2,4]triazin-4(6*H*)-one skeleton with either an unsubstituted phenyl moiety or various aromatic ring substituents—was docked into the orthosteric binding site of the adenosine A_2A_ (PDB ID: 3EML) [37] and A_2B_ (PDB ID: 8HDO) [38] receptors (Figure 2 and Figure 3). This was performed to evaluate the ligand-receptor binding affinity, structural complementarity and the molecular basis of interactions. Additionally, molecular docking studies were carried out for the selective A_2A_ AR antagonist ZM-241385 and the selective A_2B_ AR antagonist PSB-1115. The docking score values (in kcal/mol) were calculated for each compound against both receptors, and subsequently compared with those of reference antagonists, i.e., ZM-241385 and PSB-1115.

**Figure 4:**
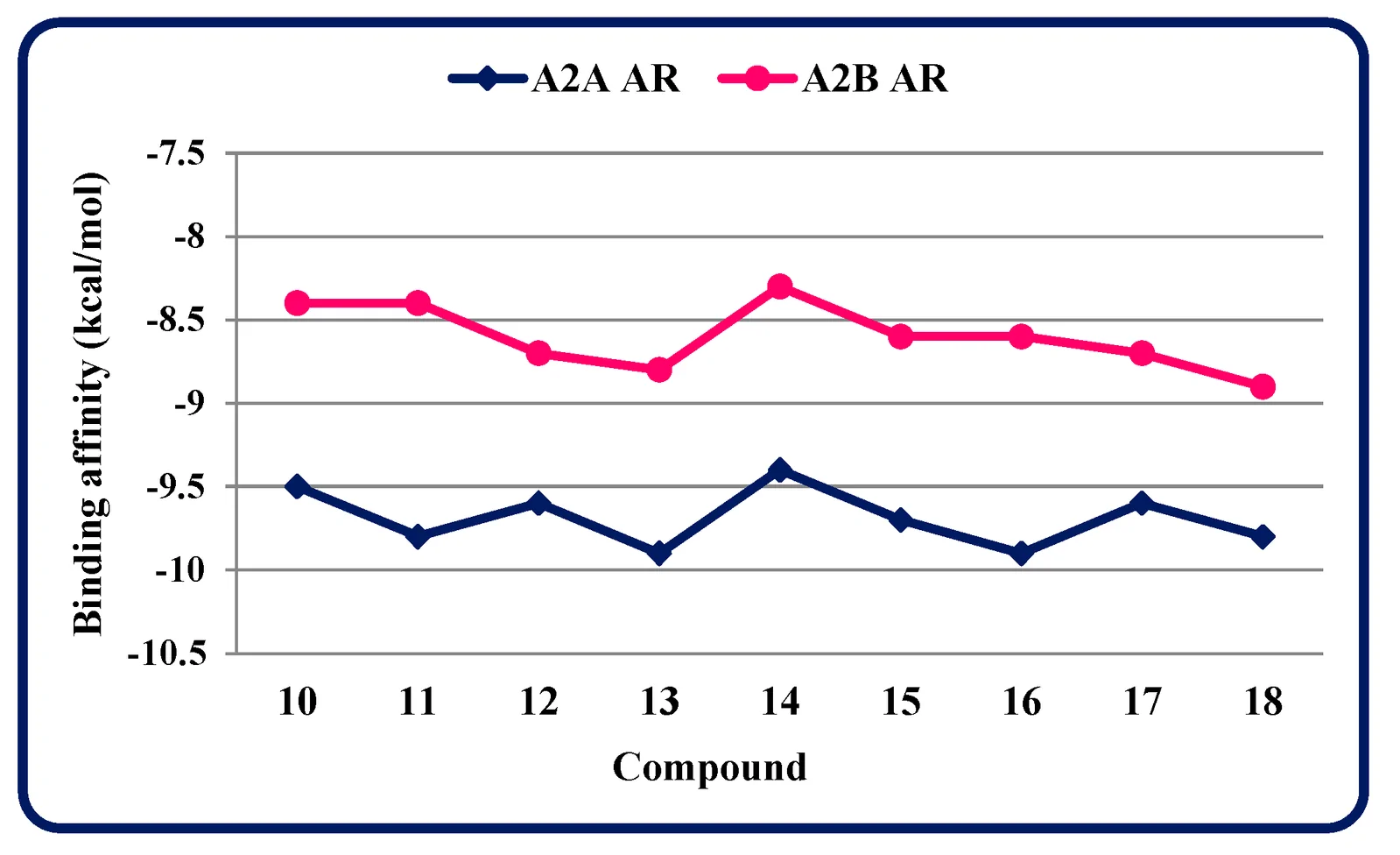
Comparison of calculated binding affinities of fluorophenylated fused 1,2,4-triazinones (**10**–**18**) for adenosine A_2A_ (PDB ID: 3EML) and A_2B_ (PDB ID: 8HDO) receptors.

In silico docking studies performed with the AutoDock Vina 1.2.0 [39,40] engine integrated into the Tamarind Bio platform (https://www.tamarind.bio/; accessed on 22 August 2026) revealed that structural modifications within a series of fluorophenylated fused 1,2,4-triazinones (**10**–**18**) significantly modulated their binding affinities for the adenosine A_2A_ and A_2B_ receptors (Table 3, Figure 4).

Remarkably, all investigated derivatives demonstrated high affinity for both the A_2A_ and A_2B_ AR subtypes, displaying docking score values of ≤−8.3. Notably, consistently greater binding affinities—ranging from −9.9 to −9.4 kcal/mol—were observed for the adenosine A_2A_ receptor (Table 3, Figure 4). When compared to the reference selective A_2A_ AR antagonist, ZM-241385, all evaluated molecules exhibited superior binding affinities. The unsubstituted derivative **10**, serving as the parent structure, revealed a baseline affinity of −9.5 kcal/mol. Modifying the phenyl ring with lipophilic, electron-donating, or electron-withdrawing groups distinctly affected binding affinity for this receptor. This highlights the sensitivity of the A_2A_ binding pocket to both steric and electronic effects. Introducing a single methyl group at the *ortho* position in **11** or the *para* position in **12** improved the binding affinity compared to **10**. This suggests that the extended hydrophobic pocket comfortably accommodates or favourably binds with small alkyl groups. Remarkably, a beneficial effect was observed upon ring activation with electron-donating groups of low steric hindrance, as evidenced by the dimethylated derivative **13**. Its high affinity for the A_2A_ AR suggests that small, electron-donating methyl groups at the *ortho* and *meta* positions optimise hydrophobic contacts within the receptor cavity. The incorporation of a chlorine atom significantly enhanced A_2A_ AR binding; indeed, the *meta*-chlorinated derivative **16** reached an identical binding affinity to the promising dimethyl analogue **13**. Placing a chlorine atom at the *ortho* position (**15**) slightly reduced affinity, while introducing a chloro group at the *para* position (**17**) matched the affinity of 4*-*methyl analogue. Interestingly, introducing chlorine atoms at both the *meta* and *para* positions in **18** did not produce a synergistic effect; instead, it resulted in a lower binding affinity for A_2A_ AR than that of the promising mono-substituted 3-chloro derivative. Conversely, introducing a bulky and strongly electron-donating methoxy group at the *ortho* position resulted in a drop in the binding affinity of **14**, suggesting steric hindrance within the receptor-binding pocket. Nevertheless, this molecule still exhibits superior binding affinity compared to the selective antagonist of A_2A_ AR, ZM-241385. The high binding affinity of the chlorinated analogues, particularly **16** (3-Cl) and **15** (2-Cl), underscores the beneficial role of halogen atoms, which is likely driven by favourable halogen bonding or localised electrostatic interactions.

The studied compounds were characterised by a lower binding affinity for the adenosine A_2B_ receptor, with docking score values ranging from −8.9 to −8.3 kcal/mol (Table 3, Figure 4), although structural modifications offered crucial SAR insights. Nevertheless, the majority of compounds outperformed the selective A_2B_ AR antagonist PSB-1115. Notably, the 3,4-dichloro derivative (**18**) achieved the strongest binding affinity for the A_2B_ AR, closely followed by the 2,3-dimethyl analogue (**13**). This indicates that this receptor pocket can accommodate either highly polarisable or highly hydrophobic substituents. In the design of ligands targeting the adenosine A_2B_ receptor within this class of molecules, introducing mono-substituted groups at various positions of the phenyl moiety has proven to be a successful strategy. Specifically, substitution with a methyl group at the *ortho* or *para* positions (**11** and **12**, respectively) or chlorine atom at the *ortho*, *meta* and *para* positions (**15**, **16**, and **17**, respectively) resulted in comparable binding affinity values, which were nonetheless superior to the value of PSB-1115. The sole exception to this trend was compound **14** (bearing a 2-OCH_3_ group), which demonstrated equipotency to the reference antagonist. Notably, this derivative exhibited the lowest binding affinity for the A_2B_ AR among all synthesised molecules. This indicates that the presence of a bulky, electron-donating methoxy group at the *ortho* position introduces steric hindrance or unfavourable electronic repulsions that disrupt binding.

Regarding the binding affinity within the fluorophenylated fused 1,2,4-triazinone series, compounds **13** and **16** proved to be the most promising as ligands targeting A_2A_ ARs. Notably, both molecules retained sufficient affinity for the A_2B_ subtype to exhibit a dual antagonistic profile. This dual antagonism justifies their further in vitro and in vivo evaluation in cancer immunotherapeutic applications. Structural analysis within this class of compounds shows that disubstitution with small alkyls of low steric hindrance (such as 2,3-(CH_3_)_2_) (**13**) or monosubstitution with chlorine at the *meta* position (**16**) or the *para* position (**17**) of the phenyl moiety especially maximises interactions with the adenosine A_2A_ receptor. Similarly, the A_2B_ AR prefers hydrophobic disubstituted phenyl rings, such as the 3,4-Cl_2_ group in **18** and 2,3-(CH_3_)_2_ group in **13**, for antagonism. Compounds **13**, **16** and **17** may be regarded as dual antagonists, although they exhibit a distinctly stronger binding affinity for the A_2A_ AR than for the A_2B_ AR. These findings suggest that incorporating either an electron-donating dimethyl group at the 2,3-position or an electron-withdrawing chloro group at the *meta* or the *para* position of the phenyl ring is a viable strategy for designing potent dual antagonists based on the dihydroimidazotriazinone core targeting both A_2A_/A_2B_ receptor subtypes. While selective antagonists are currently undergoing clinical trials focussed solely on blocking the adenosine A_2A_ receptors [41], dual A_2A_/A_2B_ AR antagonists—which block both receptors simultaneously, as exemplified by muvadenant—may offer higher therapeutic efficacy by concurrently counteracting the immunosuppressive and pro-angiogenic effects of adenosine [42,43].

**Figure 5:**
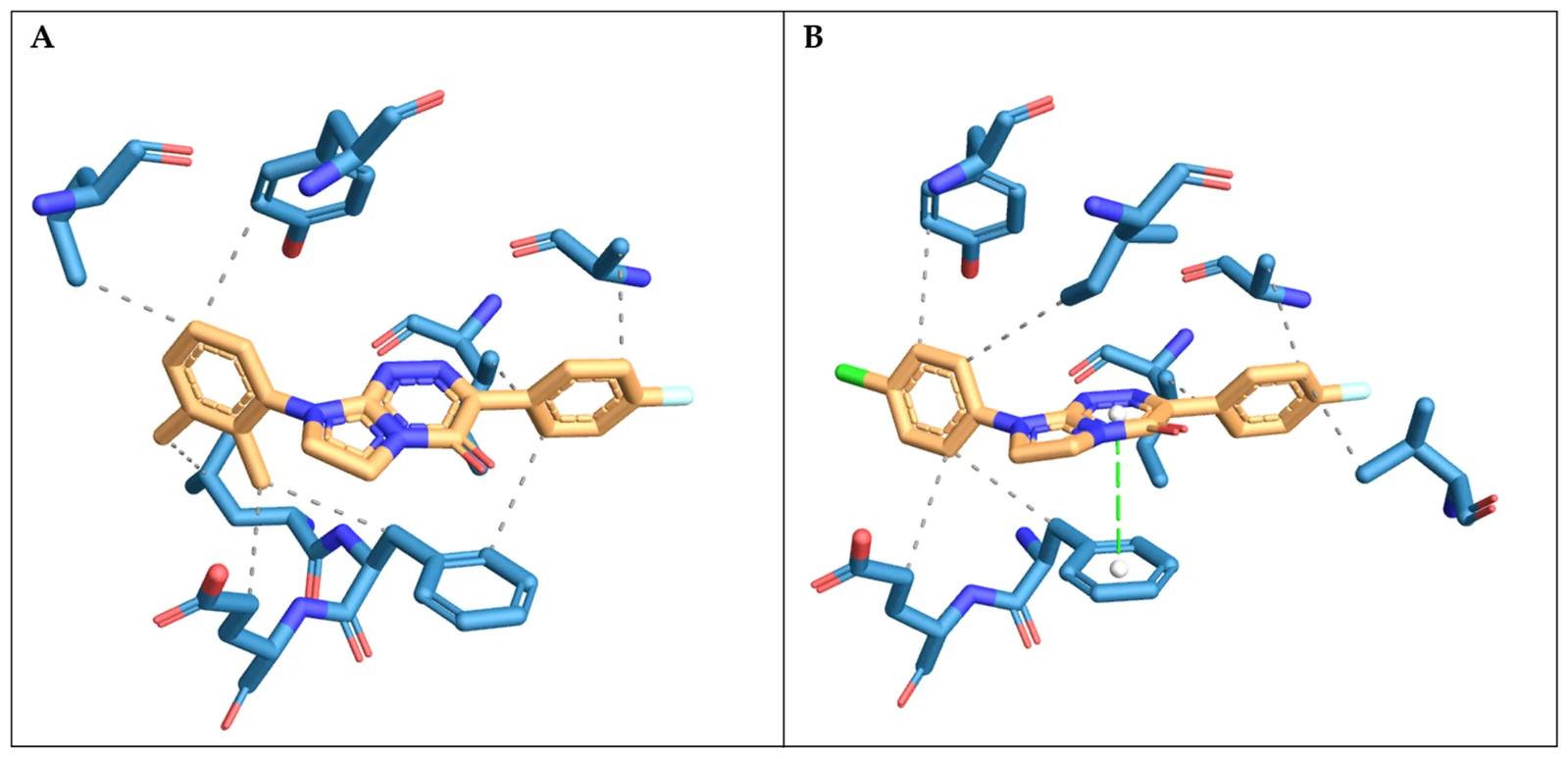
Representative complexes—of ligands **13** (**A**) and **17** (**B**) within the adenosine A_2A_ receptor (PDB ID: 3EML)—showing the highest number of contacts. Interaction lines were automatically generated by the Protein-Ligand Interaction Profiler (PLIP): grey dashed lines indicate hydrophobic interactions, and green dashed lines represent π-π stacking.

To gain further insight into the ligand–receptor interactions, the docked complexes of ligands **10**–**18** within the orthosteric binding sites of the adenosine A_2A_ (PDB ID: 3EML) and A_2B_ (PDB ID: 8HDO) receptors were analysed using the Protein-Ligand Interaction Profiler (https://plip-tool.biotec.tu-dresden.de/plip-web/plip/index; accessed on 24 August 2026) [44]. Interaction profiling revealed key non-covalent contacts that stabilise ligand binding within the receptor active sites. Table 4 summarises the key interactions between the studied ligands and amino acid residues, along with their geometric parameters. Figure 5 shows representative complexes displaying the highest number of contacts.

Protein–ligand interaction analyses demonstrated that the stabilisation of ligands **10**–**18** within the A_2A_ AR structural template occurs primarily through hydrophobic contacts with key amino acid residues located on the transmembrane (TM) helices and extracellular loop 2 (ECL2). The upper portion of the pocket is stabilised by ECL2; specifically, ligands **11**–**18** interact with Phe168, a residue acting as an aromatic “lid” over the orthosteric site, whereas ligands **11**, **13**, **14**, **16**, and **17** engage with Glu169, forming strong contacts with polar or charged atoms of the ligands. These amino acid residues define the geometry of the pocket entrance, thereby preventing complex dissociation or blocking access to endogenous adenosine—a highly desirable feature for antagonists [37,45]. Notably, compound **17** additionally forms a classical, parallel-displaced π-stacking interaction with Phe168 (centroid-to-centroid distance: 4.19 Å), which represents a hallmark structural feature of high-affinity A_2A_ AR blockers [37,46]. Deep penetration and anchoring of the ligand core are demonstrated by hydrophobic interactions between ligands **10**, **11**, **13**–**18** and Tyr271 located on TM7. Notably, molecule **15** exhibits a particularly close contact of 3.37 Å. This spatial proximity enables the phenolic hydroxyl group of Tyr271 to engage in strong, short-range electrostatic interactions or potential hydrogen bonds with polar acceptors on the ligand core, effectively stabilising the ligand within the binding site of the receptor. Lateral anchoring within the hydrophobic core is further supported by the aliphatic residues of Ala63 on TM2 (for ligands **10**–**18**) and Val84 on TM3 (for ligands **10**–**12**, **14**, and **16**–**18**), which participate in strong lipophilic interactions with the core scaffold of the docked derivatives. Additionally, a cluster of branched aliphatic residues—specifically Leu167 (for **12**), Leu267 (for **15**), Ile66 (for **12**, **13**, and **15**–**18**), and Ile274 (for **10**–**12**, and **17**)—exerts strict spatial confinement. In particular, the multivalent interactions established with Leu167 by **12** signify an optimal fit within the binding cavity. This comprehensive interaction profile ensures tight molecular packing and a physical blockade of the receptor activation channel, directly correlating with the intended antagonistic mechanism of action [37,47].

Molecular docking studies showed high geometric complementarity of compounds **10**–**18** within the A_2B_ AR structural template, mimicking the general binding mode observed in A_2A_ AR. Similarly, these ligands are firmly anchored to the transmembrane helices and extracellular loop 2. The stabilisation mediated by ECL2 is effectively reproduced through hydrophobic contacts between Phe173 and ligands **13**, **16**, and **17**, alongside interactions of Leu172 with ligands **10**, **12**, **13**, and **16**–**18**, indicating tight and stable molecular packing at the channel entrance. The lateral anchoring of ligands **10**, **12**, **16**, and **17** is preserved via Ile67 on TM2. Notably, ligands **10**, **11**, and **13**–**16** exhibit high adaptability to subtype-specific sequence variations in the deeper layers of TM7; instead of forming the polar contacts with tyrosine observed in A_2A_ AR, they engage in purely lipophilic interactions with Ile276 in A_2B_ AR. Furthermore, within the 8HDO structure, ligands **16**–**18** establish contacts with Trp247 (on TM6), a crucial molecular switch for receptor activation. These compounds exert their antagonistic effect on the A_2B_ AR through a steric blockade mechanism, physically restricting the rotational freedom of helix 6 required for downstream signal transduction. Additionally, ligands **10**–**14**, **17**, and **18** form hydrophobic contacts with Val250 (with compound **14** establishing a multivalent contact) on TM6, in close proximity to the activation microdomain. Interestingly, compound **14** forms a highly specialised π-cation interaction between its guanidine moiety and the aromatic ring of Phe173 (perpendicular distance: 4.85 Å). In structural biology, π-cation interactions are recognised as potent energy-contributing factors that frequently exceed the stabilisation energy of standard hydrophobic contacts. This strongly suggests that this compound achieves high-affinity binding to A_2B_ AR not through extensive lipophilic packing, but via charge-enhanced anchoring to ECL2 [48].

In summary, the comparative analysis of binding modes highlights the structural distinctiveness of the compounds within the series of fluorophenylated fused *as*-triazinones, which is conditioned by a dense, multi-layered network of interactions. Among the studied molecules, ligands **13**, **16**, and **17** are identified as the most promising dual-target candidates, exhibiting the most comprehensive and potent binding profiles for both adenosine A_2A_ and A_2B_ receptor subtypes. These compounds efficiently engage a triad of critical stabilising domains: ECL2 via Phe168/Phe173, the hydrophobic core spanning TM2 and TM3, and the activation microdomain on TM6. Within the A_2A_ AR binding cavity, ligand **17** stands out as the most potent antagonist, demonstrating exceptionally high structural affinity. This potency is fundamentally driven by a classic, parallel-displaced π-π stacking interaction established with the aromatic “lid” residue Phe168, while simultaneously maintaining an extensive network of seven independent intermolecular contacts. Conversely, ligand **14** introduces a unique binding mode within the adenosine A_2B_ receptor structural template, diverging from the extensive, lipophilic packing driven by the hydrophobic effect seen in the remaining compounds. Instead, ligand **14** utilises a highly specialised, charge-enhanced π-cation interaction between its guanidine moiety and the aromatic side chain of Phe173 on ECL2. This interaction functions as a powerful polar anchor at the channel entrance, providing an alternative energetic driver for high-affinity receptor recognition [48,49]. The functional efficacy of this series of fluorophenylated dihydroimidazotriazinones is further enhanced by a direct steric blockade of the A_2B_ AR activation microdomain. In particular, ligands **16**, **17**, and **18** form direct contacts with Trp247 (on TM6), which serves as a pivotal conformational switch. By physically restricting the rotational and outward movement of helix 6 essential for the downstream signalling cascade, these compounds effectively lock the receptor in its inactive conformation, providing a clear structural rationale for their potent antagonistic mechanism of action.

### 2.6. The Effect of Fluorophenylated Fused 1,2,4-Triazinones (***10**–**18***) on Red Blood Cells

**Figure 6:**
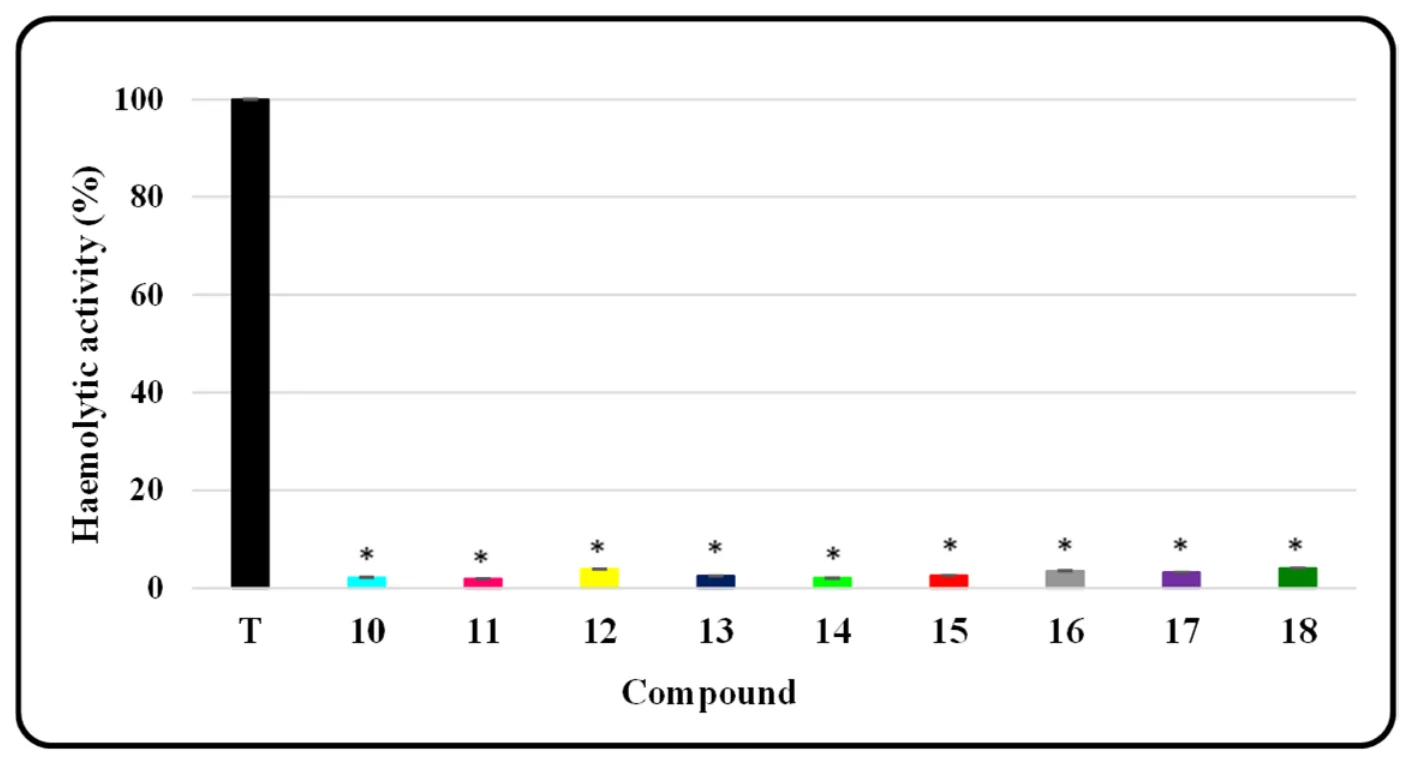
Haemolytic activity of fluorophenylated fused triazinones (**10**–**18**). T—triton X-100 (a positive control). Compounds and triton X-100 were tested at a concentration of 150 μM. Data (from three independent experiments) are shown as the mean and standard deviation. * statistically significantly different from the control (*p* < 0.05, Student’s *t*-test).

Assessing the haemolytic potential of newly synthesised compounds is an important component of toxicity profile screening in the early preclinical phase of drug development. Erythrocytes are among the most commonly used models for studying cytotoxicity because, as the most abundant blood cells, they are readily accessible for research. Their cell membranes are similar to the membranes of other cells. Therefore, the effect of potential drug candidates on red blood cells may be a marker of their overall cytotoxicity towards normal cells or—as in the case of injectable active substances—haemocompatibility is a direct indicator of their toxicity [50,51,52,53]. Taking this into account, the effect of new fluorophenylated fused *as*-triazinones (**10**–**18**) on erythrocytes was investigated in an ex vivo model of erythrocytes (Figure 6).

The results of the haemocompatibility study showed a very good safety profile of these compounds. As shown in Figure 6, incubation of red blood cells with tested molecules at a concentration of 150 μM did not promote any significant haemolytic effects (haemolysis rate below 5%), which indicates that these compounds are safe for erythrocytes. In contrast, triton X-100 (a non-ionic surfactant used as a haemolytic agent) induced complete lysis of red blood cells. It is worth noting that the investigated compounds are completely non-toxic to erythrocytes at a concentration at which they effectively inhibit the in vitro proliferation of various cancer cells. This is a valuable observation, especially since these molecules may prove to be potential therapeutic agents in the treatment of cancer.

### 2.7. Antihaemolytic Activity of Fluorophenylated Fused 1,2,4-Triazinones (***10**–**18***)

**Figure 7:**
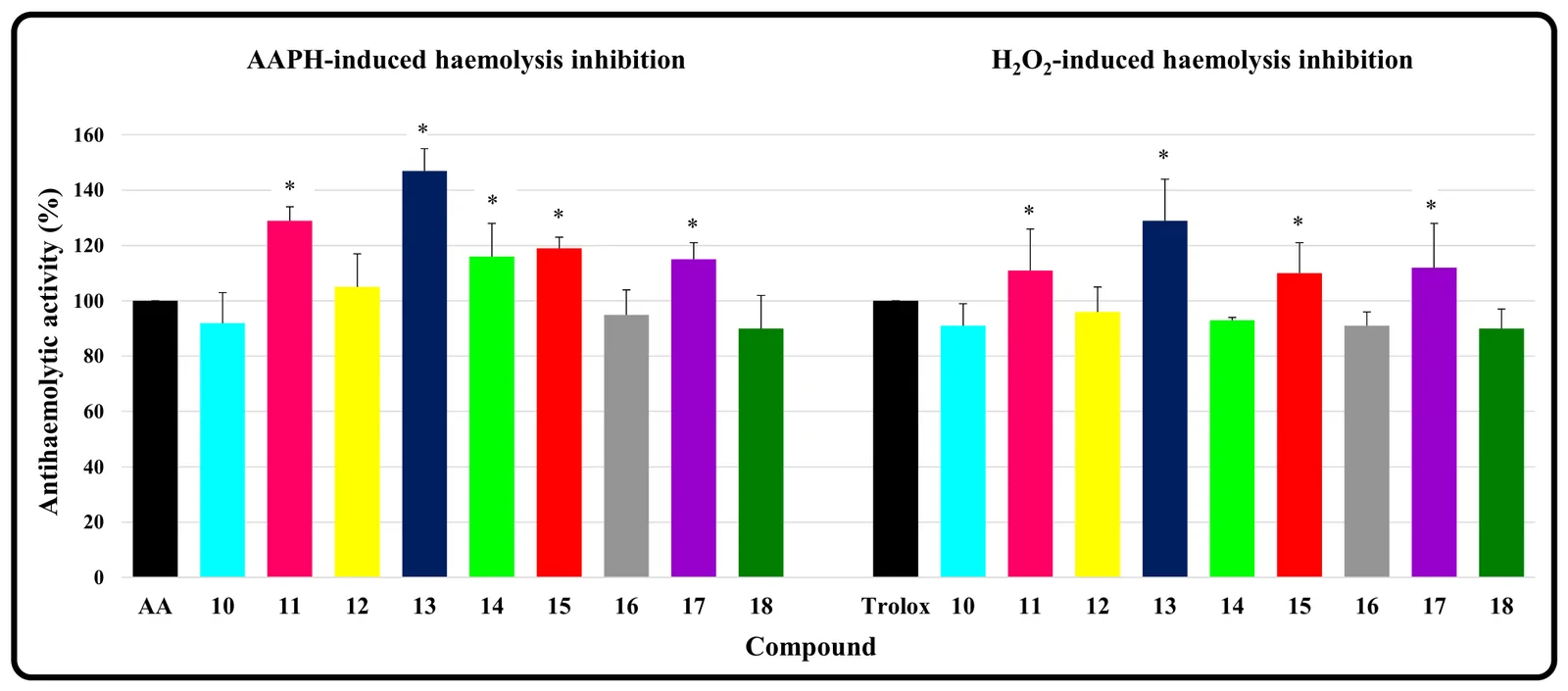
Antihaemolytic activities of fluorophenylated fused 1,2,4-triazinones **10**–**18** in relation to antioxidant standards, i.e., ascorbic acid and trolox. AAPH—2,2′-azobis(2-methylpropionamidine)dihydrochloride; AA—ascorbic acid; H_2_O_2_—hydrogen peroxide; trolox—6-hydroxy-2,5,7,8-tetramethylchroman-2-carboxylic acid. All compounds and standard antioxidants (AA and trolox) were tested at a concentration of 150 μM. Data (from four independent experiments) are shown as the mean ± standard deviation. *—the activity of compound statistically significantly higher than that of an antioxidant standard (*p* < 0.05, Student’s *t*-test).

Since none of the new fluorophenylated fused *as*-triazinones (**10**–**18**) had an adverse effect on red blood cells, as they did not cause haemolysis, it was purposeful to investigate whether these compounds exhibit antihaemolytic activity. The study was performed ex vivo on oxidatively stressed erythrocytes. In this model, reactive oxygen species (ROS), such as peroxyl radicals derived from 2,2′-azobis(2-amidinopropane) dihydrochloride (AAPH) or hydrogen peroxides, induce lipid peroxidation and oxidation of membrane proteins, leading to erythrocyte membrane damage and ultimately haemolysis. The tested compounds, by scavenging ROS, protect red blood cells from damage caused by oxidative stress and thus prevent haemolysis [54]. In this assay, the ability of congeners **10**–**18** to protect erythrocytes from oxidative haemolysis was examined and compared with the activity of standard antioxidants such as ascorbic acid or trolox. As shown in Figure 7, all molecules were able to protect red blood cells from oxidative damage, and the inhibition of AAPH- or H_2_O_2_-generated haemolysis was structure-dependent. It can be clearly seen that the most effective in inhibiting haemolysis induced by AAPH were compounds **11**, **13**, **14**, **15** and **17**, and by H_2_O_2_—**11**, **13**, **15** and **17**, whose antihaemolytic activity was significantly higher than that of ascorbic acid or trolox, respectively. The remaining derivatives protected red blood cells against oxidative haemolysis as effectively as standard antioxidants. Thus, *ortho*-methyl, *ortho*-methoxy, *ortho*-chloro, 2,3-dimethyl and *para*-chloro substituents at the phenyl ring are the best substitution patterns with respect to antihaemolytic activity of this novel class of compounds.

The research results prove that the developed fluorophenylated fused *as*-triazinones are not only safe for erythrocytes, but also have a protective effect against oxidative damage to the erythrocyte membrane, which makes them safe drug candidates (especially compounds **11**, **13**, **14**, **15** and **17**) suitable for further investigations.

### 2.8. Toxicity Risk Assessment of Fluorophenylated Fused 1,2,4-Triazinones (***10**–**18***)

An OSIRIS Property Explorer (accessed on 27 January 2026) [55] was employed to assess the risk of adverse side effects of fluorophenylated fused *as*-triazinones (**10**–**18**) (Table 5). This software predicts whether the structures developed may be mutagenic, carcinogenic, irritating, or reprotoxic. The prediction relies on substructural “genotoxicophoric” fragments encountered in each original molecule that give rise to toxicity alerts from the Registry of Toxic Effects of Chemical Substances enlarged database.

For the vast majority of molecules (**10**–**12** and **14**–**18**), no risk of mutagenicity, tumorigenicity, or irritating and reprotoxic effects were predicted because of the lack of “genotoxicophore” fragments in their structures. The risk of irritation was predicted only for compound **13**, the molecule of which contains a substructural fragment of 2,3-dimethylphenylnitrene, presumably associated with 2,3-dimethylaniline (2,3-xylidine). Exposure to 2,3-xylidine may cause irritation of eyes and skin [56]. This fragment is found in the non-steroidal anti-inflammatory drug mefenamic acid (synthesised from 2-chlorobenzoic acid and 2,3-dimethylaniline), which does not affect its therapeutic application [56,57]. Noteworthy is that the risk of tumorigenicity has been predicted for the anticancer drug gemcitabine. This is also confirmed by in vitro studies [58,59]. The results are listed in Table 5, where a value of 1.0 indicates no adverse side effect, while a lower value (0.6) means an increased risk of an adverse side effect.

### 2.9. In Silico ADME Evaluations of Fluorophenylated Fused 1,2,4-Triazinones (***10**–**18***)

Poor pharmacokinetic properties usually lead to drug candidate failure. Therefore, ADME (absorption, distribution, metabolism, excretion) evaluations should be performed as early as possible in the preclinical phase of drug development [60,61]. Molecular descriptors for drug-likeness assessment as well as parameters characterising the ADME profile of fluorophenylated fused *as*-triazinones **10**–**18** were calculated in silico using the pkCSM software (accessed on 27 January 2026) [62], and are presented in Table 6 and Table 7.

The rule of five (Ro5) is used to assess whether small molecules under investigation, due to their drug-like properties, have sufficient oral bioavailability to cross cell membranes by passive diffusion. It is seen that compounds **10**–**18** have structural features required by Lipinski’s Ro5: their molecular weights (ranging from 308.316 g mol^−1^ to 377.206 g mol^−1^) are less than 500, the numbers of hydrogen bond donor (HBD = 0) and hydrogen bond acceptor (HBA = 5 or 6) groups are ≤5 and 10, respectively, and their log *P*_o/w_ values—which are a measure of their hydrophobicity (in the range from 2.5961 to 3.9029)—are <5 [63] (Table 6). So, in the case of these compounds, none of the criteria of this rule were violated. In addition, all molecules obey the requirements formulated by Veber et al. for pharmaceuticals with acceptable oral bioavailability. The number of hydrogen bond donors and acceptors in total (HBD + HBA) is 5 or 6, so it is ≤12, and the number of rotatable bonds (nRB) is 2 or 3, so it is ≤10 [64] (Table 6). Thus, the molecular descriptor values given above indicate that compounds **10**–**18** are drug-like and meet the requirements for medicines with acceptable oral bioavailability and optimal lipophilicity.

In silico ADME prediction models allow the assessment of the pharmacokinetic profiles of new drug candidates. The pkCSM software [62] provides extensive information on selected molecules at an early stage of drug development. Using this program, the parameters responsible for absorption (intestinal absorption, Caco2 permeability, interactions with P-glycoprotein, water solubility, and skin permeability), distribution (steady state volume of distribution, fraction unbound in human blood plasma, and permeability to the blood-brain barrier and central nervous system), metabolism (interactions with cytochrome P450 isoforms), and excretion (total clearance, and renal transport systems) were calculated for the title molecules (Table 7).

Compounds with high intestinal absorption and permeability rates penetrate more efficiently into the bloodstream and, with it, into target tissues, where they can exert the desired pharmacological effect [61,65]. Molecules **10**–**18** show high intestinal absorption values (HIA: from 96.180 to 99.474%) and good passive diffusion potential (P_Caco-2_: from 1.107 to 1.439 × 10^−6^ cm s^−1^), which indicates that they should be efficiently absorbed through intestines and readily penetrate into the systemic circulation. Interaction with P-glycoprotein (P-gp) plays an important role in the absorption of drug candidates. As a carrier protein found in intestinal epithelial cells, P-gp can limit the absorption of its substrates by removing them from the cell [66,67]. None of compounds **10**–**18** were identified as P-glycoprotein substrates, suggesting that their penetration into the systemic circulation may be more efficient. Furthermore, all molecules were found to be inhibitors of P-gp II, and **15**–**18** also of P-gp I, indicating that they may potentially enhance their own absorption by inhibiting the activity of this carrier protein. The absorption process of compounds is also influenced by their level of solubility in water [61]. The predicted log *S* value for more than 80% of drugs is above −4 [55]. Most of our fluorophenylated fused *as*-triazinones also have values of this parameter > −4. Skin permeability is a factor to consider when administering pharmaceuticals, but it also helps determine the risks associated with their use [68,69]. Log *K*_p_ values allow for the assessment of the absorption potential of active substances through the skin. Molecules **10**–**18** are not expected to penetrate the skin well, as evidenced by their relatively low logs *K*_p_ (from −2.735 to −2.554). For this reason, they are unlikely to be suitable for transdermal drug delivery. Pemetrexed is poorly available after oral administration due to its low predicted intestinal absorption and passive diffusion potential, as well as unfavourable interactions with P-gp. Gemcitabine has better oral bioavailability. Both drugs penetrate the skin poorly but are water-soluble.

The pharmacological efficacy of a drug depends on its ability to diffuse into tissues and areas where it can be distributed [70]. A parameter estimating fraction unbound in human blood plasma (Fu) in the range of 0.101–0.260 indicates that most of each compound will remain bound to proteins in the circulation. The steady state volume of distribution (VDss) ranging from −0.297 to 0.044 suggests that molecules **10**–**18** will be distributed primarily intravascularly. The log BB values (the logarithmic ratio of the drug concentration in the brain to its concentration in the plasma) between −0.019 and 0.901, as well as the log PS values in the range of −2.379 to −1.891 suggest that the majority of compounds are able to cross blood–brain barrier and some of them are able to reach central nervous system. Thus, these fluorophenylated fused *as*-triazinones can be considered to have therapeutic potential targeting the brain [71,72,73]. Standard drugs, i.e., pemetrexed and gemcitabine, have low VDss and moderate or high Fu, respectively. They are predicted to penetrate the blood–brain barrier poorly and not be distributed in the central nervous system.

The cytochrome P450 (CYP450) enzyme system, found primarily in the liver and intestinal wall, plays an important role in drug metabolism. The CYP3A4, CYP1A2, CYP2C9, CYP2C19, and CYP2D6 isoforms of this enzyme are responsible for the initial phase of metabolism, known as phase I metabolism (oxidation), of over 90% of medicines [61,74,75]. All the compounds under study (**10**–**18**) were identified as substrates of the CYP3A4 isoform and should be subject to normal metabolic processing by this enzyme. Since CYP3A4 is one of the most common CYP450 enzyme systems involved in drug metabolism, these compounds are expected to be easily analysed using pharmacokinetic models and provide predictable metabolic profiles. On the other hand, molecule **18** may autoinhibit CYP3A4, allowing it to remain active in the body longer and extend its half-life, thereby increasing its therapeutic effectiveness. However, compounds **10**–**17**, which do not exhibit CYP3A4 autoinhibition, may provide safer pharmacokinetic profiles by controlling metabolic degradation and promoting balanced elimination. Therefore, these molecules can be considered long-acting agents with no risk of accumulation in the body and reducing the likelihood of side effects. Due to CYP2D6 polymorphism, the interindividual variability between slow-metabolising and fast-metabolising individuals is common for drugs metabolised by this isoform [61]. None of the compounds tested in this study were identified as CYP2D6 substrates or inhibitors, so interindividual differences in the metabolism of these molecules will not be a significant problem, and their pharmacokinetic profiles will be more predictable. The fact that compounds **10**–**18** are not CYP2D6 substrates is an important advantage that may increase their safety, facilitating the optimisation of their doses. This provides a more predictable clinical use. In turn, all compounds were predicted to inhibit CYP1A2, CYP2C19, CYP2C9, indicating a greater likelihood of metabolic interference or drug–drug interactions. Due to their CYP1A2, CYP2C19 and CYP2C9 inhibitory properties, they can be expected to provide therapeutic benefits when combined with certain medications. In contrast, neither pemetrexed nor gemcitabine are predicted to be substrates or inhibitors of any CYP450 isoform.

The duration of action of drugs in the body largely depends on the rate and way of their elimination [61,76]. Total clearance is a key pharmacokinetic parameter that indicates how quickly drugs are removed from the systemic circulation [75,77,78]. The molecules **10**–**18** have total clearance values ranging from −0.287 to 0.004, suggesting a slower elimination process and longer half-life, which may be beneficial in reducing the frequency of dosing. Membrane transporters play an important role in the excretion of drugs, as they are responsible for their movement through the membranes of kidney cells (from the blood to the kidney and from the kidney to the urine). These renal transport systems include, among others, organic cation transporters (OCTs) and organic anion transporters (OATs), which are potential sites of drug interactions in vivo [76]. Most fluorophenylated fused *as*-triazinones were identified as renal OCT2 substrates, suggesting that they would be excreted by the kidneys via active tubular secretion. However, molecule **14**, which is not a substrate of OCT2, will be metabolised primarily in the liver and excreted in the bile, which may be beneficial, especially in cases of renal failure. Pemetrexed and gemcitabine are predicted to have moderate total clearance and are not OCT2 substrates.

In silico assessment of parameters associated with oral bioavailability and ADME profile of fluorophenylated compounds supported their oral drug-likeness and acceptable metabolic liability.

### 2.10. Lipophilicity Indices of Fluorophenylated Fused 1,2,4-Triazinones (***10**–**18***)

Lipophilicity is a relevant parameter from both a physicochemical and pharmacological point of view for potential drug candidates. Passive diffusion of small-molecule drugs transported across biological membranes is dependent on their hydrophobicity [79]. A reversed-phase HPLC method with an ODS-2 column and a suitable isocratic acetonitrile/water eluent system was applied to determine the lipophilicity indices (i.e., logarithmic retention factors—logs *k*, calculated from isocratic retention factors—*k*) of fluorophenylated fused *as*-triazinones (**10**–**18**). This technique was economically cheap since it allowed for a remarkable reduction in the production of effluent wastes (the consumption of acetonitrile—an organic modifier of the mobile phase—was approximately 42 mL during the entire analysis, which means the consumption of 4.6 mL of acetonitrile for free times measuring the retention time of each solute), as well as a relatively short time for the entire analysis (approximately 70 min—taking into account the three-fold measurement of the retention time of each solute).

It should be noted that although the isocratic log *k* values measured in an acetonitrile-water mobile phase are not standardised parameters, they allow the assessment of the lipophilic properties of our compounds and provide a guide for further more detailed tests.

**Figure 8:**
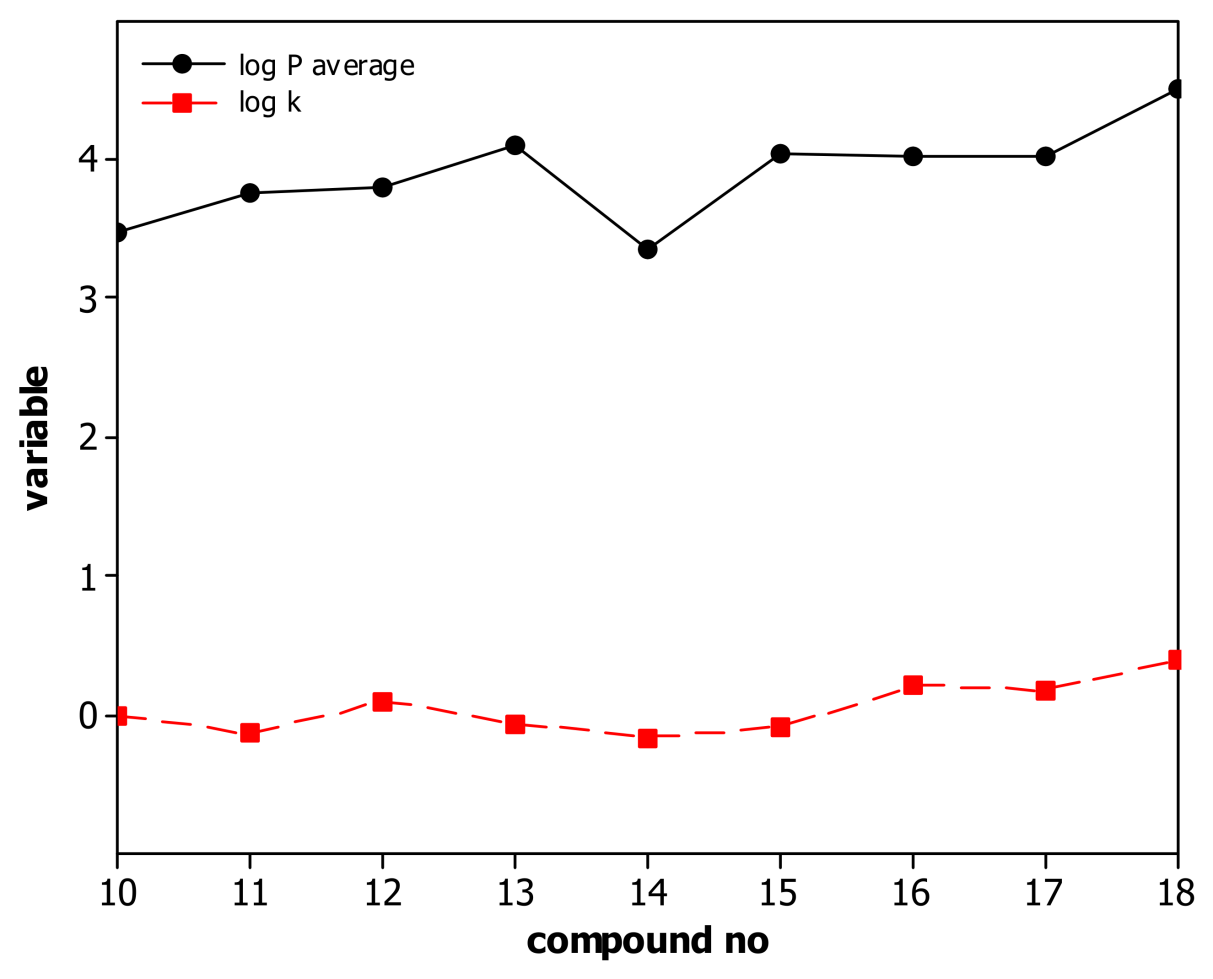
Lipophilicity profiles of all fluorophenylated fused 1,2,4-triazinones (**10**–**18**). Log *P*_average_—the logarithmic *n*-octanol-water partition coefficient computed in silico as an average value from different computational methods for estimating log P (Alog P_s_, AClog P, Alog P, Mlog P, xlog P2 and xlog P3); log *k*—the logarithmic retention factor obtained by RP-HPLC.

Lipophilicity indices—expressed as the logarithmic retention factors (logs *k*) measured in an appropriate mobile phase by RP-HPLC—of molecules **10**–**18**, as well as their average logarithmic *n*-octanol/water partition coefficients (logs *P*_average_), computed in silico, are listed in Table 8. In turn, lipophilicity profiles of compounds are presented in Figure 8.

Since all tested analytes are neutral organic compounds, buffering the mobile phase [83] consisting of water and acetonitrile was pointless. The lipophilicity profile allows for the analysis of experimental lipophilicity indices of the molecules in relation to their structure. It was found that the log *k* values of the tested compounds depend on the substituent (R) attached to the phenyl ring, and decrease in the following order of R: 3,4-Cl_2_ > 3-Cl > 4-Cl > 4-CH_3_ > H > 2,3-(CH_3_)_2_ > 2-Cl > 2-CH_3_ > 2-OCH_3_ (compounds **18**, **16**, **17**, **12**, **10**, **13**, **15**, **11**, **14**, respectively). Compound **18**, containing two electron-withdrawing chlorine atoms, the first in the *meta* position and the second in the *para* position of the phenyl ring, was identified as the most lipophilic molecule—with the longest retention time. In turn, compound **14**, having an electron-donating methoxy substituent in the *ortho* position of the phenyl ring, was revealed as the least lipophilic molecule—with the shortest retention time. Presumably due to the so-called *ortho* effect [84], substituents located in the *ortho* position decrease lipophilicity indices in the following order of R: 2,3-(CH_3_)_2_ > 2-Cl > 2-CH_3_ > 2-OCH_3_ (compounds **13**, **15**, **11**, **14**, respectively). It seems most likely that the placement of substituents at this position hinders the rotational freedom of the phenyl ring, resulting in decreased retention of the *ortho*-substituted molecules (**11**, **13**, **14**, **15**) compared to the retention of the parent structure without a substituent at the phenyl ring (**10**). On the other hand, the same *ortho*-substitutions may also exert a positive mesomeric effect by increasing the electron donor properties of the nitrogen and oxygen atoms (as proton bond acceptors) in the privileged scaffold, which consequently decreases the retention of the analysed substances. It was found that a chloro substituent in the *meta* or *para* position (in the case of **16** and **17**, respectively) and a methyl substituent in the *para* position (in the case of **12**) increased the lipophilicity indices compared to the parent compound (**10**). This increment in log *k* values is consistent with expectations, since the known substituent hydrophobicity constants (π) for Cl and CH_3_ in aromatic substituents are 0.71 and 0.56, respectively [2,80]. Among the monochloro-substituted molecules (**15**, **16**, **17**), the *ortho*-chloro isomer (**15**) is the least lipophilic, while the *meta*-chloro isomer (**16**) is more lipophilic than the *para*-chloro isomer (**17**). Among the methyl-substituted solutes (**11**, **12**, **13**), the *para*-methyl isomer is the most lipophilic, and the *ortho*-methyl isomer (**12**) is the least lipophilic. It was generally noted that among all the compounds analysed, the *ortho*-substituted molecules showed the lowest lipophilicity indices obtained by RP-HPLC.

Analysing the experimentally determined lipophilicity indices (logs *k*) of fluorophenylated fused 1,2,4-triazinones (**10**–**18**) in relation to their biological activity, it can be concluded that the least lipophilic compounds, i.e., **14**, **11**, **15** and **13** (with logs *k* ranging from −0.159 to −0.058), were the least toxic to normal GMK cells, although the lipophilicity index of another—also nontoxic to these cells—compound **17** was 0.182. However, no relationship was found between lipophilicity and antiproliferative activity of the heterocycles tested. The most anticancer active fluorinated *as*-triazinones were characterised by lipophilicity indices in the range from −0.159 to 0.392. In turn, the antihaemolytic activity of the molecules depended on their log *k* values. The compounds with the most effective inhibition of oxidative haemolysis, i.e., **11**, **13**, **14** and **15**, were found to be the least lipophilic.

**Figure 9:**
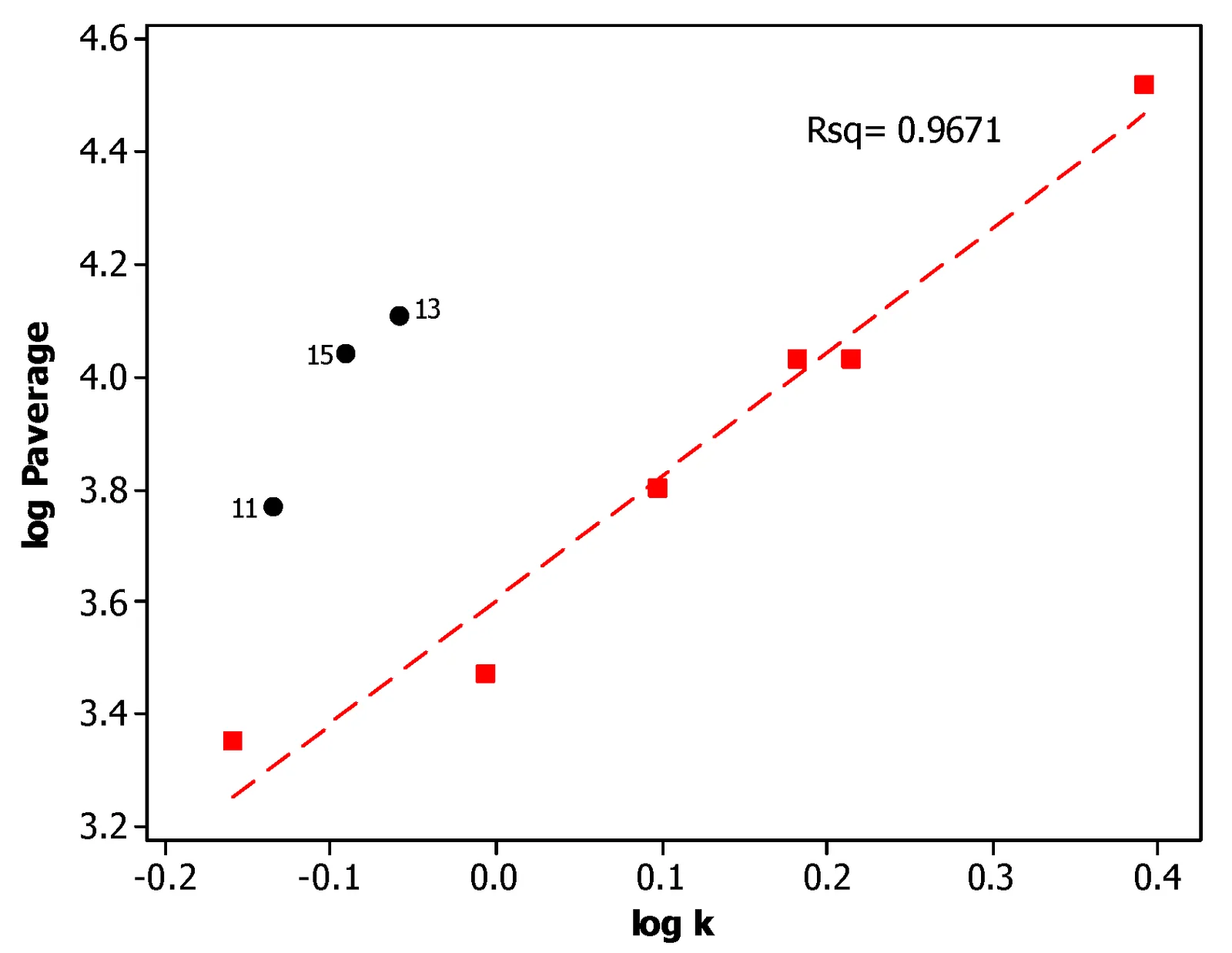
The relationship between computed log *P*_average_ values and experimentally determined log *k* values. This correlation was established for six fluorophenylated fused 1,2,4-triazinones (**10**, **12**, **14** and **16**–**18**). Log *P*_average_—the logarithmic *n*-octanol-water partition coefficient computed in silico as an average value from different computational methods for estimating log *P* (Alog P_s_, AClog P, Alog P, Mlog P, xlog P2 and xlog P3); log *k*—the logarithmic retention factor obtained by RP-HPLC.

For the six tested molecules (**10**, **12**, **14**, **16**, **17** and **18**), a statistically significant and linear relationship was established between the computed log *P*_average_ values and the experimentally determined log *k* values (Figure 9). This dependence was expressed by the following equation:

log *P*_average_ = 3.602(0.043) + 2.203(0.203) log *k* s = 0.0864; R^2^ = 0.9671; R^2^_adj_ = 0.9589; F = 118; p = 0.0004, (1)

where s, R^2^, R^2^_adj_, F, and p denote the standard deviation, the coefficient of determination, the adjusted coefficient of determination, F-value from a statistical F-test, and the probability value, respectively.

However, attempts to correlate the computed log *P*_average_ values with experimental lipophilicity indices were unsuccessful for three compounds (**11**, **13** and **15**) containing substituents at the *ortho* position of the phenyl ring. These derivatives behave as typical outliers, giving scattered data points shown in Figure 9, most likely due to the steric effect in their molecules. In these organic substances, *ortho*-substituents—both electron-donating and electron-withdrawing—decrease their lipophilicity indices due to the *ortho* effect. This suggests that it is not possible to correctly predict in silico the logarithmic *n*-octanol/water partition coefficients for these neutral small molecules. A weakness of the available computer software [80] was that theoretical methods for estimating log *P* (Alog P_s_, AClog P, Alog P, Mlog P, xlog P2 and xlog P3) were unable to distinguish between different positional isomers of the investigated compounds, for which the computed log *P*_average_ values (Table 8) were found to be the same or very close. Therefore, in this case, only the experimentally determined lipophilicity indices of these *ortho*-substituted molecules (**11**, **13** and **15**) should be considered as helpful descriptors for modelling their pharmacokinetics and ADMETox profile in the early stages of the drug development process.

### 2.11. Logarithmic Human Serum Albumin–Water Partition Coefficients Versus Lipophilicity Indices of Fluorophenylated Fused 1,2,4-Triazinones (***10**–**18***)

**Figure 10:**
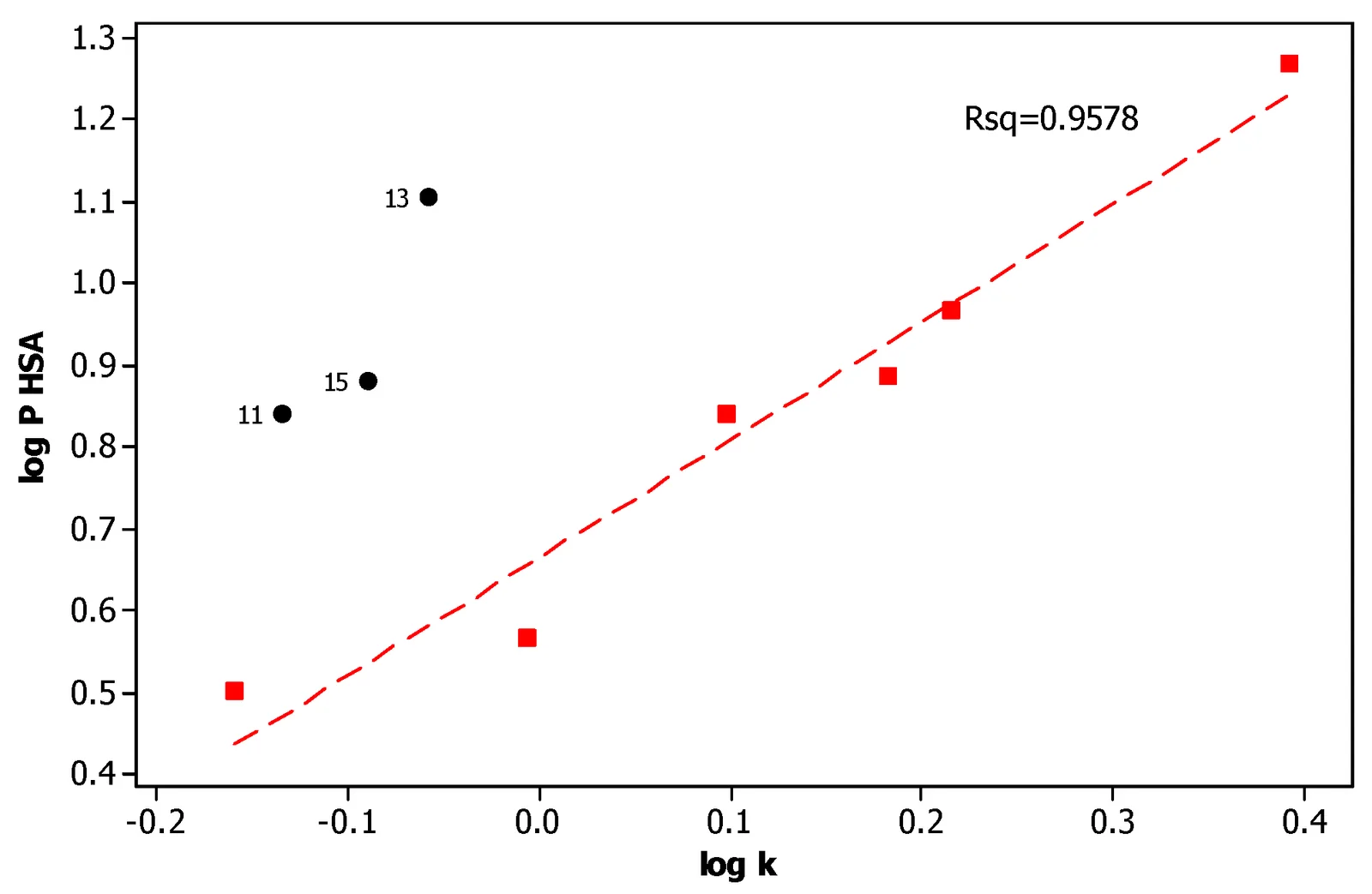
A relationship between the in silico predicted log *P*_HSA_ values and the experimentally determined log *k* values. This correlation was established for six fluorophenylated fused 1,2,4-triazinones (**10**, **12**, **14**, and **16**–**18**). Log *P*_HSA_—the logarithmic partition coefficient of a particular solute between human serum albumin and water; log *k*—the logarithmic retention factor obtained by RP-HPLC.

It is known that lipophilic/hydrophobic behaviour of drugs (during dynamic chromatographic separation processes mimicking biological processes) influences their binding to human serum albumin (HSA) and, consequently, their ADMETox profile. Binding to HSA is known to affect the distribution of potential molecular pharmaceutics within the organism, the rate of their metabolism, clearance, biological half-life and sometimes interactions between certain pharmaceutical substances. Chromatographic retention parameters are commonly used in in silico correlation studies with binding to human proteins [79,85,86,87], including human serum albumin as the major plasma protein, accounting for 60% of all plasma proteins [82]. The log *P*_HSA_ value describes the partitioning of the molecule between human serum albumin and water, which controls the concentration of active drug fraction in various tissues and organs in equilibrium with plasma [79]. Fluorophenylated fused *as*-triazinones (**10**–**18**) are predicted to bind to human proteins (mainly to HSA, as in the case of the vast majority of neutral pharmaceutics [79]), and their affinity for HSA may significantly affect their distribution [82]. Since the lipophilicity of drug candidates affects their binding to human proteins and consequently their distribution to various tissues and organs, it was worthwhile to correlate the estimated in silico log *P*_HSA_ descriptors of the title small molecules with their experimentally determined lipophilicity indices (logs *k*) (Table 8). This was especially important because all compounds would in the future be subjected to further more advanced procedures in the process of research and development of anticancer drugs. However, three *ortho*-substituted solutes (**11**, **13** and **15**) had to be excluded from the regression equation as evident outliers due to their lower retention in RP-HPLC measurements resulting from the steric effect mentioned above [84]. Finally, a strong, statistically significant and linear relationship was obtained (Figure 10) between the predictors of log *P*_HSA_ and lipophilicity indices (logs *k*) for the six tested compounds (**10**, **12**, **14**, and **16**–**18**). This dependence was expressed by the following equation:

log *P*_HSA_ = 0.664(0.032) + 1.440(0.151) log *k* s = 0.0643; R^2^ = 0.9578; R^2^_adj_ = 0.9472; F = 91; p = 0.0007, (2)

where s, R^2^, R^2^_adj_, F and p denote the standard deviation, the coefficient of determination, the adjusted coefficient of determination, F-value from a statistical F-test, and the probability value, respectively. The linear shape of this correlation suggests that lipophilicity is a significant physicochemical parameter. As retention factors increase, the binding equilibrium of tested molecules to human serum albumin/water also increases.

### 2.12. Principal Component Analysis

**Figure 11:**
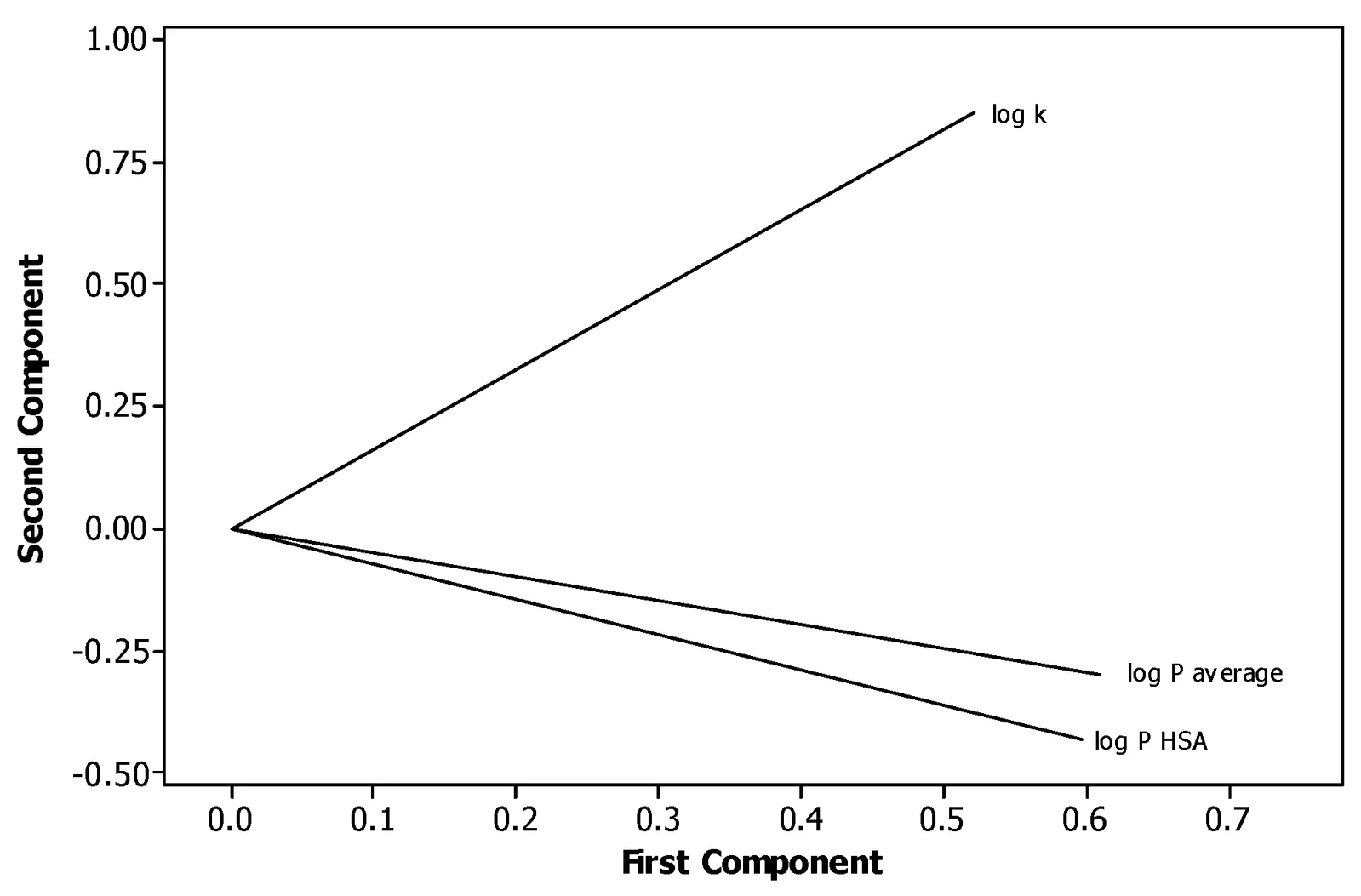
Principal component analysis (performed using Minitab 16 statistical software): the loading plot. Log *k*—the logarithmic retention factor obtained by RP-HPLC; log *P*_average_—the logarithmic *n*-octanol/water partition coefficient computed in silico as an average value from different computational methods for estimating log P (Alog Ps, AClog P, Alog P, Mlog P, xlog P2 and xlog P3); log *P*_HSA_—the logarithmic partition coefficient of a particular solute between human serum albumin and water.

**Figure 12:**
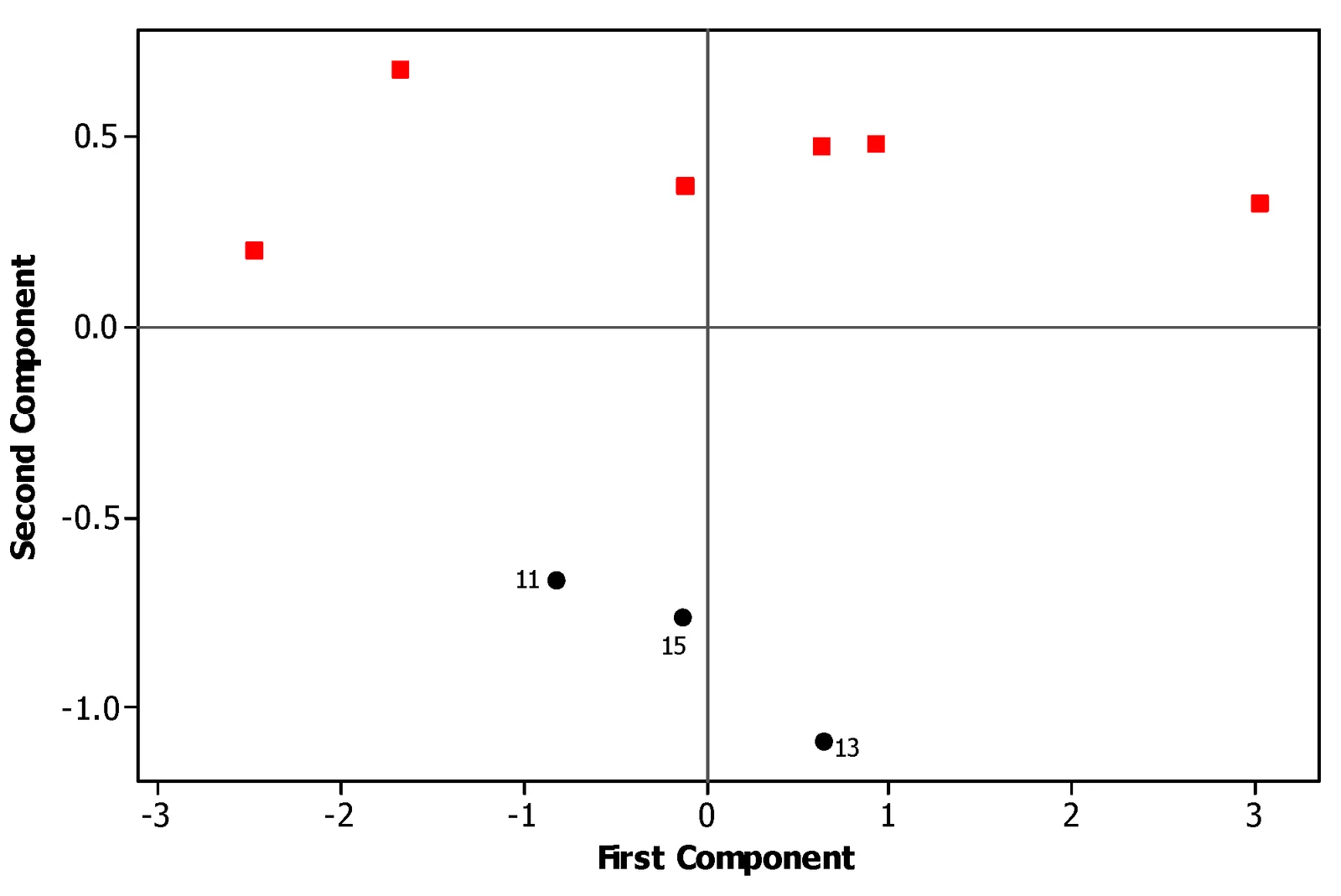
Principal component analysis (performed using Minitab 16 statistical software): the score plot.

In the present study, principal component analysis (PCA) was used as a helpful data processing method; it allows transforming all variables into linear combinations called principal components (PCs) [88,89]. PCA results were obtained using the following variables: logarithmic retention factors (logs *k*) obtained by RP-HPLC, computationally derived partitioning coefficients (logs *P*_average_) and pharmacokinetic-related human serum albumin–water partition coefficients (logs *P*_HSA_) of fluorophenylated fused *as*-triazinones (**10**–**18**). When plots of the first two principal components were considered, the first component (FC) explained 84.9%, while the second component (SC) explained 99.2% of the total variance in the data. All variables of the first principal component were in the range from 0.521 to 0.610, while all variables of the second principal component ranged from −0.434 to 0.849. Comparison of the distances on the loading plot (Figure 11) between the first and second components led to the conclusion that there is a directly proportional correlation between the RP-HPLC-derived log *k*, computational log *P*_average_ and log *P*_HSA_ descriptors. Moreover, there is a strong correlation between the log *P*_average_ and log *P*_HSA_ descriptors. In turn, on the score plot (Figure 12) there are visible similarities as well as dissimilarities between all investigated molecules according to variables that characterise them (logs *k*, logs *P*_average_ and logs *P*_HSA_). It was observed that the tested compounds formed two distinctive clusters. The first cluster constitutes the first six compounds (**10**, **12**, **14**, **16**, **17** and **18**) with a positive value of the second component, and the second cluster represents the remaining three derivatives (**11**, **13** and **15**) with a negative value of the second component. It can be seen that the grouping of the tested molecules is clearly dependent on the substituent attached to the phenyl ring. The substitution pattern appeared to be the most important structural factor influencing the chromatographic behaviour both experimentally determined and computed in silico as well as the modelled pharmacokinetic property of fluorophenylated fused *as*-triazinones.

## 3. Materials and Methods

### 3.1. Chemistry

#### 3.1.1. General Materials and Instrumentation

2-(4-Fluorophenyl)-2-oxoacetic acid stored under nitrogen and anhydrous triethylamine—of the highest grade available—were purchased from Combi-Blocks (San Diego, CA, USA) and Merck (Darmstadt, Germany), respectively. These reagents were used without further purification, with the proviso that triethylamine had to be stored over potassium hydroxide (Sigma-Aldrich, Saint Louis, MO, USA) prior to use. The organic solvents for synthesis (i.e., methanol—MeOH and dimethylformamide—DMF) from Roth GmhH (Karlsruhe, Germany) were employed. All novel compounds (**10**–**18**) were recrystallised, dried using the dry heat steriliser MOV-212S-PE purchased from Panasonic Healthcare Co. Ltd. (Ora-Gun, Gunma, Japan), and then characterised based on their elemental analyses, melting point determinations, and spectroscopic data (ATR-IR, ^1^H NMR, ^13^C NMR). Thin-layer chromatography (TLC)—employing commercially available SiO_2_ 60 F_254_ aluminium sheets (Merck, Darmstadt, Germany)—was used to follow up the heterocyclisation reaction and establish the homogeneity of each target compound sample, which was then visualised under a UV lamp (Herolab, Wiesloch, Germany) giving an intense blue fluorescence. A Boetius micro melting point apparatus (Franz Kustner Nachf. KG, Dresden, Germany) was employed to determine the melting point (m.p.), which is presented without any corrections for each compound sample. The elemental composition of new compounds was established by combustion microanalyses of C, H, and N, which were agreeable within ±0.4% of the theoretical values. A Thermo Scientific FTIR Nicolet 8700 spectrometer (Madison, WI, USA) (with a diamond crystal within the range of 4000–400 cm^−1^ and the spectral resolution of 4 cm^−1^) was used to record the attenuated total reflectance-infrared (ATR-IR) spectrum at ambient temperature of each compound. A Bruker Avance spectrometer (Bruker BioSpin GmbH, Karlsruhe, Germany) was employed to record the ^1^H NMR and ^13^C NMR spectra of each compound sample at ambient temperature. ^1^H NMR and ^13^C NMR spectra were measured at 500 and 125 MHz, respectively, in the reference solvent—deuterated dimethyl sulfoxide (DMSO-*d_6_*, Merck, Darmstadt, Germany). The chemical shift values are reported in ppm in reference to tetramethylsilane (TMS, Sigma-Aldrich, Munich, Germany) as a standard (δ = 0 ppm). The four-letter abbreviations for the signal multiplicity are as follows: s—singlet, d—doublet, pt—pseudotriplet, and m—multiplet. The presented coupling constant (*J*) values are expressed in hertz (Hz). The retention times (*t*_R_) of compounds were measured on the ODS-2 HPLC column: Spherisorb; 125 × 4 mm i.d., 5 μm (Merck, Darmstadt, Germany) at 298K, employing as the mobile phase a filtered and degassed mixture of water and acetonitrile (Merck, Darmstadt, Germany)—H_2_O/CH_3_CN (4:6, *v*/*v*).

#### 3.1.2. Synthesis of 1-Aryl-2-hydrazinylideneimidazolidine Hydroiodides (**1**–**9**)

Pharmacologically important starting materials of 1-aryl-2-hydrazinylideneimidazolidine hydroiodides (**1**–**9**)—which were not commercially available—were resynthesised in a multi-gram scale for the current research needs, applying our general synthetic procedure patented previously [14]. Prior to use, these crude heterocyclic hydrazones were recrystallised from propan-2-ol to obtain highly pure compounds **1**–**9**.

#### 3.1.3. A General Procedure for Synthesis of 8-(R-Phenyl)-3-(4-fluorophenyl)-7,8-dihydroimidazo[2,1-*c*][1,2,4]triazin-4(*6H*)-ones (**10**–**18**)

1-(R-phenyl)-2-hydrazonoimidazolidine hydroiodide (0.02 mole) was dissolved/suspended in 10 mL of methanol containing a molar excess of triethylamine (2.8 mL). Next, 2-(4-fluorophenyl)-2-oxoacetic acid (0.02 mole) was added. Just then, there occurred an exothermic effect. The reaction mixture was kept at room temperature as long as the intermediate precipitate had appeared. An appropriate amount of DMF was then added, and the cyclocondensation process was carried out until the intermediate product precipitate was completely dissolved during boiling of the reaction mixture and a one-phase system was obtained. The resultant liquid reaction solution was still refluxed for 4–5 h. The progress of heterocyclisation reaction was monitored by TLC, and the ultraviolet lamp at 254 nm was used to achieve visualisation of the spots of the starting materials and the final reaction products. The reaction solution was left to stand overnight. It was then concentrated to ca. half its volume by evaporation under reduced pressure. The residue was cooled to room temperature and remained in a refrigerator to give a crude solid product. This product was filtered off, washed three times with 10 mL of hot water, then three times with 10 mL of cold methanol, and allowed to air-dry. Each final product (**10**–**18**) crystallised from DMF/MeOH or DMF, and upon cooling the solution to room temperature, a highly pure solid was formed, which was filtered off and finally dried. There was no need for its further recrystallisation because its melting point did not change.

##### 3-(4-Fluorophenyl)-8-phenyl-7,8-dihydroimidazo[2,1-*c*][1,2,4]triazin-4(*6H*)-one (**10**)

Recrystallised from DMF/MeOH (3:1) mixture; creamy solid, m.p. 247–248 °C; yield 75.5%. Anal. Calcd for C_17_H_13_FN_4_O (308.31): C, 66.23%; H, 4.25%; N, 18.17%; Found: C, 66.01%; H, 4.24%; N, 18.24%. IR (ATR-IR) (ν, cm^−1^): 3079, 3047 (aromatic C–H stretching), 1670 (C=O stretching), 1601, 1585, 1495, 1455 (aromatic skeleton stretching), 1554 (C=N stretching), 1316 (C–F stretching, monofluorinated benzene ring), 851–815 (1,4-disubstituted benzene ring), 749–686 (monosubstituted benzene ring); ^1^H NMR (δ, ppm, DMSO-*d_6_*, TMS, 500 MHz): 4.22 (s, 4H, 2CH_2_), 7.16–8.21 (m, 9H, aromatic–H); ^13^C NMR (δ, ppm, DMSO-*d_6_*, TMS, 125 MHz): 164.2, 162.2 (d, ^1^*J_C–F_* = 246.7 Hz, C4′), 152.2 (C4), 152.1 (C8a), 147.1 (C3), 139.2, 130.5, 130.4 (d, ^3^*J_C–F_* = 8.3 Hz, C2′ and C6′), 129.4, 124.0, 119.2, 115.6, 115.4 (d, ^2^*J_C–F_* = 21.4 Hz, C3′ and C5′), 45.6 (C7), 40.9 (C6); HPLC _ODS-2 (298K)_ H_2_O/MeCN (4:6, *v*/*v*): *t*_R_ = 104.3 s.

##### 3-(4-Fluorophenyl)-8-(2-methylphenyl)-7,8-dihydroimidazo[2,1-*c*][1,2,4]triazin-4(*6H*)-one (**11**)

Recrystallised from DMF/MeOH (3:2) mixture; white solid; m.p. 255–257 °C; yield 77.5%. Anal. Calcd for C_18_H_15_FN_4_O (322.34): C, 67.07%; H, 4.69%; N, 17.38%; Found: C, 66.85%; H, 4.67%; N, 17.31%. IR (ATR-IR) (ν, cm^−1^): 3064, 3017 (aromatic C–H stretching), 1668 (C=O stretching), 2963, 2899 (methyl-CH, methylene-CH), 1597, 1504, 1464 (aromatic skeleton stretching), 1555 (C=N stretching), 1464, 1376 (aromatic C-CH_3_), 1316 (C–F stretching, monofluorinated benzene ring), 855–825 (1,4-disubstituted benzene ring), 762 (1,2-disubstituted benzene ring); ^1^H NMR (δ, ppm, DMSO-*d_6_*, TMS, 500 MHz): 2.30 (s, 3H, CH_3_), 4.30 (pt, *J* = 8.8 Hz, 2H, CH_2_), 4.07 (pt, *J* = 8.8 Hz, 2H, CH_2_), 7.26–8.13 (m, 8H, aromatic–H); ^13^C NMR (δ, ppm, DMSO-*d_6_*, TMS, 125 MHz): 164.0, 162.0 (d, ^1^*J_C–F_* = 246.2 Hz, C4′), 153.3 (C4), 152.5 (C8a), 146.2 (C3), 137.0, 136.6, 131.5, 130.8, 130.8 (d, ^3^*J_C–F_* = 8.3 Hz, C2′ and C6′), 130.3, 130.3, 128.6, 127.6, 127.4, 115.5, 115.3 (d, ^2^*J_C–F_* = 21.5 Hz, C3′ and C5′), 48.3 (C7), 42.0 (C6), 18.0 (CH_3_); HPLC _ODS-2 (298K)_ H_2_O/MeCN (4:6, *v*/*v*): *t*_R_ = 91.0 s.

##### 3-(4-Fluorophenyl)-8-(4-methylphenyl)-7,8-dihydroimidazo[2,1-*c*][1,2,4]triazin-4(*6H*)-one (**12**)

Recrystallised from DMF; yellow solid; m.p. 271–273 °C; yield 79.9%. Anal. Calcd for C_18_H_15_FN_4_O (322.34): C, 67.07%; H, 4.69%; N, 17.38%; Found: C, 67.29%; H, 4.71%; N, 17.44%. IR (ATR-IR) (ν, cm^−1^): 3114, 3084, 3044 (aromatic C–H stretching), 2973, 2921 (methyl-CH, methylene-CH), 1677 (C=O stretching), 1581, 1500 (aromatic skeleton stretching), 1542 (C=N), 1470, 1375 (aromatic C-CH_3_), 1309 (C–F stretching, monofluorinated benzene ring), 835 (1,4-disubstituted benzene ring), 799 (1,4-disubstituted benzene ring); ^1^H NMR (δ, ppm, DMSO-*d_6_*, TMS, 500 MHz): 2.32 (s, 3H, CH_3_), 4.20 (s, 4H, 2CH_2_), 7.26–8.21 (m, 8H, aromatic–H); ^13^C NMR (δ, ppm, DMSO-*d_6_*, TMS, 125 MHz): 164.1, 162.2 (d, ^1^*J_C–F_* = 247.0 Hz, C4′), 152.3 (C4), 152.1 (C8a), 146.8 (C3), 136.8, 133.2, 130.6, 130.6, 130.5, 130.4 (d, ^3^*J_C–F_* = 8.5 Hz, C2′ and C6′), 129.8, 119.2, 115.6, 115.4 (d, ^2^*J_C–F_* = 21.5 Hz, C3′ and C5′), 45.7 (C7), 40.9 (C6), 20.8 (CH_3_); HPLC _ODS-2 (298K)_ H_2_O/MeCN (4:6, *v*/*v*): *t*_R_ = 118.3 s.

##### 8-(2,3-Dimethylphenyl)-3-(4-fluorophenyl)-7,8-dihydroimidazo[2,1-*c*][1,2,4]triazin-4(*6H*)-one (**13**)

Recrystallised from DMF/MeOH (1:1) mixture; white solid; m.p. 219–221 °C; yield 62.4%. Anal. Calcd for C_19_H_17_FN_4_O (336.36): C, 67.84%; H, 5.09%; N, 16.66%; Found: C, 67.96%; H, 5.11%; N, 16.60%. IR (ATR-IR) (ν, cm^−1^): 3064, 3007 (aromatic C–H stretching), 2983, 2893 (methyl-CH, methylene-CH), 1669 (C=O stretching), 1597, 1503 (aromatic skeleton stretching), 1555 (C=N stretching), 1474, 1373 (aromatic C-CH_3_), 1313 (C–F stretching, monofluorinated benzene ring), 855–812 (1,4-disubstituted benzene ring), 779–677 (1,2,3-trisubstituted benzene ring); ^1^H NMR (δ, ppm, DMSO-*d_6_*, TMS, 500 MHz): 2.18 (s, 3H, CH_3_), 2.32 (s, 3H, CH_3_), 4.30 (pt, *J* = 8.8 Hz, 2H, CH_2_), 4.03 (pt, *J* = 8.8 Hz, 2H, CH_2_), 7.21–8.13 (m, 7H, aromatic–H); ^13^C NMR (δ, ppm, DMSO-*d_6_*, TMS, 125 MHz): 163.9, 162.0 (d, ^1^*J_C–F_* = 246.3 Hz, C4′), 153.6 (C4), 152.5 (C8a), 146.1 (C3), 138.5, 137.0, 135.3, 130.9, 130.8, 130.3, 130.2 (d, ^3^*J_C–F_* = 8.2 Hz, C2′ and C6′), 129.9, 126.7, 125.1, 115.5, 115.3 (d, ^2^*J_C–F_* = 21.2 Hz, C3′ and C5′), 48.6 (C7), 42.0 (C6), 20.4 (CH_3_), 14.6 (CH_3_); HPLC _ODS-2 (298K)_ H_2_O/MeCN (4:6, *v*/*v*): *t*_R_ = 98.7 s.

##### 3-(4-Fluorophenyl)-8-(2-methoxyphenyl)-7,8-dihydroimidazo[2,1-*c*][1,2,4]triazin-4(*6H*)-one (**14**)

Recrystallised from DMF/MeOH (3:2) mixture; creamy solid; m.p. 201–203 °C; yield 72.8%. Anal. Calcd for C_18_H_15_FN_4_O_2_ (338.33): C, 63.90%; H, 4.47%; N, 16.56%; Found: C, 64.12%; H, 4.45%; N, 16.50%. IR (ATR-IR) (ν, cm^−1^): 3080, 3055 (aromatic C–H stretching), 2975, 2946, 2839 (methyl-CH, methylene-CH), 1681 (C=O stretching), 1599, 1502 (aromatic skeleton stretching), 1550 (C=N stretching), 1487–1436 (methylene-CH), 1314 (C–F stretching, monofluorinated benzene ring), 1229, 1014 (C–O–C of arylalkyl ether), 835–812 (1,4-disubstituted benzene ring), 765 (1,2-disubstituted benzene ring); ^1^H NMR (δ, ppm, DMSO-*d_6_*, TMS, 500 MHz): 3.85 (s, 3H, OCH_3_), 4.28 (pt, *J* = 8.9 Hz, 2H, CH_2_), 4.05 (pt, *J* = 8.9 Hz, 2H, CH_2_), 7.06–8.14 (m, 8H, aromatic–H); ^13^C NMR (δ, ppm, DMSO-*d_6_*, TMS, 125 MHz): 164.0, 162.0 (d, ^1^*J_C–F_* = 246.2 Hz, C4′), 155.4, 153.7 (C4), 152.4 (C8a), 146.3 (C3), 130.8, 130.8, 130.3, 130.3 (d, ^3^*J_C–F_* = 8.2 Hz, C2′ and C6′), 129.6, 129.0, 126.4, 121.1, 115.5, 115.3 (d, ^2^*J_C–F_* = 21.2 Hz, C3′ and C5′), 113.1, 56.2 (CH_3_O), 47.6 (C7), 41.9 (C6); HPLC _ODS-2 (298K)_ H_2_O/MeCN (4:6, *v*/*v*): *t*_R_ = 88.9 s.

##### 8-(2-Chlorophenyl)-3-(4-fluorophenyl)-7,8-dihydroimidazo[2,1-*c*][1,2,4]triazin-4(*6H*)-one (**15**)

Recrystallised from DMF/MeOH (3:1) mixture; white solid; m.p. 264–266 °C; yield 70.1%. Anal. Calcd for C_17_H_12_ClFN_4_O (342.75): C, 59.57%; H, 3.53%; N, 16.35%; Found: C, 59.79%; H, 3.50%; N, 16.29%. IR (ATR-IR) (ν, cm^−1^): 3066, 2990 (aromatic C–H stretching), 1672 (C=O stretching), 1597, 1506, 1448 (aromatic skeleton stretching), 1556 (C=N stretching), 1320 (C–F stretching, monofluorinated benzene ring), 1098 (aromatic C–Cl stretching), 849–811 (1,4-disubstituted benzene ring), 784–754 (1,2-disubstituted benzene ring); ^1^H NMR (δ, ppm, DMSO-*d_6_*, TMS, 500 MHz): 4.33 (pt, *J* = 8.8 Hz, 2H, CH_2_), 4.10 (pt, *J* = 8.8 Hz, 2H, CH_2_), 7.27–8.13 (m, 8H, aromatic–H); ^13^C NMR (δ, ppm, DMSO-*d_6_*, TMS, 125 MHz): 164.1, 162.1 (d, ^1^*J_C–F_* = 246.4 Hz, C4′), 153.6 (C4), 152.3 (C8a), 146.9 (C3), 135.7, 132.1, 130.8, 130.6, 130.6 (d, ^3^*J_C–F_* = 8.3 Hz, C2′ and C6′), 130.5, 130.4, 130.4, 128.9, 115.6, 115.4 (d, ^2^*J_C–F_* = 21.6 Hz, C3′ and C5′), 48.0 (C7), 42.2 (C6); HPLC _ODS-2 (298K)_ H_2_O/MeCN (4:6, *v*/*v*): *t*_R_ = 95.2 s.

##### 8-(3-Chlorophenyl)-3-(4-fluorophenyl)-7,8-dihydroimidazo[2,1-*c*][1,2,4]triazin-4(*6H*)-one (**16**)

Recrystallised from DMF/MeOH (3:1) mixture; creamy solid; m.p. 234–236 °C; yield 72.2%. Anal. Calcd for C_17_H_12_ClFN_4_O (342.75): C, 59.57%; H, 3.53%; N, 16.35%; Found: C, 59.48%; H, 3.55%; N, 16.41%. IR (ATR-IR) (ν, cm^−1^): 3110, 3080, 2998 (aromatic C–H stretching), 1664 (C=O stretching), 1598, 1484 (aromatic skeleton stretching), 1544 (C=N stretching), 1323 (C–F stretching, monofluorinated benzene ring), 1094 (aromatic C–Cl stretching), 841–812 (1,4-disubstituted benzene ring), 882–745 (1,3-disubstituted benzene ring); ^1^H NMR (δ, ppm, DMSO-*d_6_*, TMS, 500 MHz): 4.22 (s, 4H, 2CH_2_), 7.22–8.22 (m, 8H, aromatic–H); ^13^C NMR (δ, ppm, DMSO-*d_6_*, TMS, 125 MHz): 164.3, 162.3 (d, ^1^*J_C–F_* = 247.1 Hz, C4′), 152.1 (C4), 152.0 (C8a), 147.5 (C3), 140.6, 133.9, 131.0, 130.6, 130.6 (d, ^3^*J_C–F_* = 8.3 Hz, C2′ and C6′), 130.3, 130.3, 123.5, 118.7, 117.2, 115.6, 115.5 (d, ^2^*J_C–F_* = 21.6 Hz, C3′ and C5′), 45.6 (C7), 41.0 (C6); HPLC _ODS-2 (298K)_ H_2_O/MeCN (4:6, *v*/*v*): *t*_R_ = 138.6 s.

##### 8-(4-Chlorophenyl)-3-(4-fluorophenyl)-7,8-dihydroimidazo[2,1-*c*][1,2,4]triazin-4(*6H*)-one (**17**)

Recrystallised from DMF; creamy solid; m.p. 297–299 °C; yield 75.4%. Anal. Calcd for C_17_H_12_ClFN_4_O (342.75): C, 59.57%; H, 3.53%; N, 16.35%; Found: C, 59.66%; H, 3.50%; N, 16.30%. IR (ATR-IR) (ν, cm^−1^): 3111, 3084 (aromatic C–H stretching), 1680 (C=O stretching), 1579, 1492, 1480 (aromatic skeleton stretching), 1542 (C=N stretching), 1318 (C–F stretching, monofluorinated benzene ring), 1089 (aromatic C–Cl stretching), 845–798 (two 1,4-disubstituted benzene rings); ^1^H NMR (δ, ppm, DMSO-*d_6_*, TMS, 500 MHz): 4.22 (s, 4H, 2CH_2_), 7.29–8.21 (m, 8H, aromatic–H); ^13^C NMR (δ, ppm, DMSO-*d_6_*, TMS, 125 MHz): 164.2, 162.3 (d, ^1^*J_C–F_* = 247.2 Hz, C4′), 152.2 (C4), 152.1 (C8a), 147.3 (C3), 138.2, 130.6, 130.5 (d, ^3^*J_C–F_* = 8.2 Hz, C2′ and C6′), 130.4, 129.3, 127.7, 120.7, 115.6, 115.5 (d, ^2^*J_C–F_* = 21.6 Hz, C3′ and C5′), 45.6 (C7), 41.0 (C6); HPLC _ODS-2 (298K)_ H_2_O/MeCN (4:6, *v*/*v*): *t*_R_ = 132.3 s.

##### 8-(3,4-Dichlorophenyl)-3-(4-fluorophenyl)-7,8-dihydroimidazo[2,1-*c*][1,2,4]triazin-4(*6H*)-one (**18**)

Recrystallised from DMF; creamy solid, m.p. 299–300 °C; yield 72.9%. Anal. Calcd for C_17_H_11_Cl_2_FN_4_O (377.20): C, 54.13%; H, 2.94%; N, 14.85%; Found: C, 54.29%; H, 2.95%; N, 14.80%. IR (ATR-IR) (ν, cm^−1^): 3108, 3076, 3059, 3000 (aromatic C–H stretching), 1680 (C=O stretching), 1595, 1495, 1472 (aromatic skeleton stretching), 1564 (C=N stretching), 1313 (C–F stretching, monofluorinated benzene ring), 1094 (aromatic C–Cl stretching), 848–822 (1,4-disubstituted benzene ring), 885–689 (1,2,4-trisubstituted benzene ring); ^1^H NMR (δ, ppm, DMSO-*d_6_*, TMS, 500 MHz): 4.23 (s, 4H, 2CH_2_), 7.30–8.37 (m, 7H, aromatic–H); ^13^C NMR (δ, ppm, DMSO-*d_6_*, TMS, 125 MHz): 164.4, 162.3 (d, ^1^*J_C–F_* = 247.3 Hz, C4′), 152.1 (C4), 152.0 (C8a), 147.8 (C3), 139.3, 131.4, 131.2, 130.7, 130.6 (d, ^3^*J_C–F_* = 8.3 Hz, C2′ and C6′), 130.3, 125.4, 120.4, 118.9, 115.7, 115.5 (d, ^2^*J_C–F_* = 21.6 Hz, C3′ and C5′), 45.6 (C7), 41.0 (C6); HPLC _ODS-2 (298K)_ H_2_O/MeCN (4:6, *v*/*v*): *t*_R_ = 182.0 s.

### 3.2. In Vitro Studies

#### 3.2.1. Determination of the Cytotoxicity of Compounds in the DNA Synthesis and Cellular Proliferation Assay

5-Bromo-2′-deoxyuridine (BrdU)-based enzyme-linked immunosorbent assay [90,91,92] was conducted in order to study the in vitro antiproliferative activity of compounds against three tumour cell lines of epithelial origin (A549 (ECACC 86012804)—human Caucasian lung carcinoma cell line; HeLa (ECACC 93021013)—human Negroid cervix epithelioid carcinoma cell line; T47D (ECACC 85102201)—human breast carcinoma cell line) and their cytotoxicity against normal epithelial cells (GMK (ECACC 88020401)—African green monkey kidney cells). The A549, HeLa, T47D and GMK cell lines were derived from the European Collection of Authenticated Cell Cultures (ECACC). A commercially available 5-Bromo-2′-deoxyuridine Labeling/Detection Kit III (Roche Diagnostics GmbH, Mannheim, Germany)—based on the known principle that BrdU, as a labelled thymidine precursor, can be incorporated instead of thymidine into the DNA of exclusively proliferating cells—was employed for the studies according to the manufacturer’s protocol. The detailed experimental procedure that allowed the assessment of cell proliferation after exposure to molecules, as well as in untreated controls, has been reported earlier [8].

Briefly, each recruited cell line—maintained in RPMI-1640 medium (Sigma-Aldrich, Saint Louis, MO, USA) containing 10% foetal bovine serum (PBS, CytoGen, Greven, Germany) and penicillin-streptomycin (100 U mL^−1^, 100 μg mL^−1^, respectively) solution (Sigma-Aldrich, Saint Louis, MO, USA), routinely passaged twice a week—was pre-incubated on a 96-well microtiter plate (Nunc, Roskilde, Denmark). A freshly prepared stock solution of each test compound in dimethyl sulphoxide (DMSO, Roth GmhH, Karlsruhe, Germany) was filtered through a sterile non-pyrogenic syringe filter (0.22 μm-pore-size, GE Water and Process Technologies, Trevose, PA, USA) and diluted in cell culture medium to obtain the appropriate concentration. Cell cultures treated with compound solutions or untreated controls were incubated under standard conditions (37 °C, 5% CO_2_, 90% humidity) for 24, 48 and 72 h. Pemetrexed (Eli Lilly, Bratislava, Slovakia) and gemcitabine (Apollo Scientific Ltd., Stockport, UK) were used as reference anticancer agents. Endpoint determinations were performed using the colorimetric BrdUrd assay on an ELISA reader (BIO-TEK Instruments Inc., Winooski, VT, USA). The percentage of growth inhibition was calculated according to the following formula:

Growth inhibition (%) = 100 − [(Abs_sample_ − Abs_blank_)/(Abs_control_ − Abs_blank_)] × 100, (3)

where: Abs_sample_—the absorbance of sample with the tested compound, Abs_control_—the absorbance of sample without the tested compound, Abs_blank_—the absorbance of solvent. It should be added that the absorbance of the sample is directly correlated with the level of labelled thymidine precursor incorporated into cellular DNA during replication. Finally, the results were expressed as the mean percentage growth inhibition ± standard deviation.

For each compound tested, an IC_50_ value was calculated based on a dose/response curve for each cell line. The IC_50_ is the concentration of compound required to inhibit cell proliferation by 50% compared to untreated cells. In addition, a selectivity index was calculated for each compound as the ratio of its IC_50_ value in the normal cell line to its IC_50_ value in the tumour cell line.

The results taken from five independent experiments were averaged and then expressed as the mean ± standard deviation. Statistical analyses were performed using STATISTICA 12.5PL software (StatSoft, Cracow, Poland). The statistical significance level was defined as *p* < 0.05 (*) or *p* < 0.001 (**).

#### 3.2.2. Determination of the Cytotoxicity of Compounds by the Trypan Blue Exclusion Assay

In this experiment, human tumour cell lines: MM.1R (ATCC CRL2975)—multiple myeloma cell line (derived from the American Type Culture Collection—ATCC) as well as two haematological tumour cell lines, i.e., Jurkat E6-1 (ECACC 88042803)—acute T-lymphocytic leukaemia cell line and HL-60 (ECACC 98070106)—acute promyelocytic leukaemia cell line (derived from European Collection of Authenticated Cell Cultures—ECACC)—were incubated under standard conditions in a humidified atmosphere for 24, 48 and 72 h with individual compounds. After staining with trypan blue (Sigma-Aldrich, Saint Louis, MO, USA), all non-viable cells were visible as blue-stained cells, allowing direct counting of the remaining viable cells. In this assay, the viability of cells was assessed with an automated cell counter (Invitrogen, Carlsbad, CA, USA) using cell counting chamber slides (Invitrogen, Carlsbad, CA, USA). Gemcitabine (Apollo Scientific Ltd., Stockport, UK) was used as a reference anticancer agent. The results were converted to cytotoxicity expressed as the percentage of dead cells after exposure to each test compound. The IC_50_ values were also calculated.

The results taken from two independent experiments were averaged and then expressed as the mean ± standard deviation. Statistical analyses were carried out using STATISTICA 12.5PL software (StatSoft, Cracow, Poland). The statistical significance level was defined as *p* < 0.05 (*) or *p* < 0.001 (**).

#### 3.2.3. Measurement of the Effect of Compounds on Caspase Levels in Normal and Tumour Cells

Human Caspase-3, -7, -9 (CASP3, CASP7, CASP9) ELISA kits—purchased from SunRed Biotechnology Company, Ltd. (Shanghai, China) and based on the double-antibody sandwich enzyme-linked immunosorbent assay (DAS-ELISA)—were used in this experimental procedure. Employing a standard curve for a particular caspase, the levels of caspase-3 or caspase-7 or caspase-9 were determined quantitatively in both untreated (controls) and treated (with each compound at a concentration of 150 μM) cell lysates of normal (GMK) and tumour (A549, HeLa, T47D) cells of the same epithelial origin. All determinations, preparation of reagents and standards were done according to the manufacturer’s protocol, as previously described [11]. Briefly, each cell lysate was added to the micro ELISA plate, well pre-coated with the human caspase-3 or caspase-7 or caspase-9 monoclonal antibody. Next, a biotinylated detection antibody specific for caspase-3 or caspase-7 or caspase-9 and a streptavidin-horseradish peroxidase conjugate were added to the same microplate well and incubated at 37 °C for 60 min to form a blue-stained immune complex. To remove uncombined enzyme, each microplate well was washed five times with wash buffer. Next, after adding two substrate solutions (A and B), incubation was continued at 37 °C for 10 min in the darkness. Finally, acidic stop solution was added to terminate the enzyme–substrate reaction and the colour turned yellow. 

The results taken from three independent experiments were statistically analysed employing the STATISTICA 12.5PL software (StatSoft, Cracow, Poland). Statistically significant differences compared to the control group were observed for *p*-values less than 0.05 at the 95% confidence level. The obtained data were expressed as the mean ± standard deviation.

### 3.3. Ex Vivo Studies

#### 3.3.1. Red Blood Cells

Sheep blood purchased from BioMaxima S.A. (Lublin, Poland) was centrifuged (1000 rpm, 10 min, 4 °C) and, after plasma separation, red blood cells (RBCs) were washed three times with phosphate-buffered saline (PBS, pH 7.4, Biomed, Lublin, Poland). RBCs and PBS were mixed to obtain a 4% suspension, which was used to determine the haemolytic and antiheamolytic activity of the compounds.

#### 3.3.2. Haemolytic Activity Assay

The haemolytic activity of the compounds was assessed in an ex vivo model of erythrocytes according to the procedure described earlier [12]. In this assay, each test compound at a concentration of 150 μM was incubated (37 °C, 60 min) with a suspension of erythrocytes. The samples were then centrifuged (3000 rpm, 10 min) and the absorbance at λ_max_ = 540 nm of each supernatant was measured using a Hitachi U2800 spectrophotometer (Hitachi, Tokyo, Japan). The negative control (0% haemolysis of RBCs) was a suspension of erythrocytes in PBS, while the positive control (100% haemolysis of RBCs) was the suspension of erythrocytes in triton X-100 (polyoxyethylene octyl phenyl ether, Sigma-Aldrich, Saint Louis, MO, USA).

Data (from three independent experiments) were shown as the mean ± standard deviation. Statistical analyses were performed using STATISTICA 12.5PL software (StatSoft, Cracow, Poland). *p*-values < 0.05 were considered statistically significant.

#### 3.3.3. Oxidative Haemolysis Inhibition Assay

The antihaemolytic activity of compounds was assessed in an ex vivo model of erythrocytes according to the procedure described earlier [10]. Briefly, each test compound or antioxidant standard—ascorbic acid, trolox (Sigma-Aldrich, Saint Louis, MO, USA)—at a concentration of 150 μM was incubated (37 °C, 60 min) with the suspension of erythrocytes. A freshly prepared 40 mM AAPH (2,2′-azobis(2-amidinopropane dihydrochloride), Sigma-Aldrich, Saint Louis, MO, USA) or 75 mM H_2_O_2_ (hydrogen peroxide, Sigma-Aldrich, Saint Louis, MO, USA) ice-cold solution in PBS was then added to each sample, followed by incubation at 37 °C for 180 or 210 min, respectively. After centrifugation (1000 rpm, 5 min), the absorbances of samples containing AAPH were measured on a Hitachi U2800 spectrophotometer (Hitachi, Tokyo, Japan) at λ_max_ = 524 nm, and those containing H_2_O_2_ at λ_max_ = 540 nm. The antihaemolytic activity of compounds was determined in relation to antioxidant standards (ascorbic acid, trolox).

Data (from four independent experiments) are shown as the mean ± standard deviation. Statistical analyses were carried out using STATISTICA 12.5PL software (StatSoft, Cracow, Poland). These values were considered statistically significant only for *p* less than 0.05.

### 3.4. In Silico Studies

#### 3.4.1. Molecular Docking

Molecular docking studies were performed to investigate the binding mode and thermodynamic affinity of fluorophenylated fused triazinones **10**–**18** within the binding pocket of adenosine A_2A_ and A_2B_ receptors. Docking workflows were executed on the Tamarind Bio platform (https://www.tamarind.bio/; accessed on 22 August 2026). The three-dimensional crystal structures of the adenosine A_2A_ and A_2B_ receptors were downloaded from the RCSB Protein Data Bank (PDB ID: 3EML and PDB ID: 8HDO, respectively) (https://www.rcsb.org; accessed on 22 August 2026) [37,38]. Prior to molecular docking, each receptor preparation was conducted via the automated pre-processing pipeline implemented in Tamarind Bio. This process included the removal of co-crystallised solvent molecules, ions, and the reference ligand (ZM-241385 from A_2A_ AR or BAY 60-6583 from A_2B_ AR), followed by the addition of polar hydrogen atoms, restoration of missing side chains, and calculation of partial atomic charges under standard physiological pH conditions.

To validate the docking workflow and serve as a reference for binding affinity comparison, ZM-241385 and PSB-1115 selective antagonists of A_2A_ and A_2B_ AR, respectively, were docked under identical parameters. Their 3D structures were obtained from the PubChem database in smiles format.

Ligand preparation, including geometry optimisation, minimisation of steric clashes, and assignment of appropriate ionisation states, was fully handled by the Tamarind Bio input validation module prior to simulation.

Docking simulations were driven by the AutoDock Vina [39,40] engine integrated into the Tamarind Bio platform. The grid box was centred on the orthosteric binding site of each antagonist, precisely encapsulating the coordinates of the native co-crystallised ligand. The grid centre parameters were set to X = −10.376, Y = −13.784, and Z = 57.487 (for A_2A_ AR) or X = 138.276, Y = 127.761, and Z = 106.258 (for A_2B_ AR), with an optimised search box volume of 47 × 37 × 51 Å (for A2_A_ AR) or 22.5 × 22.5 × 22.5 Å (for A2_B_ AR) to provide sufficient conformational freedom for the ligand’s rotatable bonds. The exhaustiveness parameter was set to 8 or 32, respectively, to ensure that the binding site was scanned over a wider range in all docking operations.

The nine binding poses were generated for each ligand and ranked according to the calculated binding affinity, expressed in kcal/mol. The conformations with the most favourable (lowest) values were retained as the most stable. Further analyses of non-covalent interaction network were performed and visualised using PLIP—Protein-Ligand Interaction Profiler (https://plip-tool.biotec.tu-dresden.de/plip-web/plip/index; accessed on 24 August 2026) [44].

#### 3.4.2. Toxicity Risk and ADME Prediction

The risk of adverse side effects of compounds was predicted by the OSIRIS Property Explorer tool (accessed on 27 January 2026) [55]. Descriptors assessing drug-likeness and parameters characterising the ADME processes of compounds were calculated using pkCSM software (accessed on 27 January 2026) [62]. In silico calculations were performed for compound structures saved in the SMILES format.

### 3.5. Lipophilicity Studies

#### 3.5.1. HPLC Equipment and Conditions for Determining Lipophilicity Indices

RP-HPLC measurements were conducted on a Shimadzu VP (Shimadzu Corporation, Columbia, MD, USA) apparatus. This liquid chromatograph was equipped with the LC 10AT pump, SPD 10A UV-Vis detector, SCL 10A system controller, and Rheodyne injector valve (IDEX Health & Science, Rohnert Park, CA, USA) with a 20 μL loop. Each chromatographic measurement was controlled by a CTO-10 AS thermostatic oven. Chromatograms were recorded at λ_max_ = 254 nm by a diode array UV absorption detector using a Shimadzu Class-VP software (version 6.1, Shimadzu Corporation, Kyoto, Japan). For analytical HPLC, in this case, the Spherisorb ODS-2 column, 125 × 4 mm i.d., 5 μm particle size (Merck, Darmstadt, Germany) was applied as the stationary phase, whereas the filtrated and degassed water/acetonitrile mixtures (40%/60% (*v*/*v*)) were used as effluents. The volume of mobile phase passing through the recruited column (i.e., flow rate) was 1 mL min^−1^. Nine fluorophenylated fused *as*-triazinones (**10**–**18**)—all of high purity—were used in this study to determine their retention factors, *k*. When each sample of the test compound was separately dissolved in acetonitrile—ca. 0.005 mg mL^−1^—and injected into the column, a symmetric peak was recorded. Sodium chloride (Sigma-Aldrich, Saint Louis, MO, USA) was used as non-retained compound to measure the dead time (*t*_0_). Each isocratic retention factor—which was obtained at a single mobile phase concentration—was calculated according to the following equation [93]:

log *k* = log [(*t*_R_ − *t*_0_)/*t*_0_], (4)

where *t*_R_ denotes the retention time of the investigated compound and *t*_0_ denotes the dead time value for the non-retained compound, which is =52.5 s.

Each log *k* value is the average of at least three independent measurements. All measurements were performed under isocratic conditions and at a constant temperature (25 °C). Acetonitrile—used as the organic modifier of the mobile phase—was of HPLC grade (Merck, Darmstadt, Germany). Deionised water was used as the inorganic component of the mobile phase. It was obtained from water by deionisation using a Direct-Q3 UV apparatus (Millipore, Darmstadt, Germany).

#### 3.5.2. In Silico Calculations and Statistical Analysis

Log *P*_HSA_ values, i.e., the logarithmic partition coefficients between human serum albumin and water, are pharmacokinetic descriptors that were calculated from molecular structures of the investigated compounds according to the Abraham’s equation based on linear solvation energy relationships (LSERs) [82,88], using ACD/Percepta software, version 1994-2012 (ACD/Labs, Advanced Chemistry Development, Inc., Toronto, ON, Canada). The logarithmic *n*-octanol/water partition coefficients were estimated as average values (logs *P*_average_) from six computational methods for estimating log *P* (i.e., Alog P_s_, AClog P, Alog P, Mlog P, xlog P2 and xlog P3). All computational *n*-octanol/water partition coefficients were estimated in silico using an online software package [80]. Statistical analysis as well as principal component analysis (PCA) were performed using Minitab 16 statistical software (Minitab Inc., State College, PA, USA).

## 4. Conclusions

A general synthetic procedure—based on a straightforward heteroannulation protocol—has been developed and optimised for all steps, which allowed us to obtain a new class of 7,8-dihydroimidazo[2,1-*c*][1,2,4]triazin-4(6*H*)-ones with the *para*-fluorophenyl moiety at *C*3 and nine different substituents at *N*8. The synthesis of the final products from the starting materials proceeded via an intermediate—ketimine—that was not isolated but cyclised in situ, which simplified the procedure. This innovative route was efficient, and required only a simple isolation (without the need for chromatographic separation) and crystallisation of the products in the final step. This minimised the necessary synthetic operations, directly reducing cost, time, and solvent consumption. These advantages would enable this method to be used for large-scale synthesis in a future production process. All new compounds (**10**–**18**) were designed on the basis of the concept of fluorine–hydrogen isosterism. The majority of them displayed a broad spectrum of anticancer activity, exhibiting potent antiproliferative effects against human lung, cervical, and breast carcinoma cells as well as human multiple myeloma and leukaemia (T-lymphocytic and promyelocytic) cells—with efficacy comparable or superior to the anticancer drugs, pemetrexed and gemcitabine. Consequently, fluorine–hydrogen isosteric replacement proved successful, broadening the spectrum of anticancer activity of fluorinated molecules compared to their non-fluorinated isosteres. The latter only decreased the viability of peripheral blood myeloma cells or inhibited cervical cancer cell motility. The finding that fluorinated dihydroimidazotriazinones **10**–**18** exhibit a broader spectrum of anticancer activity than their non-fluorinated analogues represents a significant contribution to the field of fluorine–hydrogen isosterism. Fluorophenylated molecules also demonstrated a favourable safety profile with high antihaemolytic activity (comparable or superior to that of antioxidant standards) in ex vivo erythrocyte models. Molecular docking revealed that the investigated compounds exhibited binding affinities superior or equal to those of the selective adenosine A_2A_ and A_2B_ receptor antagonists, ZM-241385 and PSB-1115, respectively. In addition, ligands **13**, **16**, and **17** were identified as promising dual-target candidates, possessing the structural features necessary to simultaneously interact with and block both adenosine receptor subtypes. This suggests that the molecules may exert their beneficial biological effects by acting as competitive antagonists, thereby preventing endogenous adenosine from binding and triggering its immunosuppressive signaling pathway. Furthermore, all the compounds under study were predicted to be drug-like with good ADME profiles. The log *k* indices for the vast majority of molecules (**10**, **12**, **14**, and **16**–**18**) strongly correlated with both their in silico log *P*_average_ and log *P*_HSA_ values. Structure–activity and structure–toxicity relationships demonstrated that the substituents at *N*8 of the privileged scaffold and the common *para*-fluorophenyl moiety at *C*3 are crucial for the pharmacological profiles of the compounds studied. Among the fluorophenylated fused *as*-triazinones, compounds **11**, **13**, **14**, **15**, and **17**—bearing the 2-methyl, 2,3-dimethyl, 2-methoxy, 2-chloro, and 4-chloro, respectively—were identified as the least toxic to normal cells. Therefore, these molecules—which exhibit a proven spectrum of antiproliferative and antihaemolytic activities alongside the ability to increase apoptotic caspase levels in human tumour cells—represent promising anticancer drug candidates that warrant further advanced investigation. They could serve as a basis for the rational design of less toxic, innovative anticancer agents. The presence of a very strong C–F bond (which is not broken during metabolic processes) in the phenyl moiety would result in enhanced metabolic stability of the compounds.

## Figures and Tables

**Table 1:** The IC_50_ and SI values for fluorophenylated fused 1,2,4-triazinones (**10**–**18**).

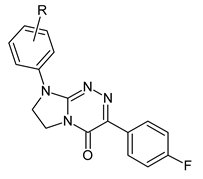												
Compound	R	Incubation Time	IC_50_ Values (µM)							SI		
			GMK	A549	HeLa	T47D	MM.1R	Jurkat E6-1	HL-60	A549	HeLa	T47D
**10**	H	24	150 ± 14 *	>150	25 ± 4 **	57 ± 4 **	150 ± 8 *	>150	>150	<1.0	6.0	2.6
		48	120 ± 9 *	>150	22 ± 3 **	40 ± 4 **	93 ± 10 **	98 ± 12 **	>150	<0.8	5.5	3.0
		72	32 ± 3 **	117 ± 10 *	19 ± 2 **	20 ± 3 **	40 ± 3 **	57 ± 8 **	150 ± 12 *	0.3	1.7	1.6
**11**	2-CH_3_	24	>150	>150	98 ± 7 *	>150	117 ± 8 *	>150	150 ± 13 *	~1.0	>1.5	~1.0
		48	>150	>150	47 ± 5 **	>150	81 ± 10 **	>150	117 ± 9 *	~1.0	>3.2	~1.0
		72	>150	>150	19 ± 2 **	40 ± 6 **	40 ± 6 **	150 ± 11 *	98 ± 9 **	~1.0	>7.9	>3.8
**12**	4-CH_3_	24	32 ± 4 **	40 ± 5 **	35 ± 2 **	68 ± 8 **	150 ± 10 *	>150	93 ± 8 *	0.8	0.9	0.5
		48	25 ± 3 **	32 ± 5 **	29 ± 3 **	35 ± 3 **	98 ± 8 *	150 ± 13 *	57 ± 7 **	0.8	0.9	0.7
		72	22 ± 2 **	20 ± 2 **	19 ± 2 **	20 ± 2 **	43 ± 3 **	93 ± 11 *	40 ± 5 **	1.1	1.2	1.1
**13**	2,3-(CH_3_)_2_	24	>150	>150	>150	>150	79 ± 8 **	>150	55 ± 4 **	~1.0	~1.0	~1.0
		48	>150	>150	150 ± 14 *	>150	68 ± 8 **	>150	20 ± 1 **	~1.0	~1.0	~1.0
		72	>150	32 ± 5 **	40 ± 3 **	150 ± 16 *	57 ± 7 **	150 ± 15 *	19 ± 1 **	>4.7	>3.8	>1.0
**14**	2-OCH_3_	24	>150	>150	98 ± 10 *	>150	98 ± 9 *	>150	55 ± 4 **	~1.0	>1.5	~1.0
		48	>150	120 ± 12 *	79 ± 9 **	>150	43 ± 6 **	>150	43 ± 3 **	>1.3	>1.9	~1.0
		72	>150	48 ± 5 **	43 ± 5 **	>150	32 ± 5 **	>150	20 ± 3 **	>3.1	>3.5	~1.0
**15**	2-Cl	24	>150	>150	>150	>150	>150	>150	48 ± 5 **	~1.0	~1.0	~1.0
		48	>150	120 ± 10 *	57 ± 6 **	>150	98 ± 7 *	>150	20 ± 3 **	>1.3	>2.6	~1.0
		72	>150	81 ± 7 **	32 ± 3 **	>150	62 ± 5 **	>150	19 ± 2 **	>1.9	>4.7	~1.0
**16**	3-Cl	24	>150	93 ± 8 **	55 ± 5 **	>150	>150	>150	>150	>1.6	>2.7	~1.0
		48	150 ± 11 *	55 ± 6 **	29 ± 3 **	120 ± 11 *	>150	>150	>150	2.7	5.2	1.3
		72	29 ± 4 **	40 ± 3 **	20 ± 2 **	57 ± 5 **	>150	>150	62 ± 4 **	0.7	1.5	0.5
**17**	4-Cl	24	>150	>150	43 ± 4 **	>150	120 ± 9 *	150 ± 11 *	150 ± 8 *	~1.0	>3.5	~1.0
		48	>150	117 ± 7 *	23 ± 2 **	55 ± 5 **	68 ± 7 **	93 ± 9 *	98 ± 6 **	>1.3	>6.5	>2.7
		72	>150	40 ± 3 **	20 ± 3 **	20 ± 1 **	32 ± 4 **	48 ± 5 **	43 ± 4 **	>3.8	>7.5	>7.5
**18**	3,4-Cl_2_	24	79 ± 6 **	35 ± 3 **	32 ± 2 **	>150	>150	>150	55 ± 4 **	2.3	2.5	<0.5
		48	48 ± 4 **	22 ± 2 **	22 ± 3 **	35 ± 3 **	>150	>150	29 ± 3 **	2.2	2.2	1.4
		72	32 ± 4 **	20 ± 3 **	19 ± 2 **	19 ± 3 **	>150	>150 ± 13 *	19 ± 2 **	1.6	1.7	1.7
Pemetrexed		24	>150	>150	>150	>150	-	-	-	~1.0	~1.0	~1.0
		48	>150	>150	>150	>150	-	-	-	~1.0	~1.0	~1.0
		72	>150	>150	150 ± 11 **	>150		-	-	~1.0	>1.0	~1.0
Gemcitabine		24	>150	>150	>150	>150	-	-	>150	~1.0	~1.0	~1.0
		48	>150	48 ± 4 **	48 ± 6 **	>150	-	-	>150	>3.1	>3.1	~1.0
		72	120 ± 9	25 ± 2 **	20 ± 4 **	120 ± 11 **	-	-	>150	4.8	6.0	1.0

Non-tumour cell line: GMK (ECACC 88020401)—African green monkey kidney cells. Cancer cell lines (from the European Collection of Authenticated Cell Cultures): A549 (ECACC 86012804)—human Caucasian lung carcinoma cells; HeLa (ECACC 93021013)—human Negroid cervix epithelioid carcinoma cells; T47D (ECACC 85102201)—human breast carcinoma cells; MM.1R (ATCC CRL2975)—human multiple myeloma cells; Jurkat E6-1 (ECACC 88042803)—human acute T-lymphocytic leukaemia cells; HL-60 (ECACC 98070106)—human acute promyelocytic leukaemia cells. IC_50_—the half-maximal inhibitory concentration. SI—the selectivity index (expressed as the ratio of IC_50_ value of the compound in a non-tumour cell line to the IC_50_ value of the compound in a cancer cell line). Data (from five (for GMK, A549, HeLa and T47D cell lines) or two (for MM.1R, Jurkat E6-1 and HL-60 cell lines) independent experiments) are provided as the mean ± standard deviation. *, **—statistically different from the control (* *p* < 0.05; ** *p* < 0.001, Student’s *t*-test).

**Table 2:** The effect of fluorophenylated fused 1,2,4-triazinones on caspase-3, -7 and -9 levels in normal and cancer cells.

Cell Line	Compound	Concentration (ng mL^−1^)		
		Caspase-3	Caspase-7	Caspase-9
GMK	Control	1.473 ± 0.1	1.069 ± 0.3	1.042 ± 0.07
	**11**	1.444 ± 0.2	1.493 ± 0.1	1.047 ± 0.1
	**13**	1.467 ± 0.1	1.564 ± 0.2	1.022 ± 0.2
	**14**	1.435 ± 0.2	1.659 ± 0.1 *	1.039 ± 0.2
	**15**	1.426 ± 0.1	2.052 ± 0.4 *	1.032 ± 0.4
	**17**	1.462 ± 0.1	2.052 ± 0.3 *	1.056 ± 0.2
A549	Control	1.641 ± 0.3	1.058 ± 0.2	1.056 ± 0.1
	**11**	1.843 ± 0.2	1.093 ± 00.5	2.084 ± 0.3 *
	**13**	1.933 ± 0.2	2.099 ± 0.2 *	3.073 ± 0.3 *
	**14**	1.922 ± 0.2	2.089 ± 0.05 *	3.073 ± 0.2 *
	**15**	2.747 ± 0.2 *	2.090 ± 0.3 *	3.092 ± 0.3 *
	**17**	2.779 ± 0.05 *	2.091 ± 0.1 *	3.083 ± 0.2 *
HeLa	Control	1.270 ± 0.1	1.015 ± 0.1	1.044 ± 0.2
	**11**	1.439 ± 0.2	2.071 ± 0.2 *	3.066 ± 0.4 *
	**13**	1.530 ± 0.2	2.070 ± 0.2 *	3.074 ± 0.3 *
	**14**	1.527 ± 0.3	2.085 ± 0.06 *	3.062 ± 0.4 *
	**15**	2.499 ± 0.3 *	2.096 ± 0.1 *	3.080 ± 0.3 *
	**17**	2.321 ± 0.2 *	2.094 ± 0.2 *	3.094 ± 0.4 *
T47D	Control	1.284 ± 0.2	1.018 ± 0.1	2.055 ± 0.2
	**11**	1.302 ± 0.1	2.060 ± 0.08 *	3.075 ± 0.4 *
	**13**	2.348 ± 0.1 *	2.075 ± 0.1 *	3.074 ± 0.3 *
	**14**	2.376 ± 0.4 *	3.089 ± 0.3 *	4.093 ± 0.6 *
	**15**	2.398 ± 0.3 *	3.085 ± 0.2 *	4.078 ± 0.4 *
	**17**	2.394 ± 0.2 *	3.083 ± 0.3 *	4.074 ± 0.3 *

Non-tumour cell line (from the European Collection of Authenticated Cell Cultures): GMK (ECACC 88020401)—African green monkey kidney cells. Cancer cell lines (from the European Collection of Authenticated Cell Cultures): A549 (ECACC 86012804)—human Caucasian lung carcinoma cells; HeLa (ECACC 93021013)—human Negroid cervix epithelioid carcinoma cells; T47D (ECACC 85102201)—human breast carcinoma cells. Compounds were tested at a concentration of 150 μM. Data (from three independent experiments) are shown as the mean ± standard deviation. * statistically significantly different from the control (*p* < 0.05, Student’s *t*-test).

**Table 3:** The calculated binding affinities of fluorophenylated fused 1,2,4-triazinones (**10**–**18**) at human adenosine A_2A_ (PDB ID: 3EML) and A_2B_ (PDB ID: 8HDO) receptors.

General Structure	Compound	R	Binding Affinity (kcal/mol)	
			A_2A_ AR	A_2B_ AR
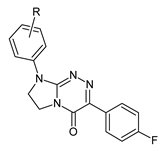	**10**	H	−9.5	−8.4
	**11**	2-CH_3_	−9.8	−8.4
	**12**	4-CH_3_	−9.6	−8.7
	**13**	2,3-(CH_3_)_2_	−9.9	−8.8
	**14**	2-OCH_3_	−9.4	−8.3
	**15**	2-Cl	−9.7	−8.6
	**16**	3-Cl	−9.9	−8.6
	**17**	4-Cl	−9.6	−8.7
	**18**	3,4-Cl_2_	−9.8	−8.9

The calculated binding affinity for ZM-241385 (an adenosine A_2A_ receptor antagonist) is −8.9 kcal/mol, whereas for PSB-1115 (an adenosine A_2B_ receptor antagonist) it is −8.3 kcal/mol.

**Table 4:** Summary of the structural profiling for the complexes of ligands **10**–**18** within the adenosine A_2A_ (PDB ID: 3EML) and A_2B_ (PDB ID: 8HDO) receptors.

Compound	Adenosine A_2A_ Receptor		Adenosine A_2B_ Receptor	
	Contacts Count	Crucial Interactions and Geometrical Features	Contacts Count	Crucial Interactions and Geometrical Features
**10**	4	Ala63, Val84, Tyr271, Ile274	5	Ile67, Ser68, Leu172, Val250, Ile276
**11**	6	Ala63, Val84, Phe168, Glu169, Tyr271, Ile274	4	Tyr10, Ser68, Val250, Ile276
**12**	7	Ala63, Ile66, Val84, multivalent Leu167, Phe168, Ile274	4	Ile67, Ser68, Leu172, Val250
**13**	8	Ala63, Ile66, Leu167, multivalent Phe168, Glu169, Leu267, Tyr271	6	Tyr10, Ser68, Leu172, Phe173, Val250, Ile276
**14**	5	Ala63, Val84, Phe168, Glu169, Tyr271	3	π-cation with Phe173; multivalent Val250 (incl. 3.39 Å), Ile276
**15**	5	Ala63, Ile66, Phe168, Leu267, Tyr271 (3.37 Å)	4	Tyr10, Ser68, Leu86, Ile276
**16**	6	Ala63, Ile66, Val84, Phe168, Glu169, Tyr271 (3.59 Å)	5	Ile67, Leu172 (3.41 Å), Phe173, Trp247, Ile276
**17**	7	π-π Stacking with Phe168 (4.19 Å); Ala63, Ile66, Val84, Phe168, Glu169, Tyr271, Ile274	6	Ile67, Ser68, Leu172, Phe173, Trp247 (3.58 Å), Val250
**18**	5	Ala63, Ile66, Val84, Phe168, Tyr271	3	Leu172, Trp247 (3.76 Å), Val250

**Table 5:** Risk assessment of adverse side effects for fluorophenylated fused 1,2,4-triazinones (**10**–**18**).

Compound	Mutagenicity	Tumorigenicity	Irritating Effects	Reproductive Effects
**10**	1.0	1.0	1.0	1.0
**11**	1.0	1.0	1.0	1.0
**12**	1.0	1.0	1.0	1.0
**13**	1.0	1.0	0.6	1.0
**14**	1.0	1.0	1.0	1.0
**15**	1.0	1.0	1.0	1.0
**16**	1.0	1.0	1.0	1.0
**17**	1.0	1.0	1.0	1.0
**18**	1.0	1.0	1.0	1.0
Pemetrexed	1.0	1.0	1.0	1.0
Gemcitabine	1.0	0.6	1.0	1.0

Score: 1.0—no risk; 0.6—high risk.

**Table 6:** In silico descriptors assessing drug-likeness of fluorophenylated fused 1,2,4-triazinones (**10**–**18**).

Compound	MW	HBD	HBA	log *P*	nRB
**10**	308.316	0	5	2.5961	2
**11**	322.343	0	5	2.9045	2
**12**	322.343	0	5	2.9045	2
**13**	336.370	0	5	3.2129	2
**14**	338.342	0	6	2.6047	3
**15**	342.761	0	5	3.2495	2
**16**	342.761	0	5	3.2495	2
**17**	342.761	0	5	3.2495	2
**18**	377.206	0	5	3.9029	2

MW (in g mol^−1^)—molecular weight; HBD—hydrogen bond donors in total; HBA—hydrogen bond acceptors in total; log *P* (in log mol L^−1^)—*n*-octanol/water partition coefficient; nRB—the number of rotatable bonds.

**Table 7:** In silico parameters characterising the adsorption, distribution, metabolism and excretion processes of fluorophenylated fused 1,2,4-triazinones (**10**–**18**).

Parameter	10	11	12	13	14	15	16	17	18	Pemetrexed	Gemcitabine
HIA	98.757	98.701	98.692	99.474	99.286	96.180	97.919	97.434	97.055	15.398	68.491
P_Caco-2_	1.353	1.358	1.364	1.354	1.323	1.439	1.394	1.376	1.107	−1.162	−0.053
log *S*	−3.445	−3.830	−3.823	−3.974	−3.975	−4.043	−3.959	−3.929	−4.485	−2.879	−2.749
log *K*_p_	−2.572	−2.680	−2.681	−2.688	−2.735	−2.554	−2.598	−2.610	−2.650	−2.735	−2.810
P-gp substate	no	no	no	no	no	no	no	no	no	yes	no
P-gp I inhibitor	no	no	no	no	no	yes	yes	yes	yes	no	no
P-gp II inhibitor	yes	yes	yes	yes	yes	yes	yes	yes	yes	no	no
VDss	−0.159	−0.109	−0.081	0.014	−0.297	−0.257	−0.048	−0.097	0.044	−0.032	−0.283
Fu	0.172	0.219	0.222	0.260	0.211	0.101	0.153	0.188	0.149	0.381	0.788
log BB	0.853	0.480	0.457	0.436	−0.019	0.901	0.816	0.810	0.786	−1.587	−0.878
log PS	−2.124	−2.034	−2.043	−1.964	−2.379	−1.979	−2.011	−2.002	−1.891	−3.916	−3.610
CYP2D6 substrate	no	no	no	no	no	no	no	no	no	no	no
CYP3A4 substrate	yes	yes	yes	yes	yes	yes	yes	yes	yes	no	no
CYP1A2 inhibitor	yes	yes	yes	yes	yes	yes	yes	yes	yes	no	no
CYP2C19 inhibitor	yes	yes	yes	yes	yes	yes	yes	yes	yes	no	no
CYP2C9 inhibitor	yes	yes	yes	yes	yes	yes	yes	yes	yes	no	no
CYP2D6 inhibitor	no	no	no	no	no	no	no	no	no	no	no
CYP3A4 inhibitor	no	no	no	no	no	no	no	no	yes	no	no
TC	−0.041	−0.003	−0.015	0.004	−0.038	−0.080	−0.287	−0.349	−0.164	0.189	0.415
OCT2 substrate	yes	yes	yes	yes	no	yes	yes	yes	yes	no	no

HIA (in %)—human intestinal absorption; P_Caco-2_ (×10^−6^ cm s^−1^)—the logarithm of the apparent permeability coefficient; log *S* (in log mol L^−1^)—water solubility in logarithmic scale; log *K*_p_—the logarithmic skin permeability constant; P-gp—P-glycoprotein; VDss (in log L kg^−1^)—steady state volume of distribution; Fu (fraction unbound)—fraction of a drug unbound in human blood plasma; log BB—the logarithmic ratio of brain to plasma drug concentration; log PS (CNS permeability)—the blood-brain permeability; CYP2D6 and CYP3A4—isoforms of cytochrome P-450 substrate; CYP1A2, CYP2C19, CYP2C9, CYP2D6, CYP3A4—isoforms of cytochrome P-450 inhibitor; TC (in log mL min^−1^ kg^−1^)—total clearance, OCT2 substrate—renal organic cation transporter 2 substrate.

**Table 8:** Logarithmic retention factors (logs *k*) measured in an acetonitrile–water mobile phase by RP-HPLC on an ODS-2 column as well as logarithmic partition coefficients computed in silico for fluorophenylated fused 1,2,4-triazinones (**10**–**18**).

Compound	log *k*	Alog P_s_	AClog P	Alog P	Mlog P	xlog P2	xlog P2	log *P*_average_	log *P*_HSA_
**10**	−0.006	2.19	4.07	3.65	3.83	4.45	2.61	3.47 (±0.88)	0.565
**11**	−0.135	2.37	4.38	4.14	4.07	4.68	2.97	3.77 (±0.90)	0.839
**12**	0.098	2.37	4.38	4.14	4.07	4.89	2.97	3.80 (±0.94)	0.839
**13**	−0.058	2.56	4.70	4.63	4.30	5.12	3.34	4.11 (±0.97)	1.105
**14**	−0.159	2.24	3.96	3.64	3.30	4.37	2.58	3.30 (±0.82)	0.499
**15**	−0.090	2.88	4.68	4.32	4.07	5.08	3.24	4.04 (±0.84)	0.880
**16**	0.215	2.82	4.68	4.32	4.07	5.08	3.24	4.03 (±0.86)	0.966
**17**	0.182	2.77	4.68	4.32	4.07	5.08	3.24	4.03 (±0.87)	0.886
**18**	0.392	2.97	5.29	4.98	4.30	5.70	3.86	4.52 (±1.01)	1.267

Chromatographic values: log *k*—logarithm of the retention factor. Computational values: log *P*_average_—the logarithmic *n*-octanol-water partition coefficient (±standard error) computed in silico as an average value from different computational methods for estimating log *P* (Alog P_s_, AClog P, Alog P, Mlog P, xlog P2 and xlog P3). These calculations take into account the increments of separate atoms, fragments, intramolecular interactions on the log *P*. All partition coefficients were computed from the molecular structures of all investigated compounds employing an accessible online software at the Virtual Computational Chemistry Laboratory, version 2005 [80,81]. The pharmacokinetic descriptor: log *P*_HSA_—the logarithmic partition coefficient of a particular solute between human serum albumin and water; it was computed according to the linear solvation energy relationships (LSERs) equation—the same as that originally developed by Valko et al. [82] using an ACD/Percepta software, version 1994-2012 (ACD/Labs, Advanced Chemistry Development, Inc., Toronto, ON, Canada).

## Data Availability

Data are contained within the article or Supplementary Material.

## Supplementary Materials

The following supporting information can be downloaded at: https://www.mdpi.com/article/10.3390/ijms27188388/s1.
