# Supplementary material for: Novel Fluorophenylated Dihydroimidazotriazinones as Potential Anticancer Agents: Design, Synthesis, In Vitro, Ex Vivo and In Silico Characterisation
Source: Int J Mol Sci. 2026 Sep 20;27(18):8388. doi: 10.3390/ijms27188388 (PMC13607122; doi:10.3390/ijms27188388)
Supplement: Supplementary file 1 [file ijms-27-08388-s001.zip › ijms-4458839-supplementary.pdf]

International Journal of *Molecular Sciences*

MDPI

Supplementary material

Novel fluorophenylated dihydroimidazotriazinones as potential anticancer agents: design, synthesis, *in vitro*, *ex vivo* and *in silico* characterisation

Małgorzata Sztanke [1]*, Jolanta Rzymowska [2], Małgorzata Janicka [3], Weronika Sofińska-Chmiel [4] and Krzysztof Sztanke [5]*

1 Department of Medical Chemistry, Medical University of Lublin, 4A Chodźki Street, 20-093 Lublin, Poland
2 Department of Biology and Genetics, Medical University of Lublin, 4A Chodźki Street, 20-093 Lublin, Poland
3 Department of Physical Chemistry, Institute of Chemical Science, Faculty of Chemistry, Maria Curie-Skłodowska University, Maria Curie-Skłodowska Sq. 3, 20-031 Lublin, Poland
4 Analytical Laboratory, Institute of Chemical Science, Faculty of Chemistry, Maria Curie-Skłodowska University, Maria Curie-Skłodowska Sq. 3, 20-031 Lublin, Poland
5 Laboratory of Bioorganic Compounds Synthesis and Analysis, Medical University of Lublin, 4A Chodźki Street, 20-093 Lublin, Poland
* Correspondence: malgorzata.sztanke@umlub.edu.pl (M.S.); krzysztof.sztanke@umlub.edu.pl (K.S.)

**Figure S1.** HPLC chromatogram for compound **10**	3
**Figure S2.** HPLC chromatogram for compound **11**	3
**Figure S3.** HPLC chromatogram for compound **12**	3
**Figure S4.** HPLC chromatogram for compound **13**	4
**Figure S5.** HPLC chromatogram for compound **14**	4
**Figure S6.** HPLC chromatogram for compound **15**	4
**Figure S7.** HPLC chromatogram for compound **16**	5
**Figure S8.** HPLC chromatogram for compound **17**	5
**Figure S9.** HPLC chromatogram for compound **18**	5
**Figure S10.** ^{1}H NMR spectrum in DMSO-d_6 for compound **10**	6
**Figure S11.** ^{1}H NMR spectrum in DMSO-d_6 for compound **11**	7
**Figure S12.** ^{1}H NMR spectrum in DMSO-d_6 for compound **12**	8
**Figure S13.** ^{1}H NMR spectrum in DMSO-d_6 for compound **13**	9
**Figure S14.** ^{1}H NMR spectrum in DMSO-d_6 for compound **14**	10
**Figure S15.** ^{1}H NMR spectrum in DMSO-d_6 for compound **15**	11
**Figure S16.** ^{1}H NMR spectrum in DMSO-d_6 for compound **16**	12
**Figure S17.** ^{1}H NMR spectrum in DMSO-d_6 for compound **17**	13
**Figure S18.** ^{1}H NMR spectrum in DMSO-d_6 for compound **18**	14
**Figure S19.** ^{13}C NMR spectrum in DMSO-d_6 for compound **10** (A), and expanded regions of the aromatic (B) and aliphatic (C) ranges with peak values	15
**Figure S20.** ^{13}C NMR spectrum in DMSO-d_6 for compound **11** (A), and expanded regions of the aromatic (B) and aliphatic (C) ranges with peak values	17
**Figure S21.** ^{13}C NMR spectrum in DMSO-d_6 for compound **12** (A), and expanded regions of the aromatic (B) and aliphatic (C) ranges with peak values	19
**Figure S22.** ^{13}C NMR spectrum in DMSO-d_6 for compound **13** (A), and expanded regions of the aromatic (B) and aliphatic (C) ranges with peak values	21

**Figure S23.** ^{13}C NMR spectrum in DMSO-d_6 for compound **14** (A), and expanded regions of the aromatic (B) and aliphatic (C) ranges with peak values	**23**
**Figure S24.** ^{13}C NMR spectrum in DMSO-d_6 for compound **15** (A), and expanded regions of the aromatic (B) and aliphatic (C) ranges with peak values	**25**
**Figure S25.** ^{13}C NMR spectrum in DMSO-d_6 for compound **16** (A), and expanded regions of the aromatic (B) and aliphatic (C) ranges with peak values	**27**
**Figure S26**. ^{13}C NMR spectrum in DMSO-d_6 for compound **17** (A), and expanded regions of the aromatic (B) and aliphatic (C) ranges with peak values	**29**
**Figure S27.** ^{13}C NMR spectrum in DMSO-d_6 for compound **18** with peak values	**31**
**Figure S28.** ATR-IR spectrum for compound **10**	**32**
**Figure S29.** ATR-IR spectrum for compound **11**	**32**
**Figure S30.** ATR-IR spectrum for compound **12**	**33**
**Figure S31.** ATR-IR spectrum for compound **13**	**33**
**Figure S32.** ATR-IR spectrum for compound **14**	**34**
**Figure S33.** ATR-IR spectrum for compound **15**	**34**
**Figure S34.** ATR-IR spectrum for compound **16**	**35**
**Figure S35.** ATR-IR spectrum for compound **17**	**35**
**Figure S36.** ATR-IR spectrum for compound **18**	**36**
**Table S1.** Cytotoxicity of fluorophenylated fused 1,2,4-triazinones **10-18** in non-tumour and tumour cells of epithelial origin	**37**
**Table S2.** Cytotoxicity of fluorophenylated fused 1,2,4-triazinones **10-18** in MM.1R, Jurkat E6-1 and HL-60 cells	**38**

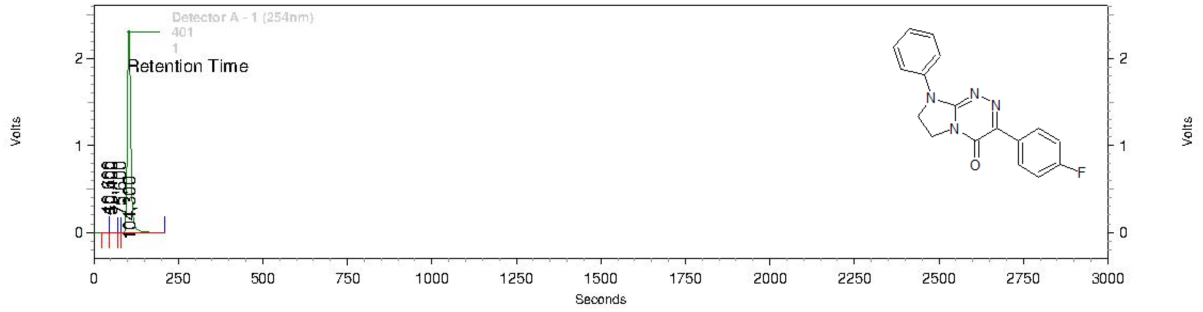

Detector A - 1 (254nm)

Pk #	Retention Time	Area	Area %	Height	Height %
1	40,600	22255	0,080	2851	0,123
2	50,400	21682	0,078	2161	0,093
3	75,600	206	0,001	39	0,002
4	104,300	27796770	99,841	2318772	99,783
Totals		27840913	100,000	2323823	100,000

Figure S1. HPLC chromatogram for compound **10**.

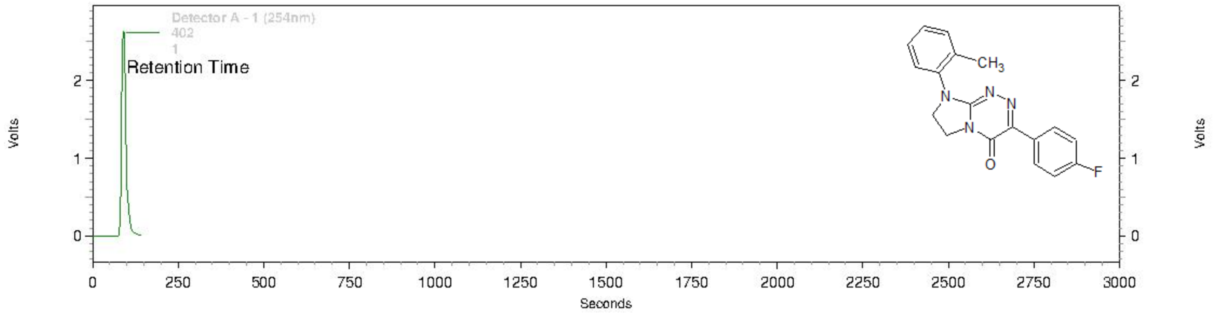

Figure S2. HPLC chromatogram for compound **11**.

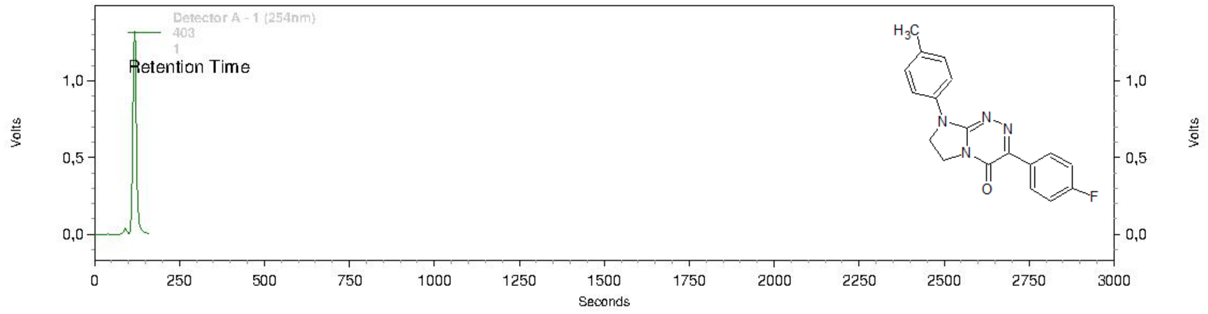

Figure S3. HPLC chromatogram for compound **12**.

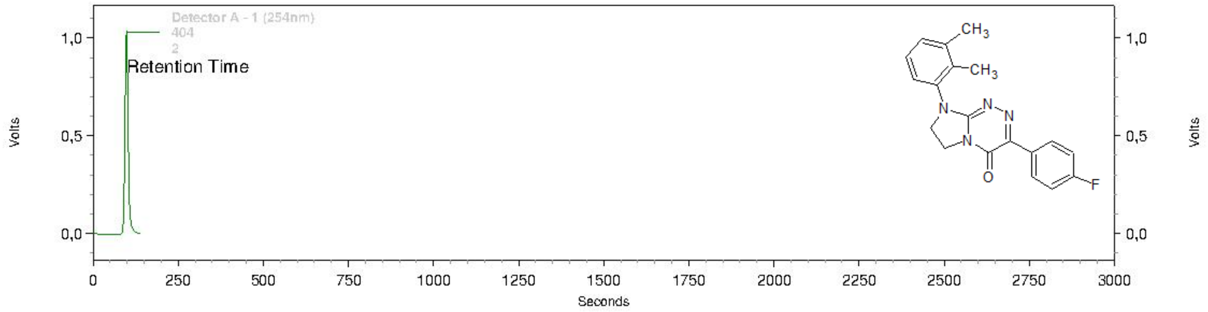

Figure S4. HPLC chromatogram for compound **13**.

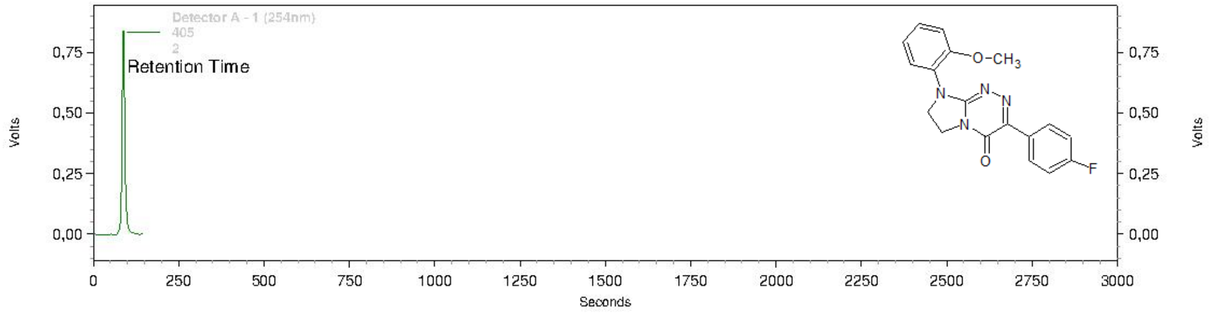

Figure S5. HPLC chromatogram for compound **14**.

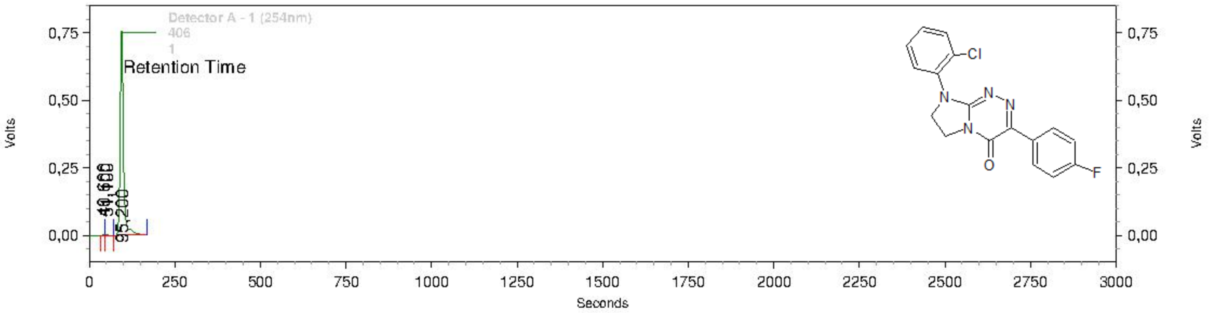

Detector A - 1 (254nm)

Pk #	Retention Time	Area	Area %	Height	Height %
1	40,600	9179	0,106	1191	0,157
2	51,100	16260	0,188	1580	0,208
3	95,200	8621191	99,706	755797	99,635
Totals		8646630	100,000	758568	100,000

Figure S6. HPLC chromatogram for compound **15**.

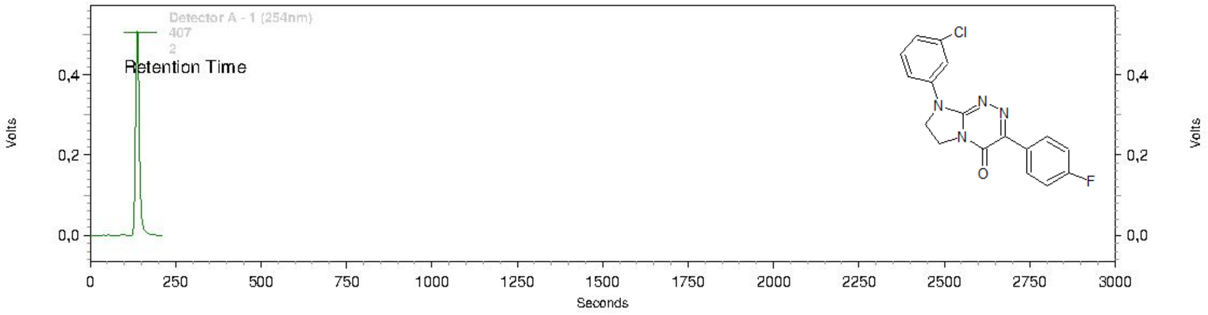

Figure S7. HPLC chromatogram for compound **16**.

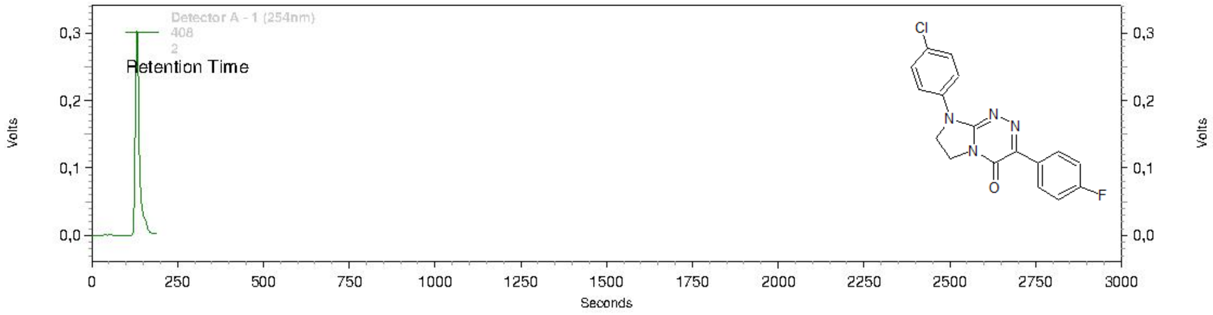

Figure S8. HPLC chromatogram for compound **17**.

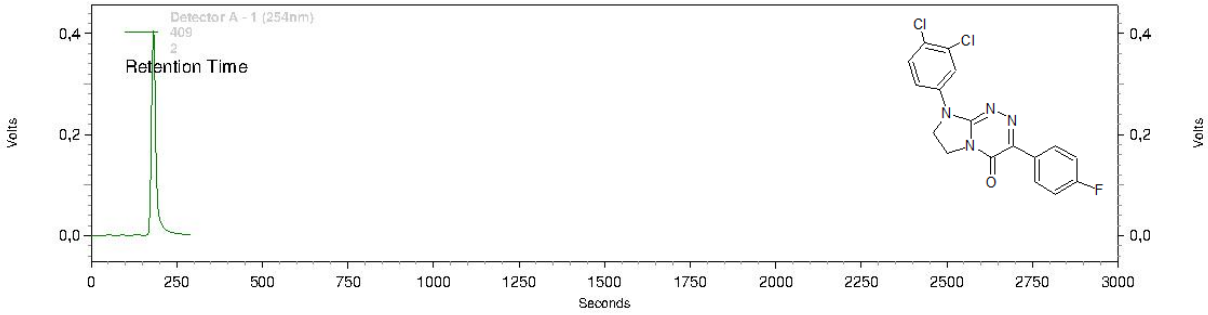

Figure S9. HPLC chromatogram for compound **18**.

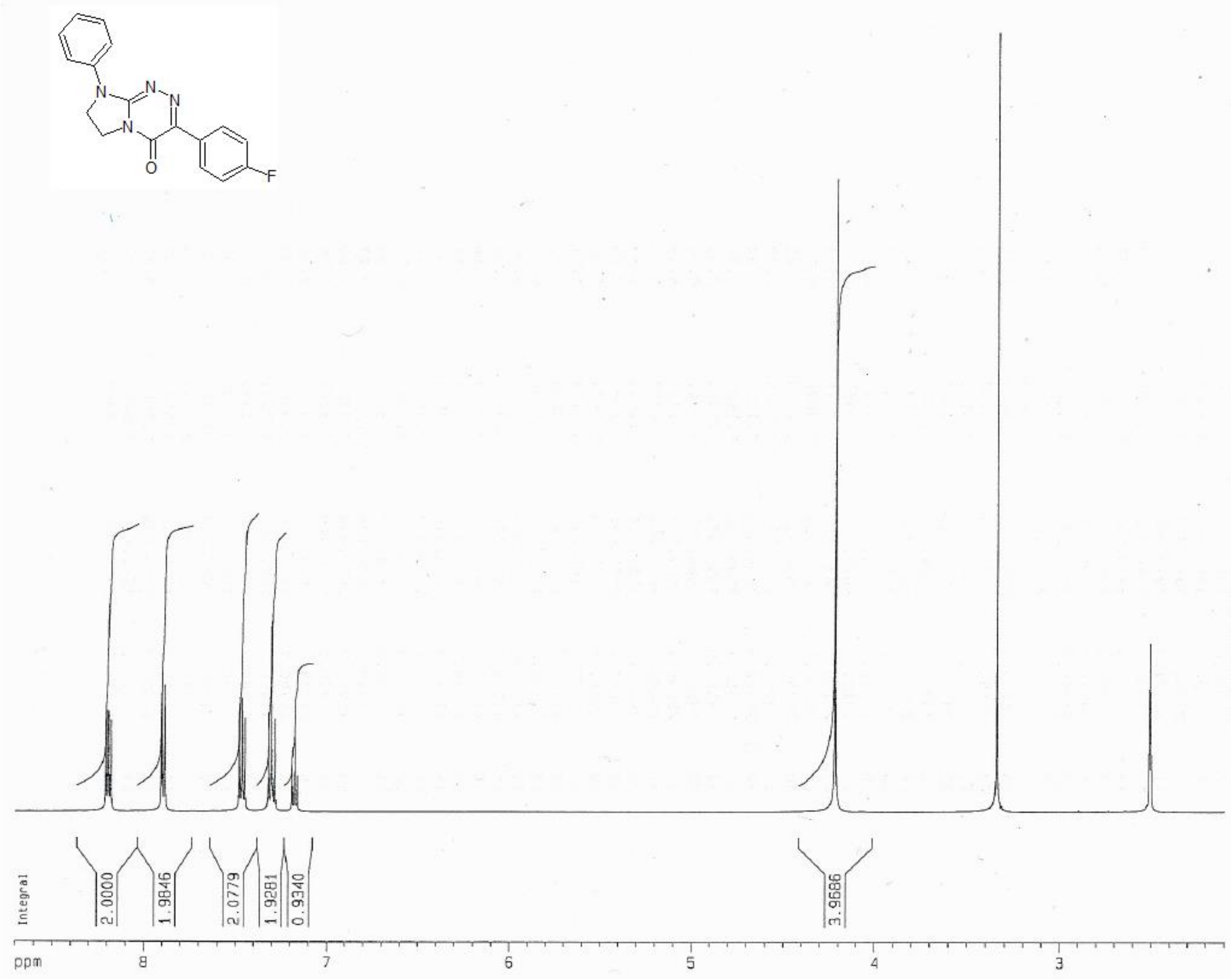

Figure S10. ^{1}H NMR spectrum in DMSO-d_6 for compound **10.**

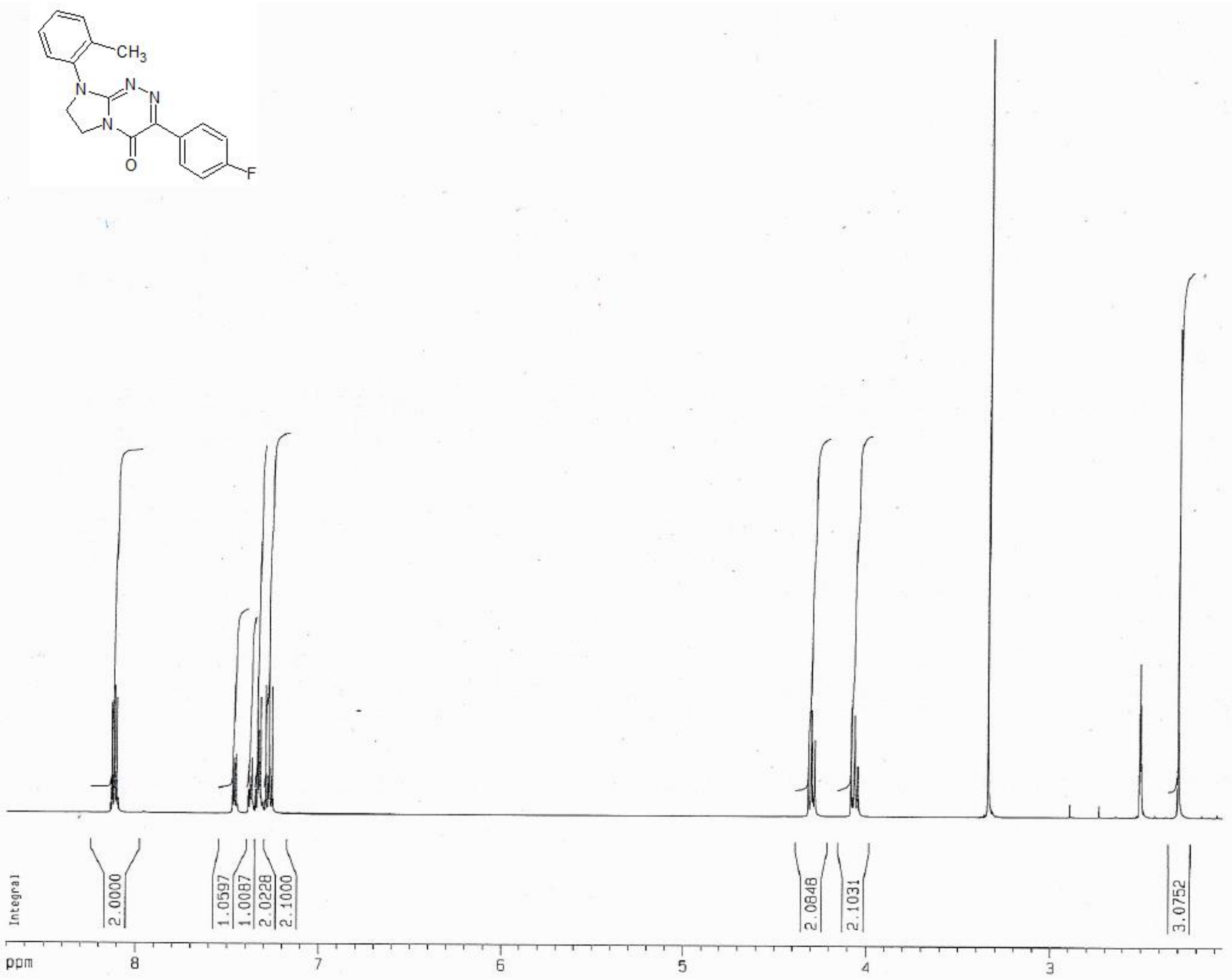

Figure S11. ^{1}H NMR spectrum in DMSO-d_6 for compound **11.**

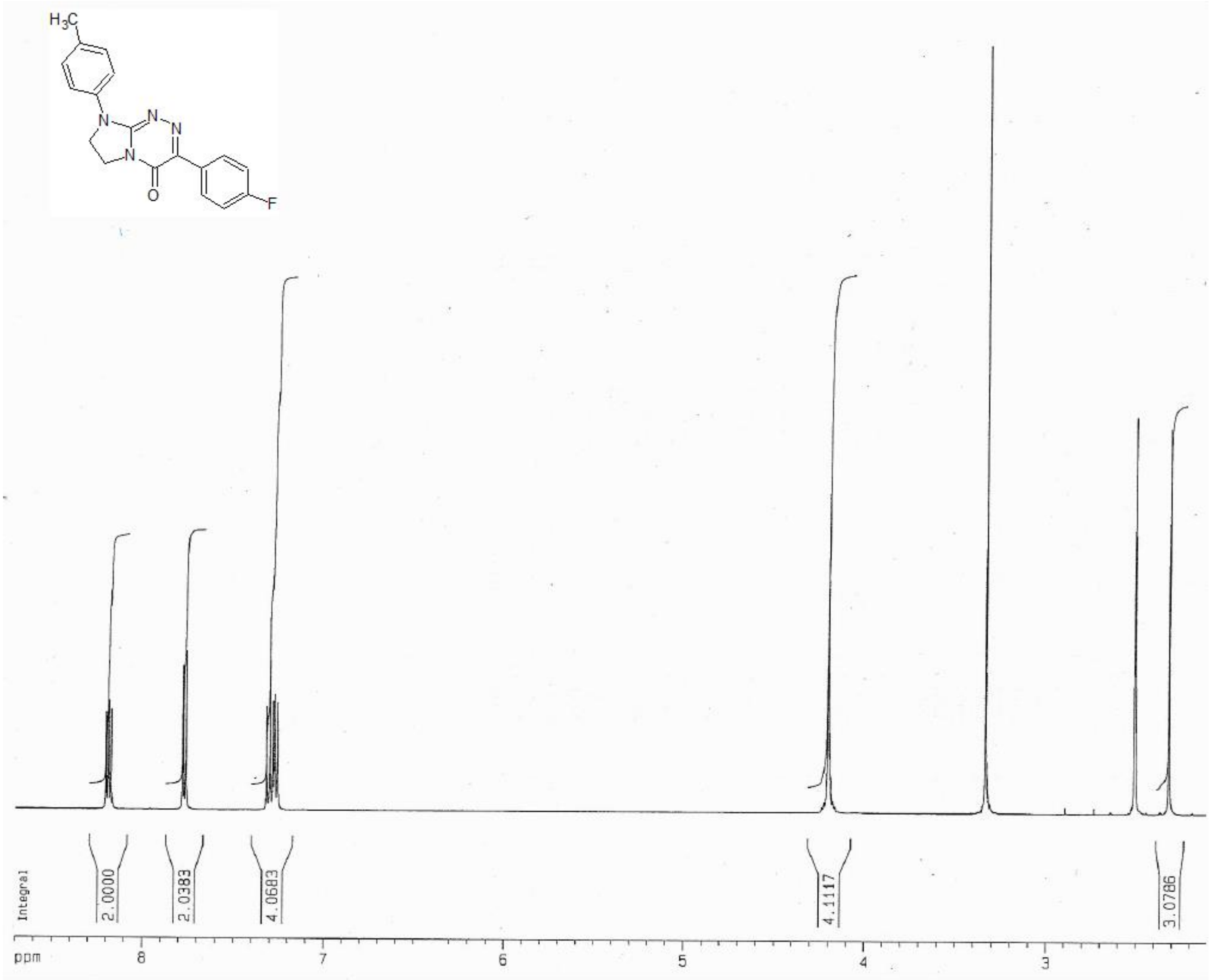

Figure S12. ^{1}H NMR spectrum in DMSO-d_6 for compound **12.**

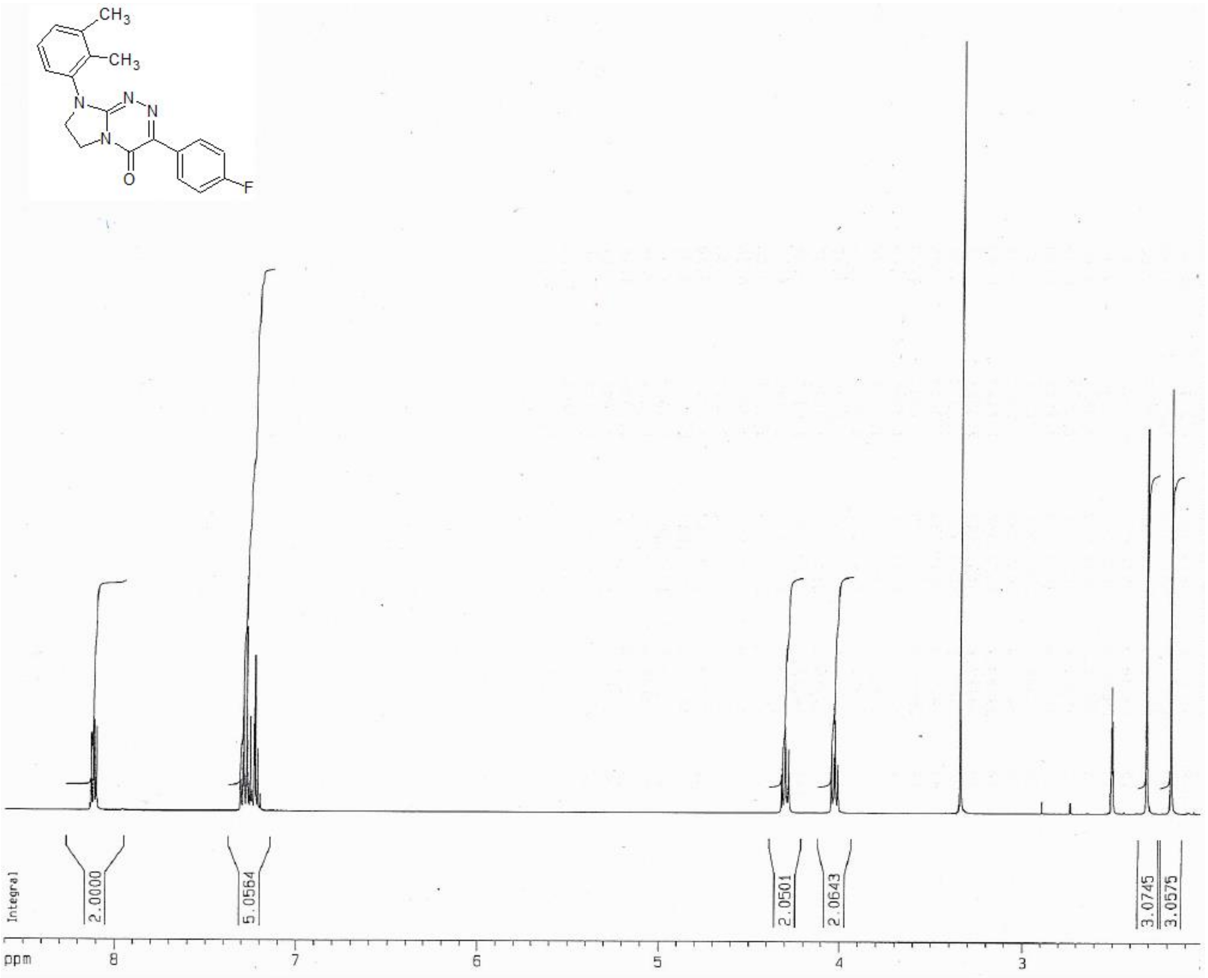

Figure S13. ^{1}H NMR spectrum in DMSO-d_6 for compound **13.**

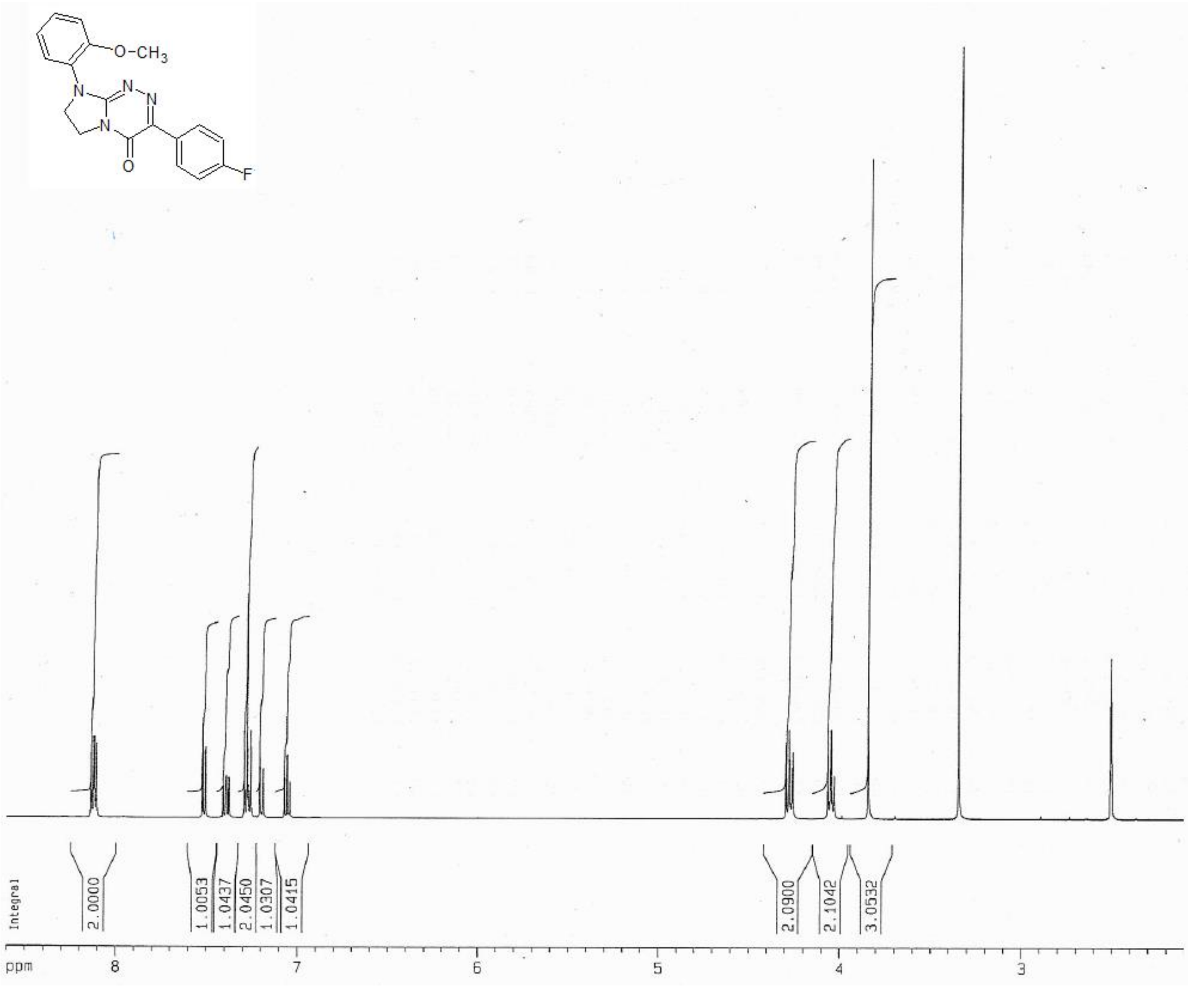

Figure S14. ^{1}H NMR spectrum in DMSO-d_6 for compound **14.**

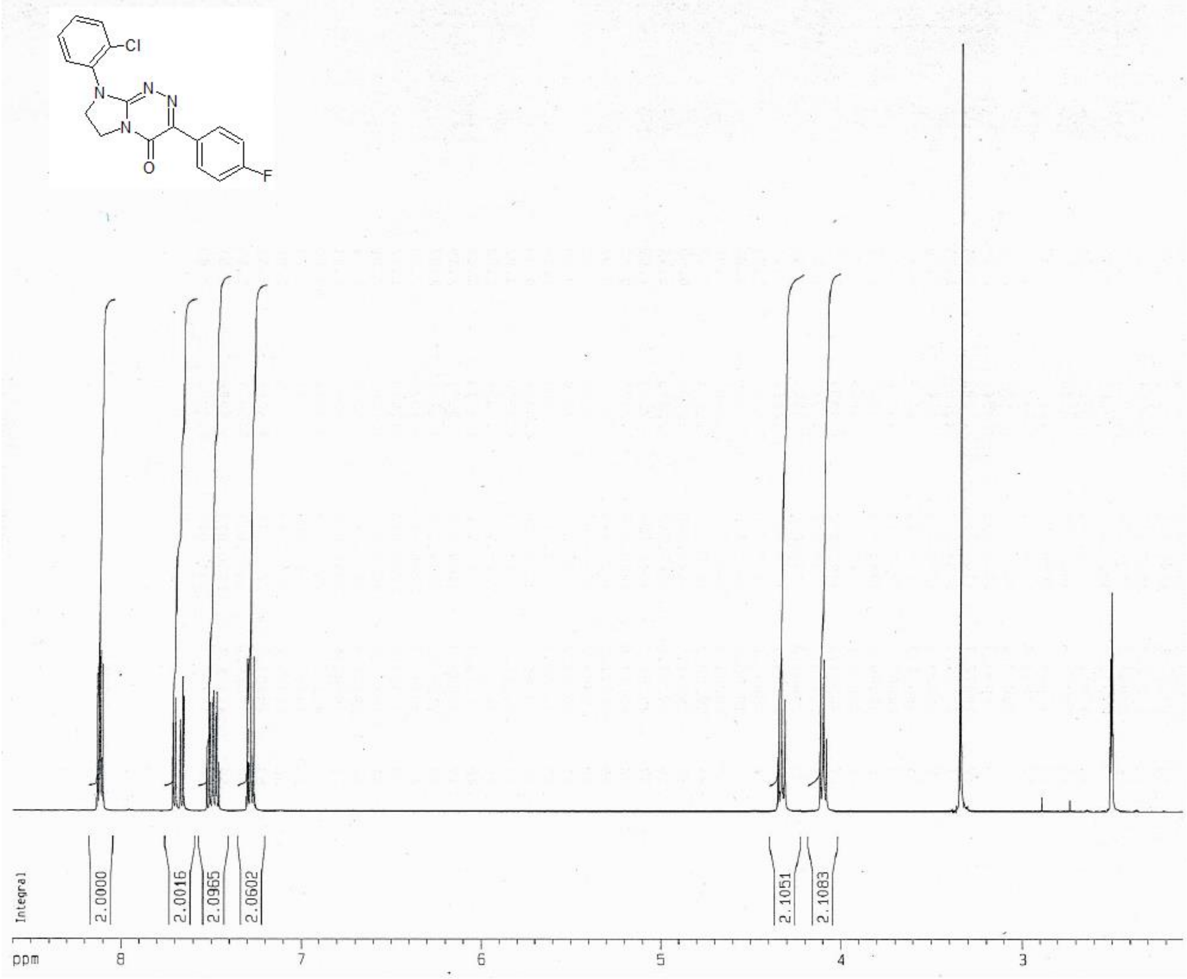

Figure S15. ^{1}H NMR spectrum in DMSO-d_6 for compound **15.**

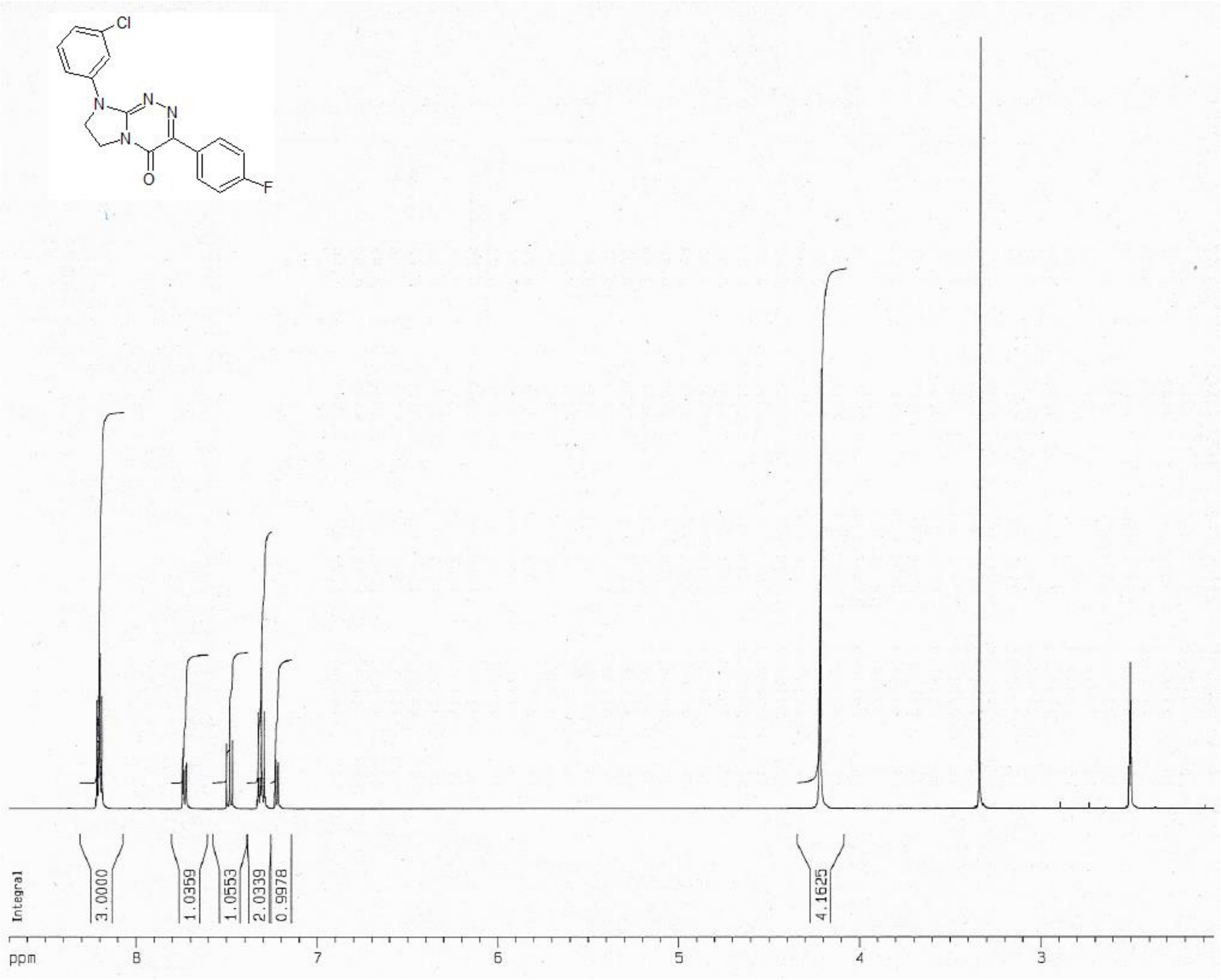

Figure S16. ^{1}H NMR spectrum in DMSO-d_6 for compound **16.**

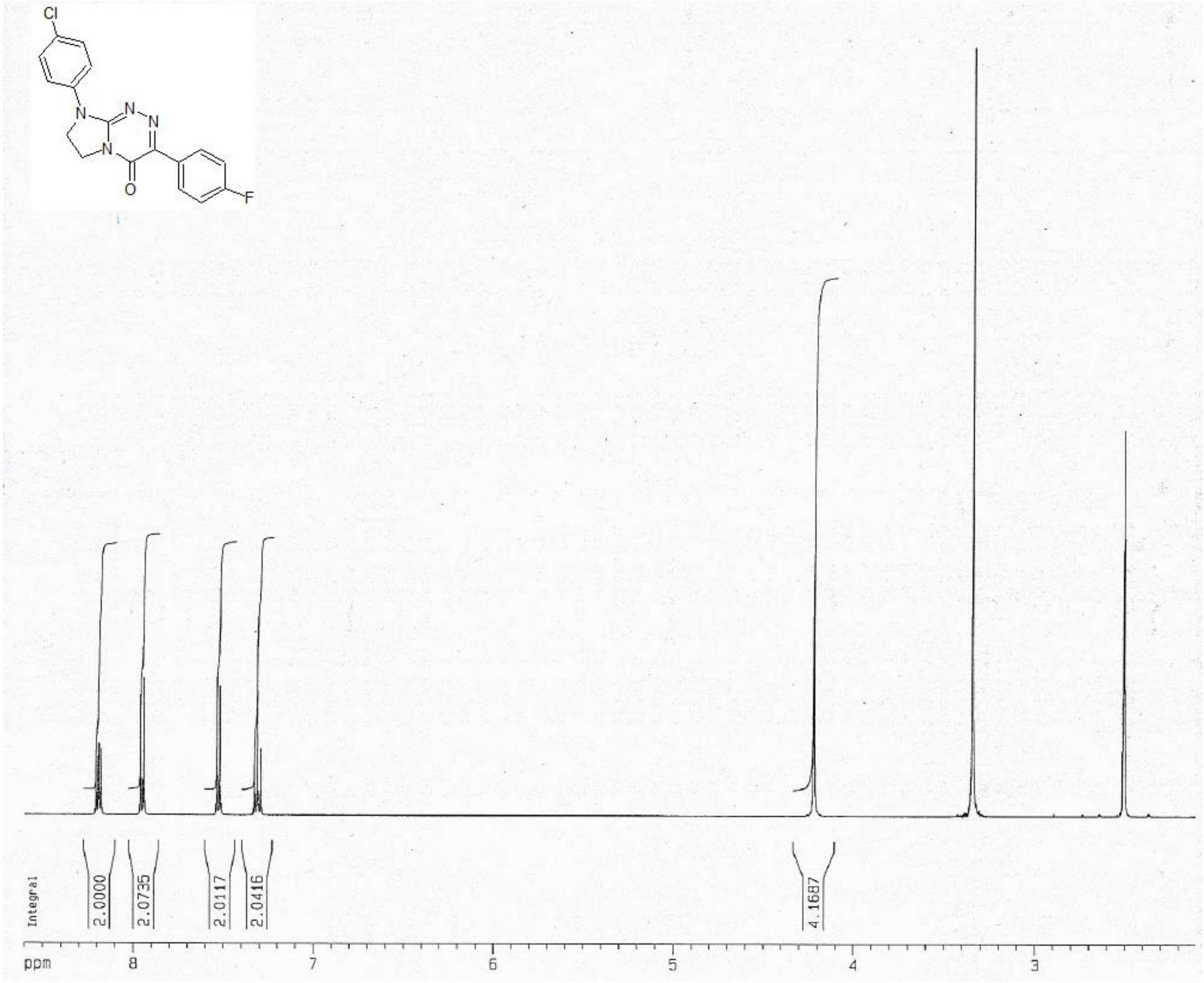

Figure S17. ^{1}H NMR spectrum in DMSO-d_6 for compound **17.**

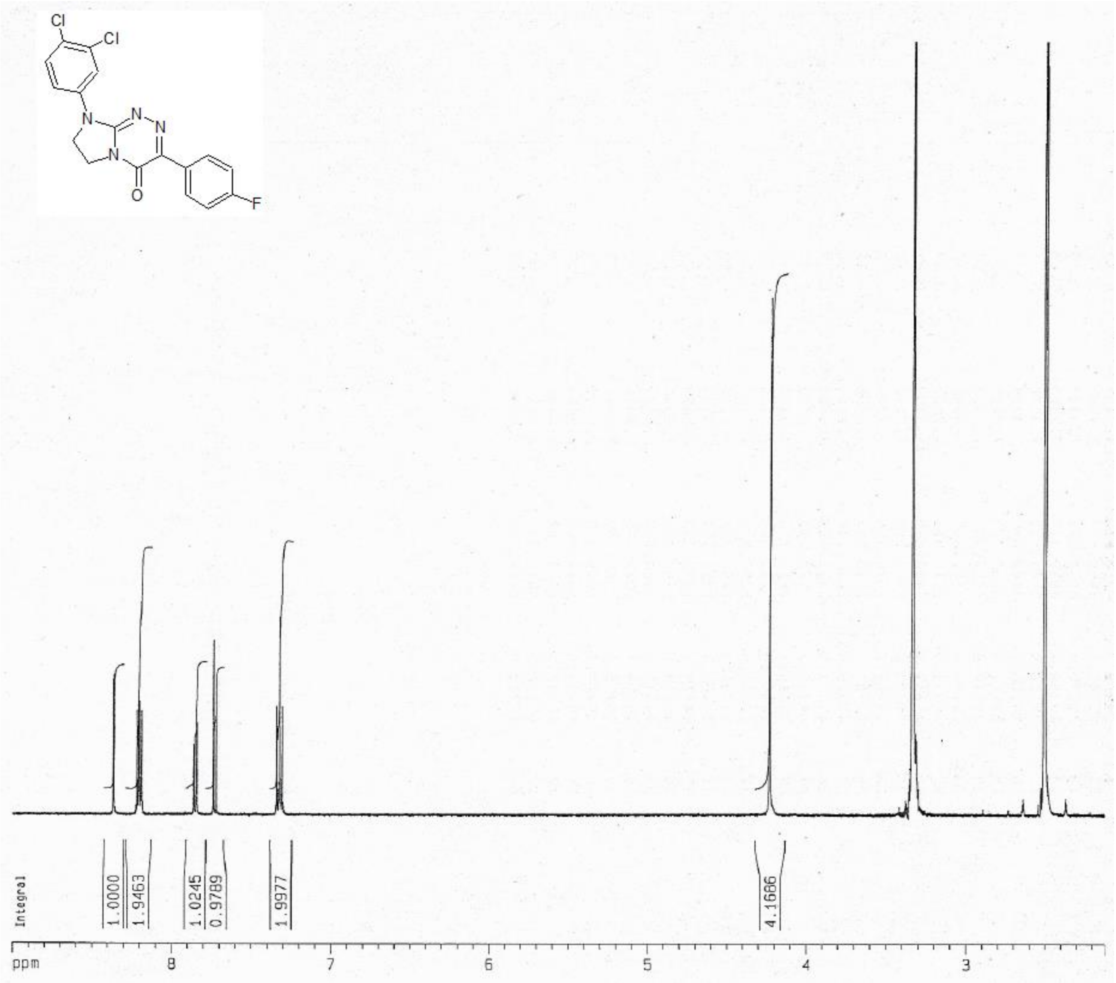

Figure S18. ^{1}H NMR spectrum in DMSO-d_6 for compound **18.**

A.

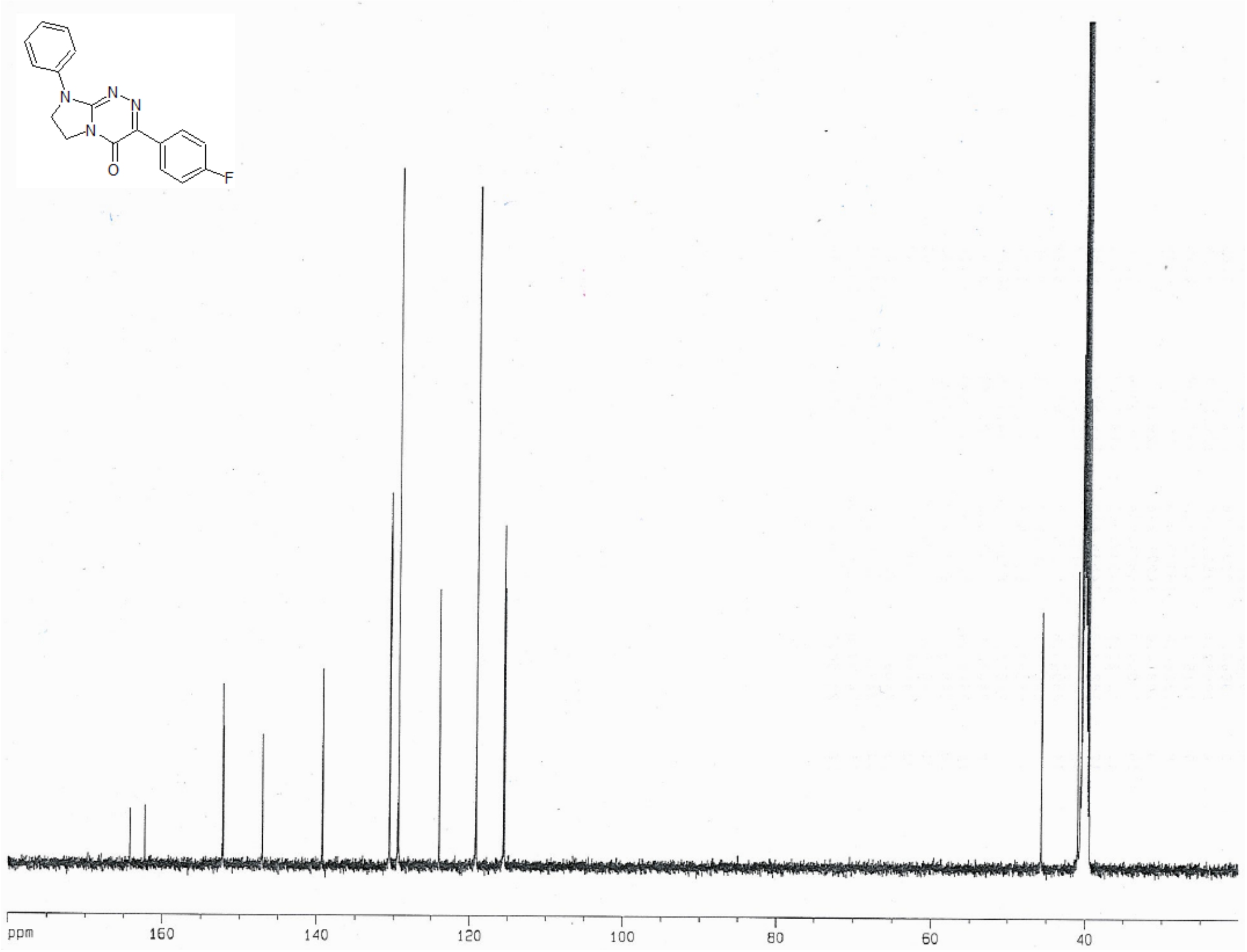

B.

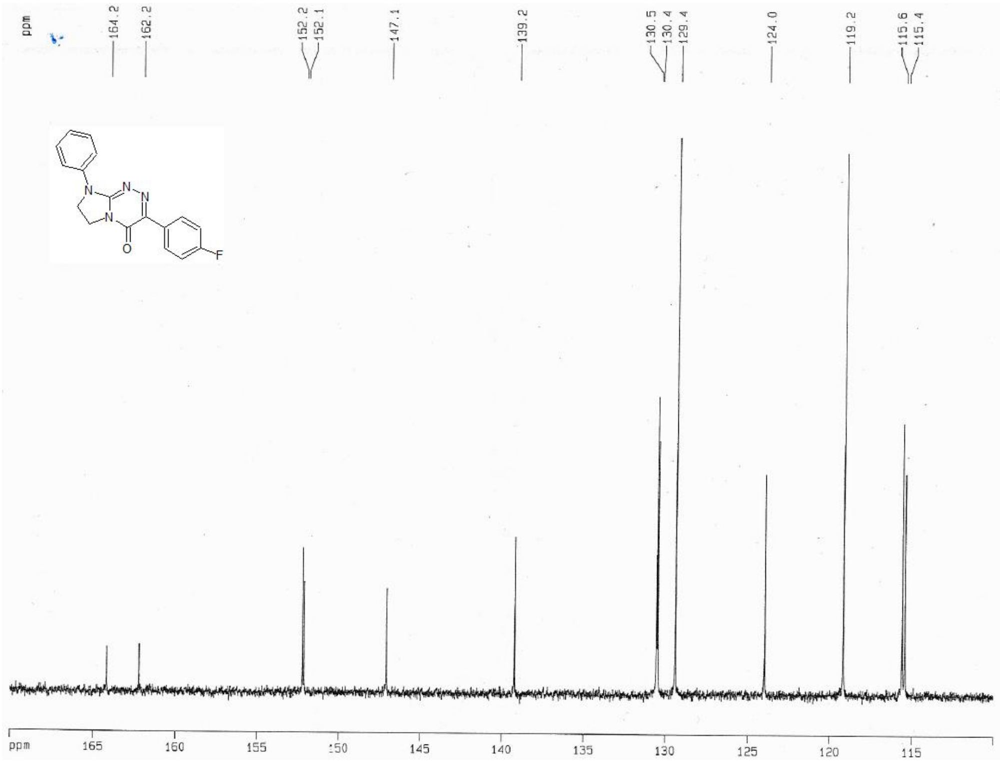

C.

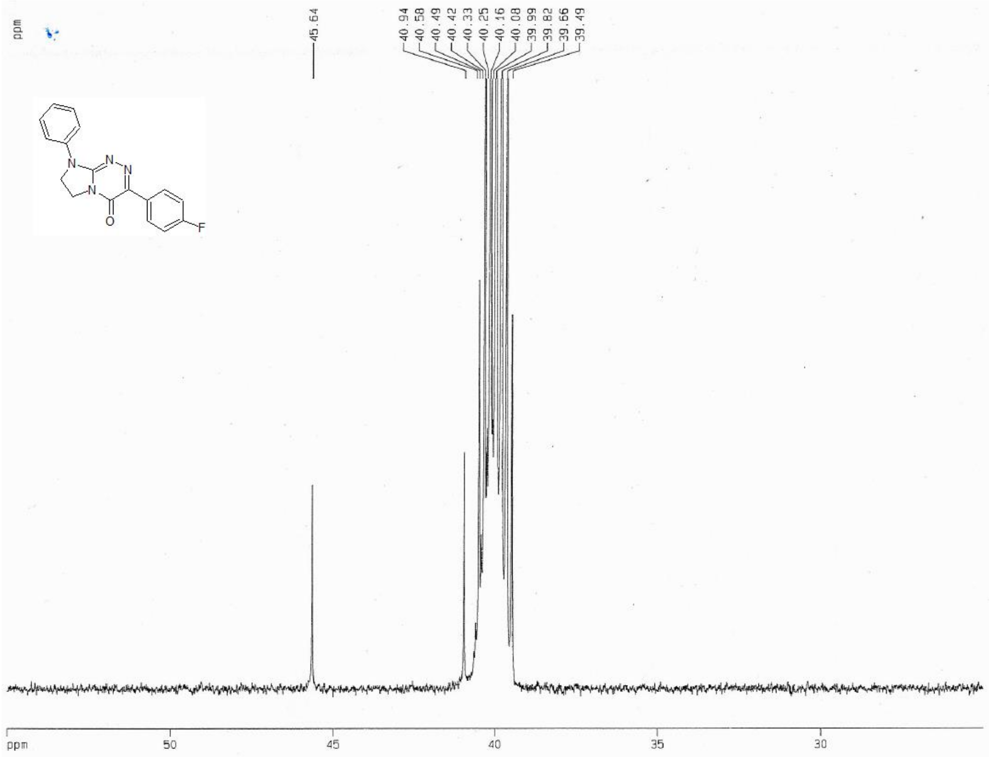

Figure S19. ^{13}C NMR spectrum in DMSO-d_6 for compound **10** (A), and expanded regions of the aromatic (B) and aliphatic (C) ranges with peak values.

A.

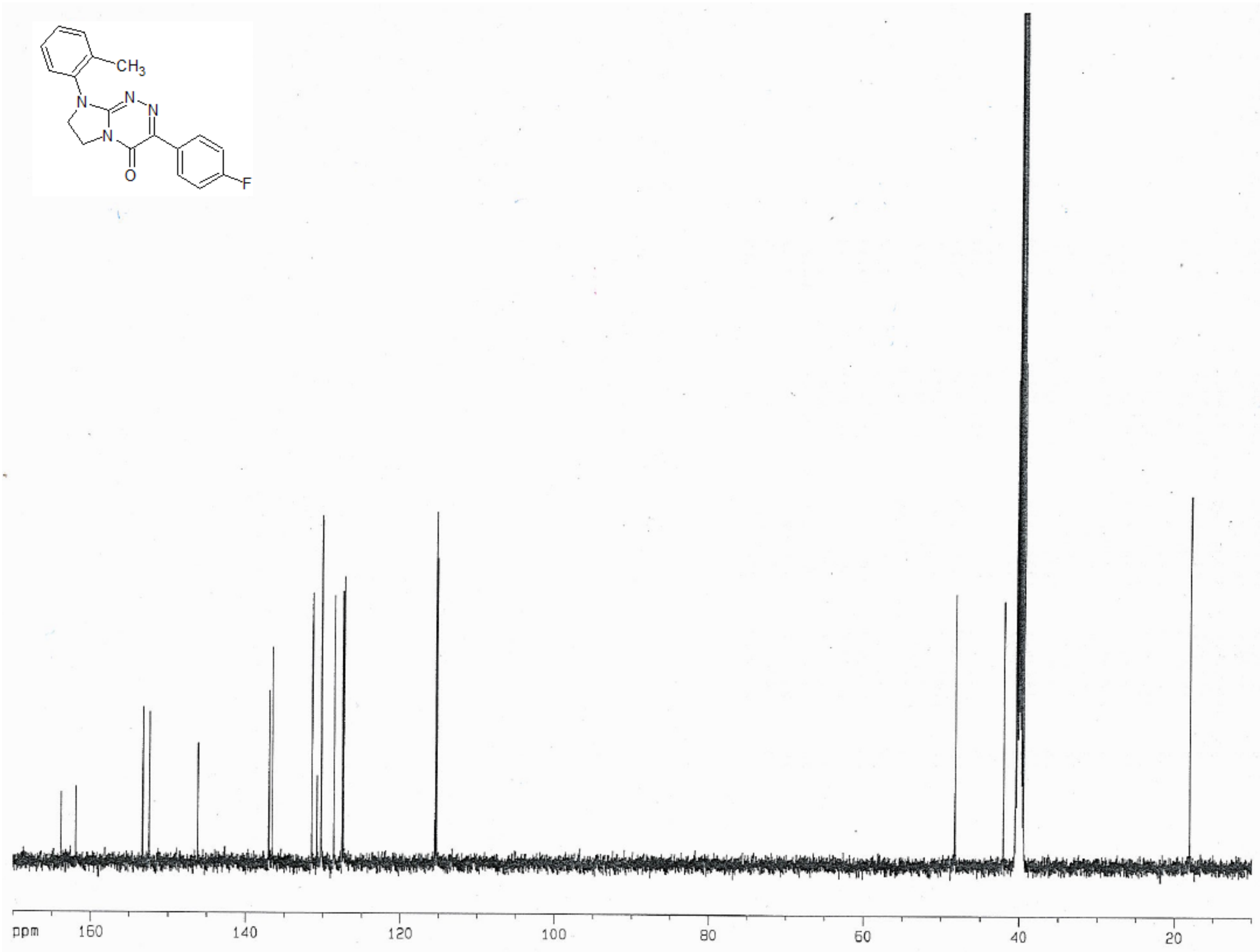

B.

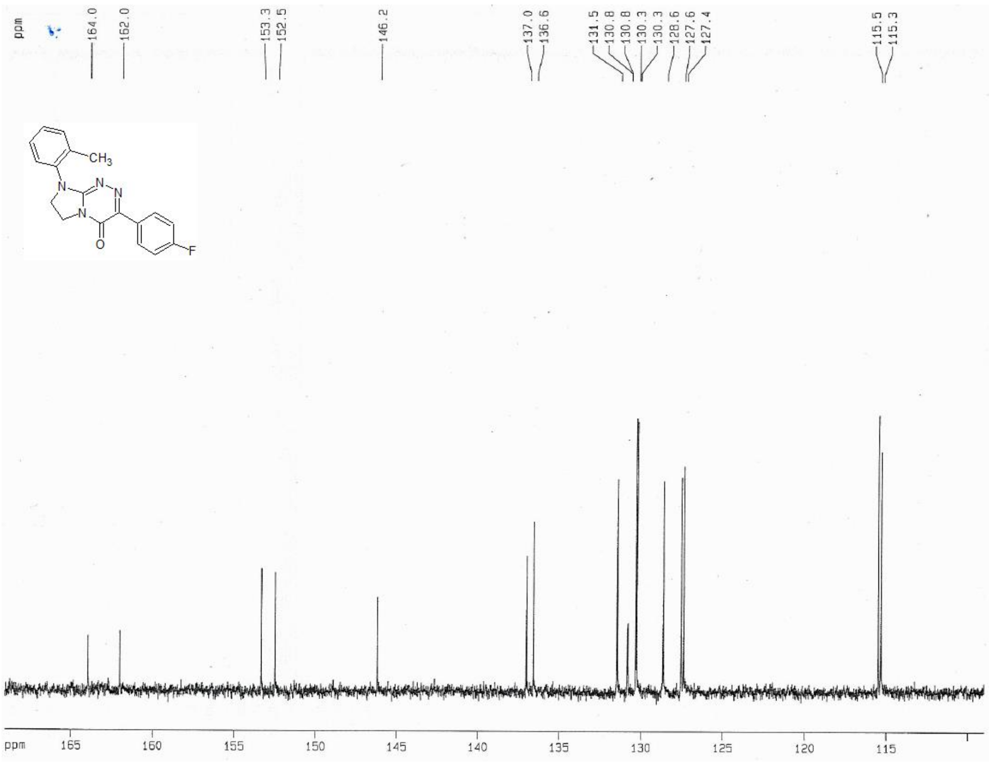

C.

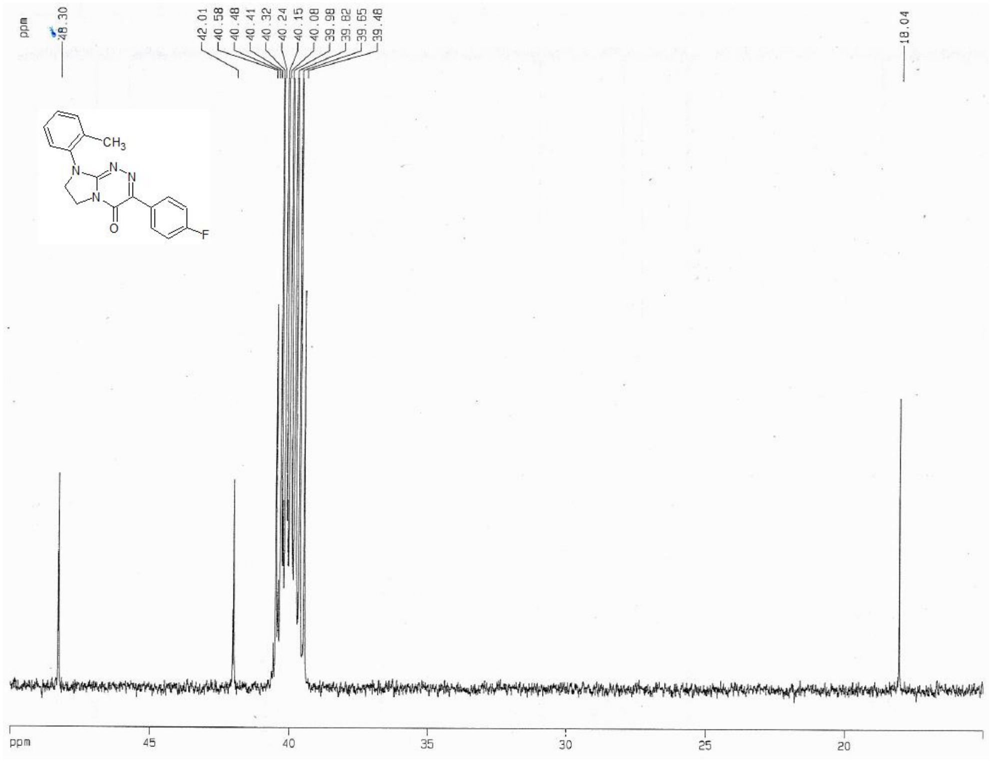

Figure S20. ^{13}C NMR spectrum in DMSO-d_6 for compound **11** (A), and expanded regions of the aromatic (B) and aliphatic (C) ranges with peak values.

A.

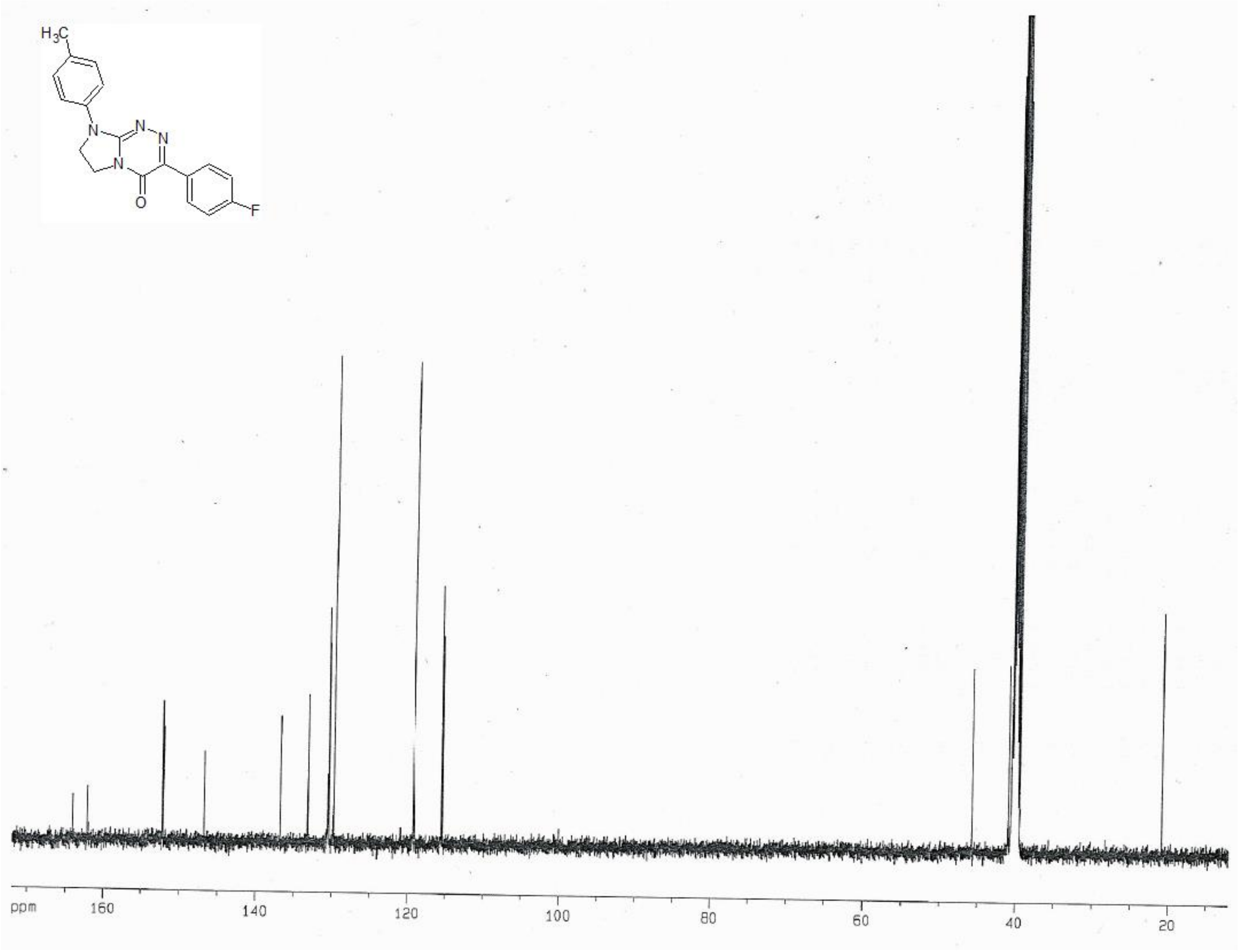

B.

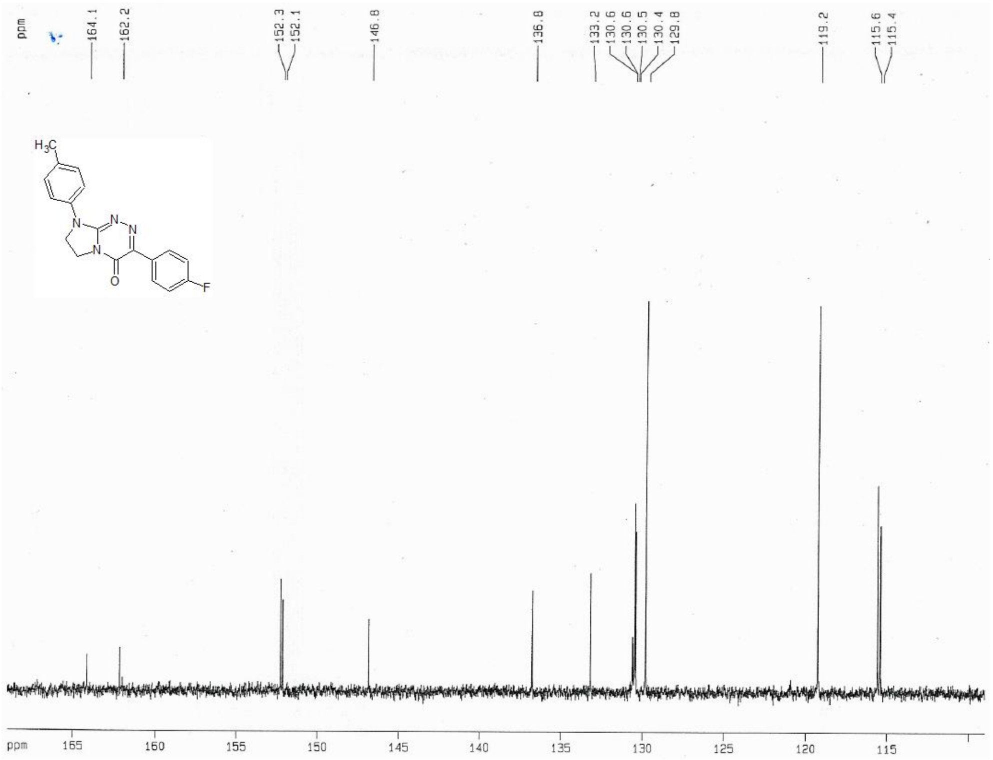

C.

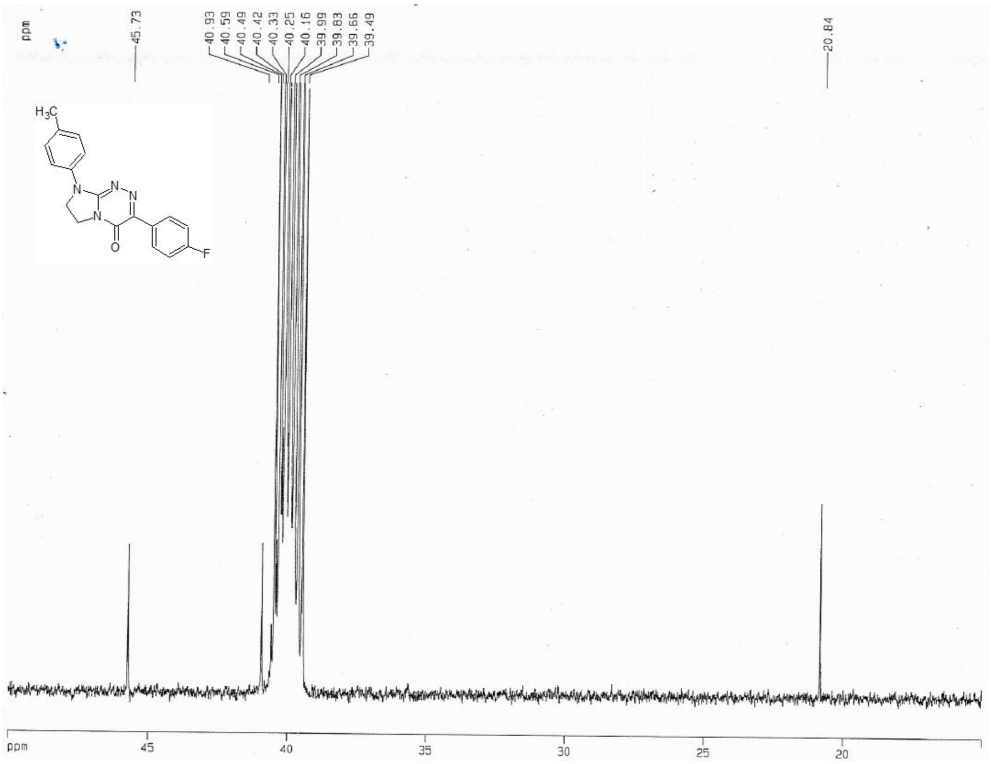

Figure S21. ^{13}C NMR spectrum in DMSO-d_6 for compound **12** (A), and expanded regions of the aromatic (B) and aliphatic (C) ranges with peak values.

A.

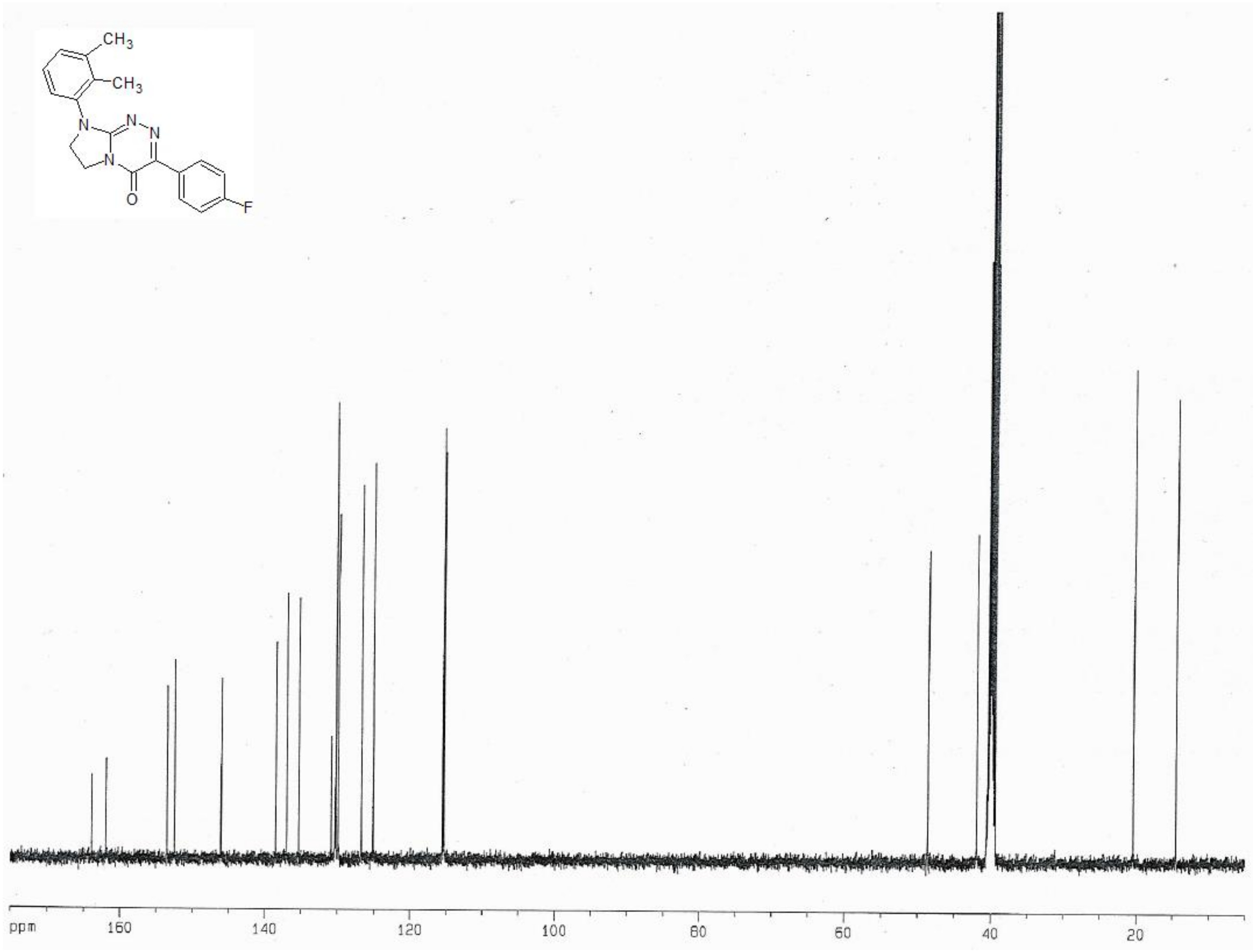

B.

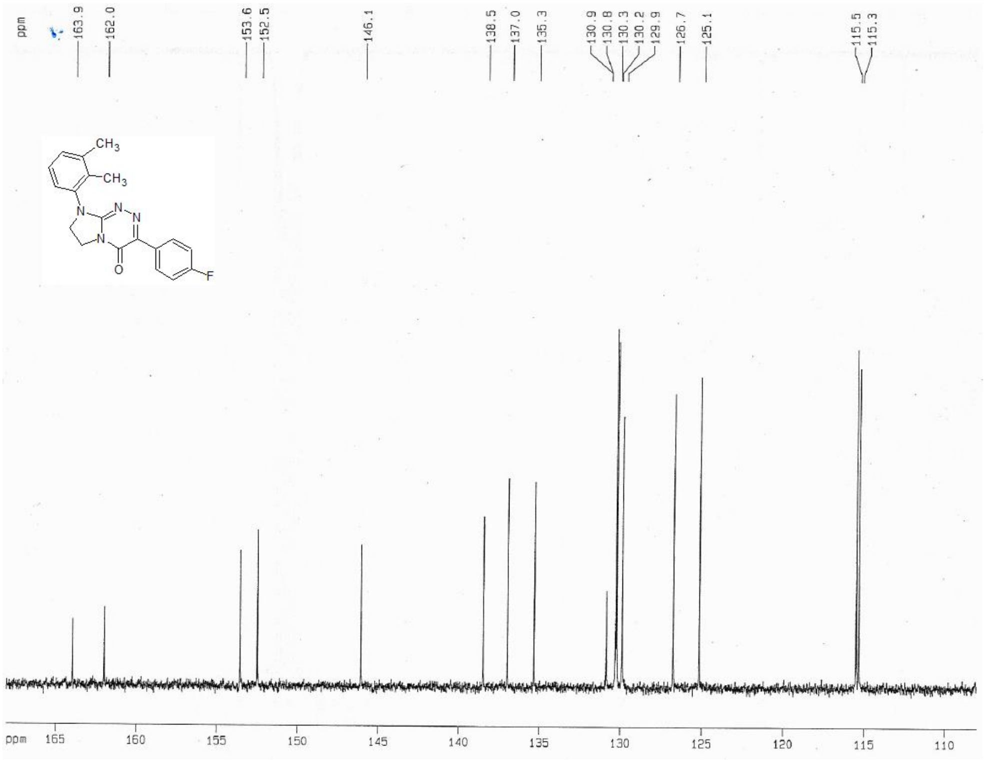

C.

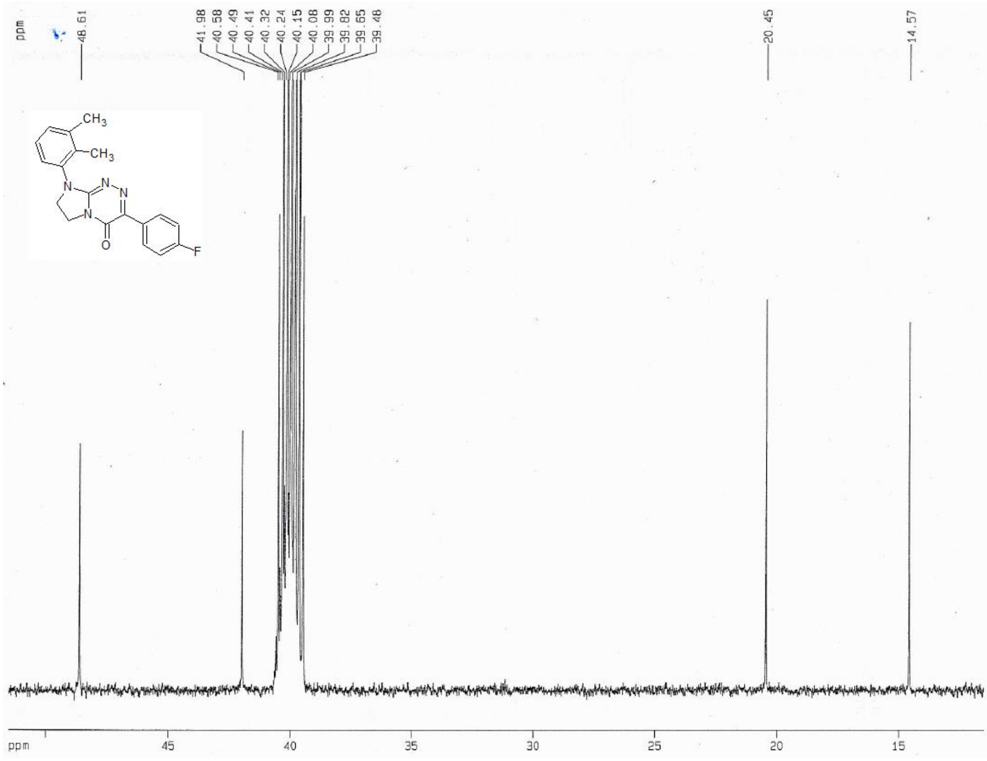

Figure S22. ^{13}C NMR spectrum in DMSO-d_6 for compound **13** (A), and expanded regions of the aromatic (B) and aliphatic (C) ranges with peak values.

A.

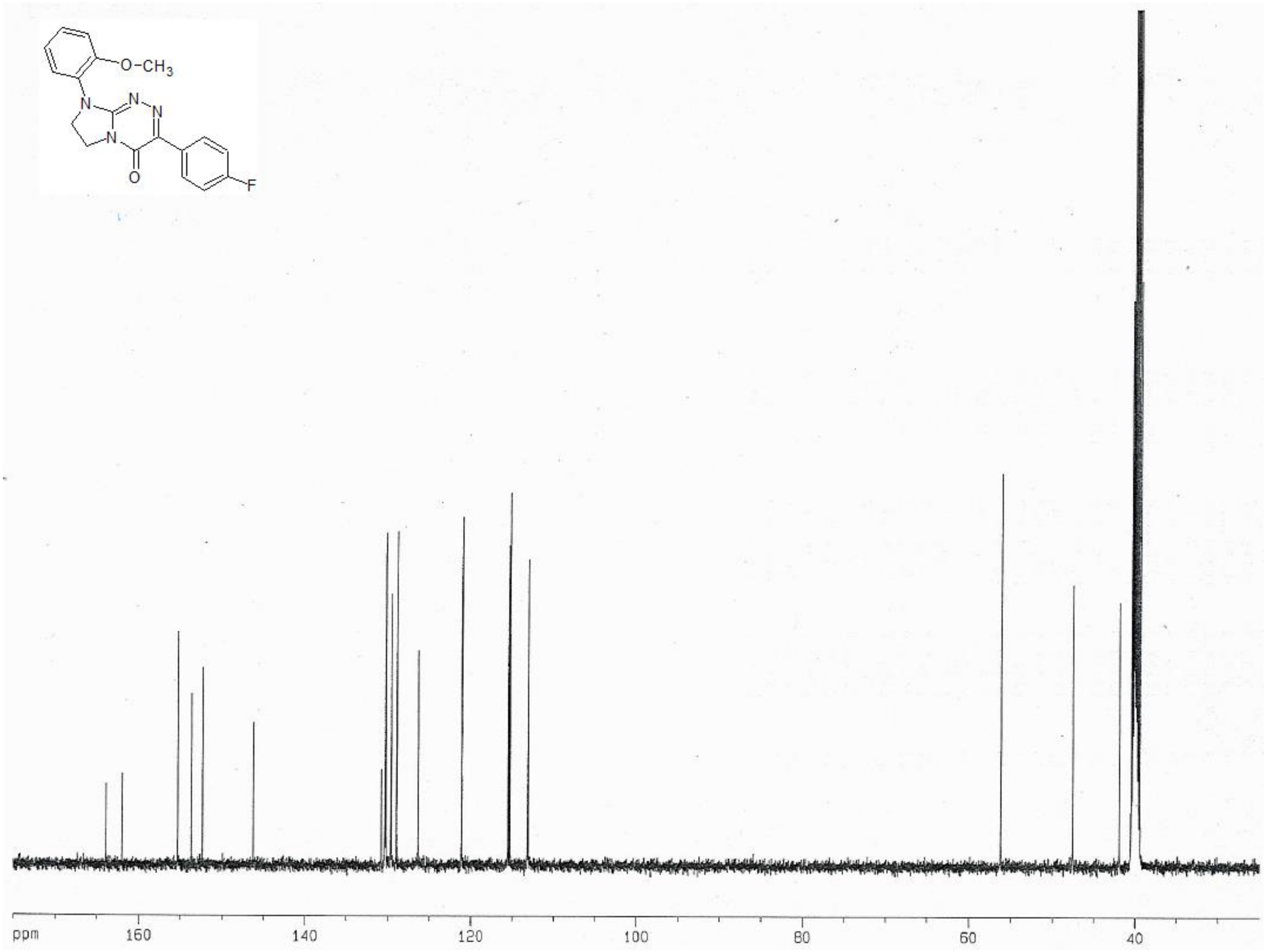

B.

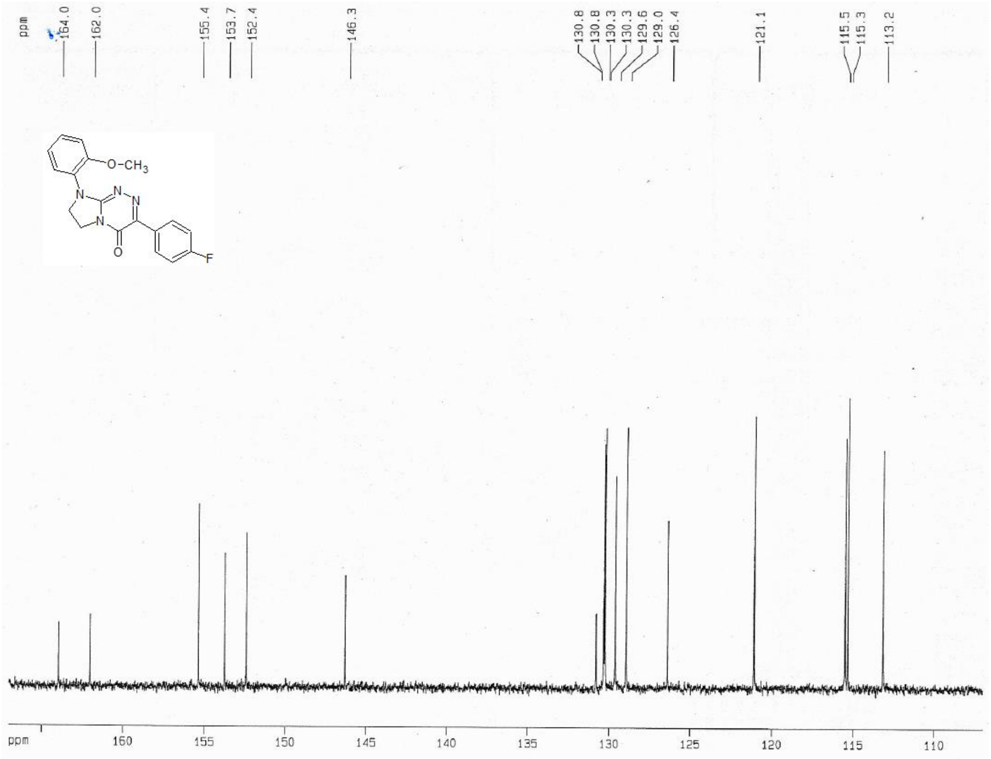

C.

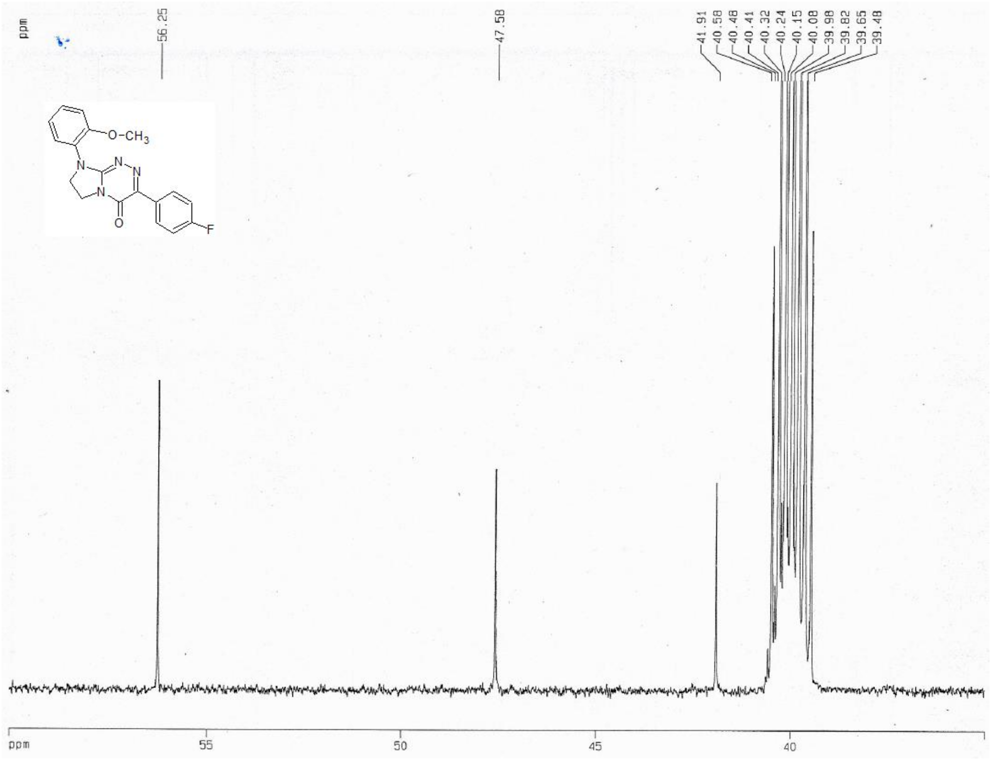

Figure S23. ^{13}C NMR spectrum in DMSO-d_6 for compound **14** (A), and expanded regions of the aromatic (B) and aliphatic (C) ranges with peak values.

A.

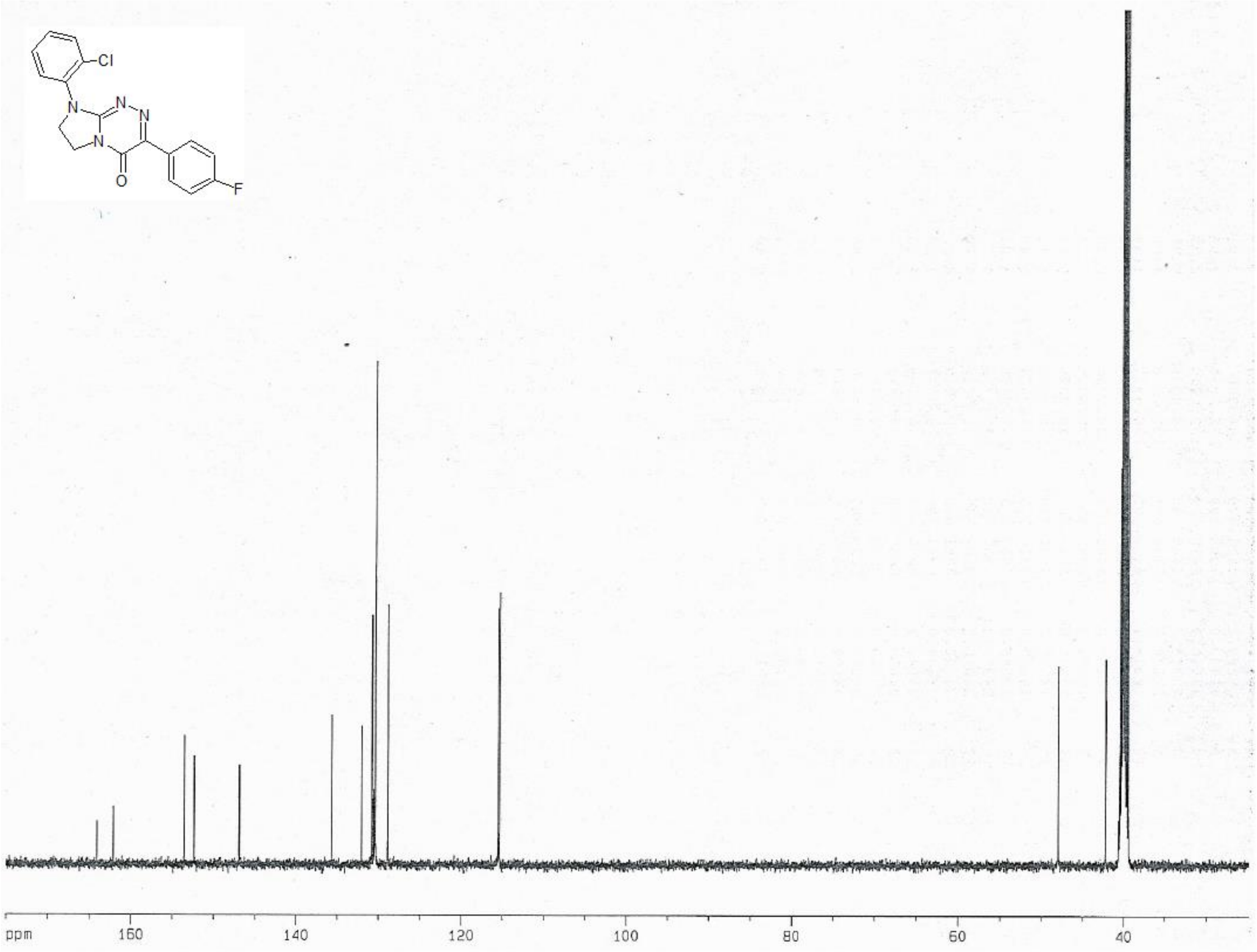

B.

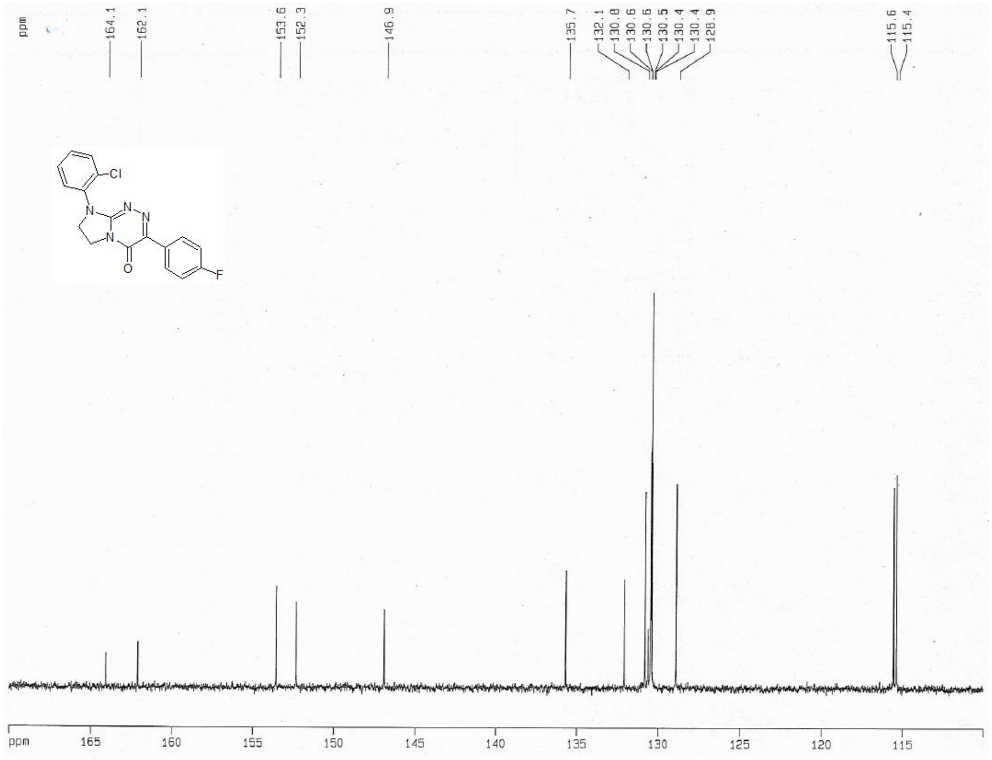

C.

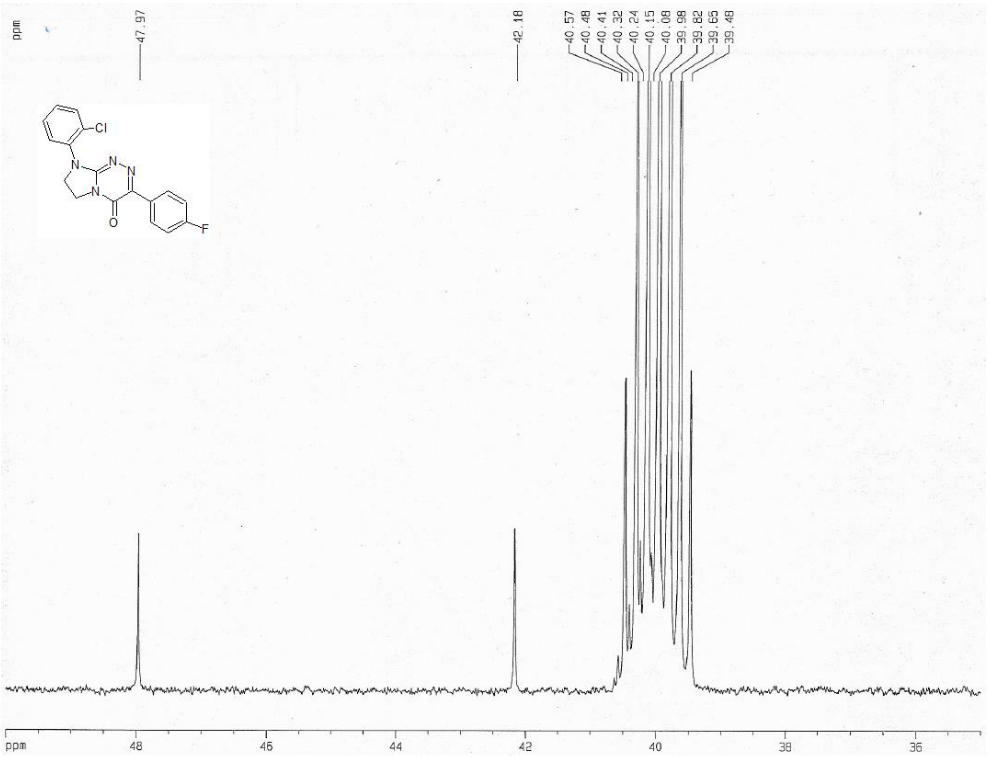

Figure S24. ^{13}C NMR spectrum in DMSO-d_6 for compound **15** (A), and expanded regions of the aromatic (B) and aliphatic (C) ranges with peak values.

A.

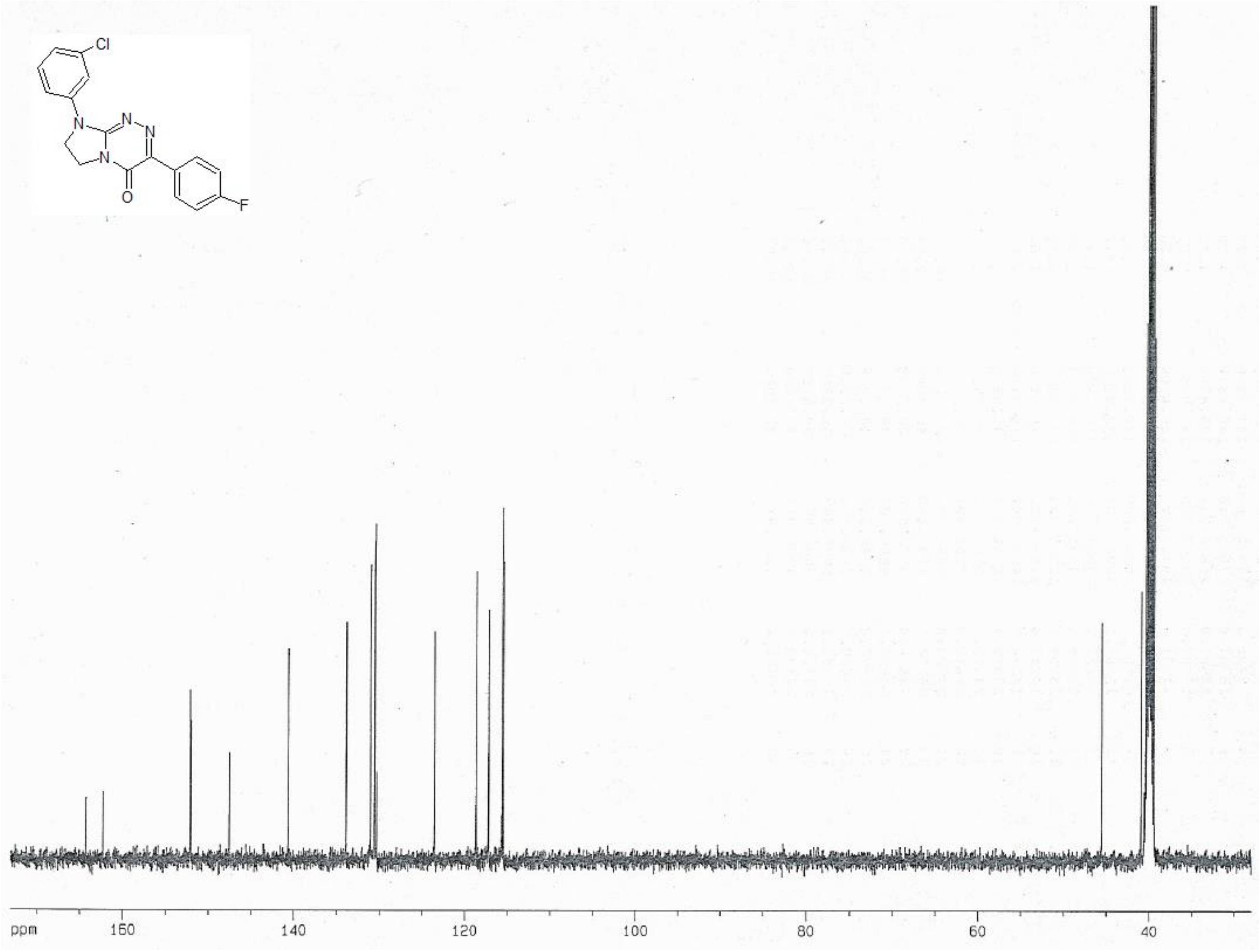

B.

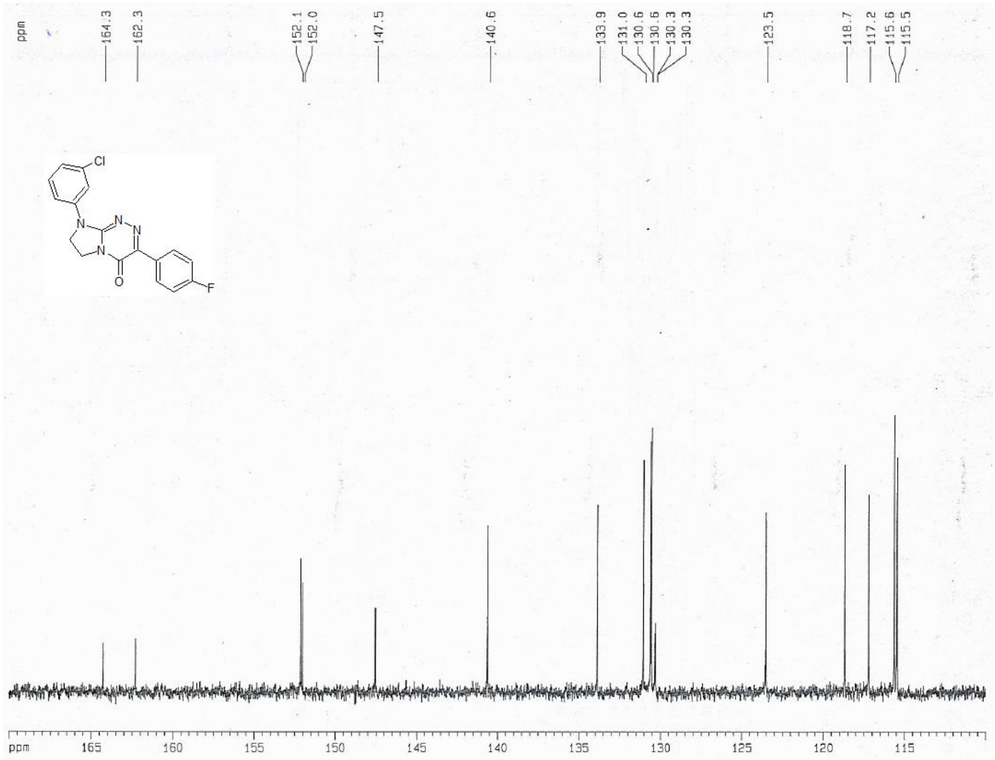

C.

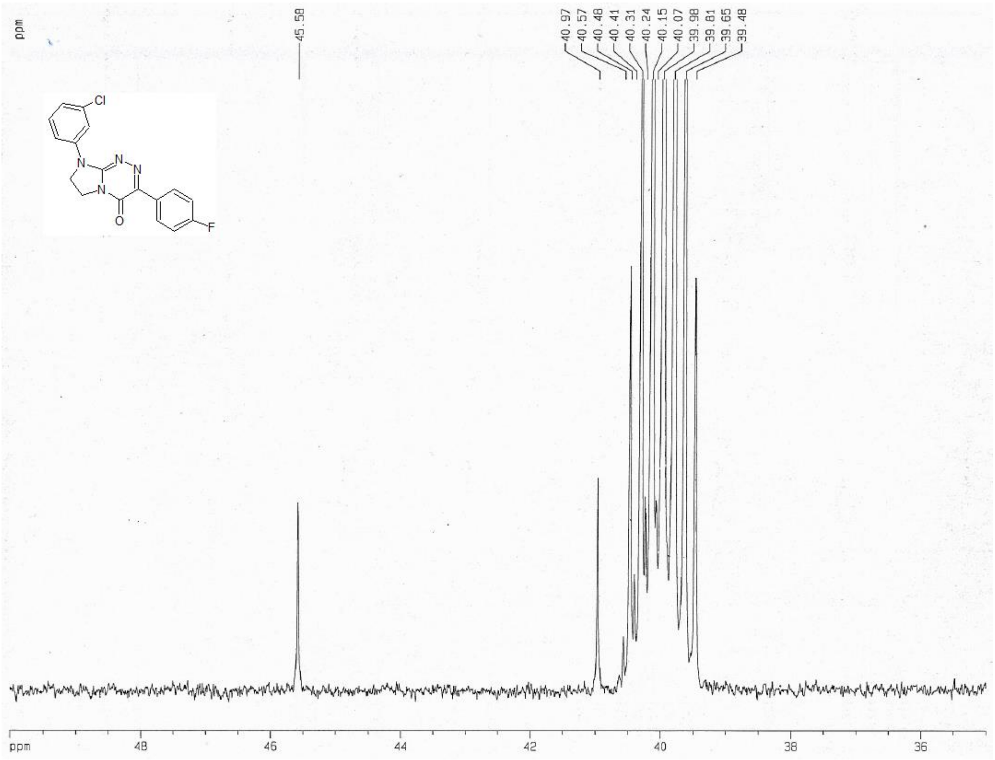

Figure S25. ^{13}C NMR spectrum in DMSO-d_6 for compound **16** (A), and expanded regions of the aromatic (B) and aliphatic (C) ranges with peak values.

A.

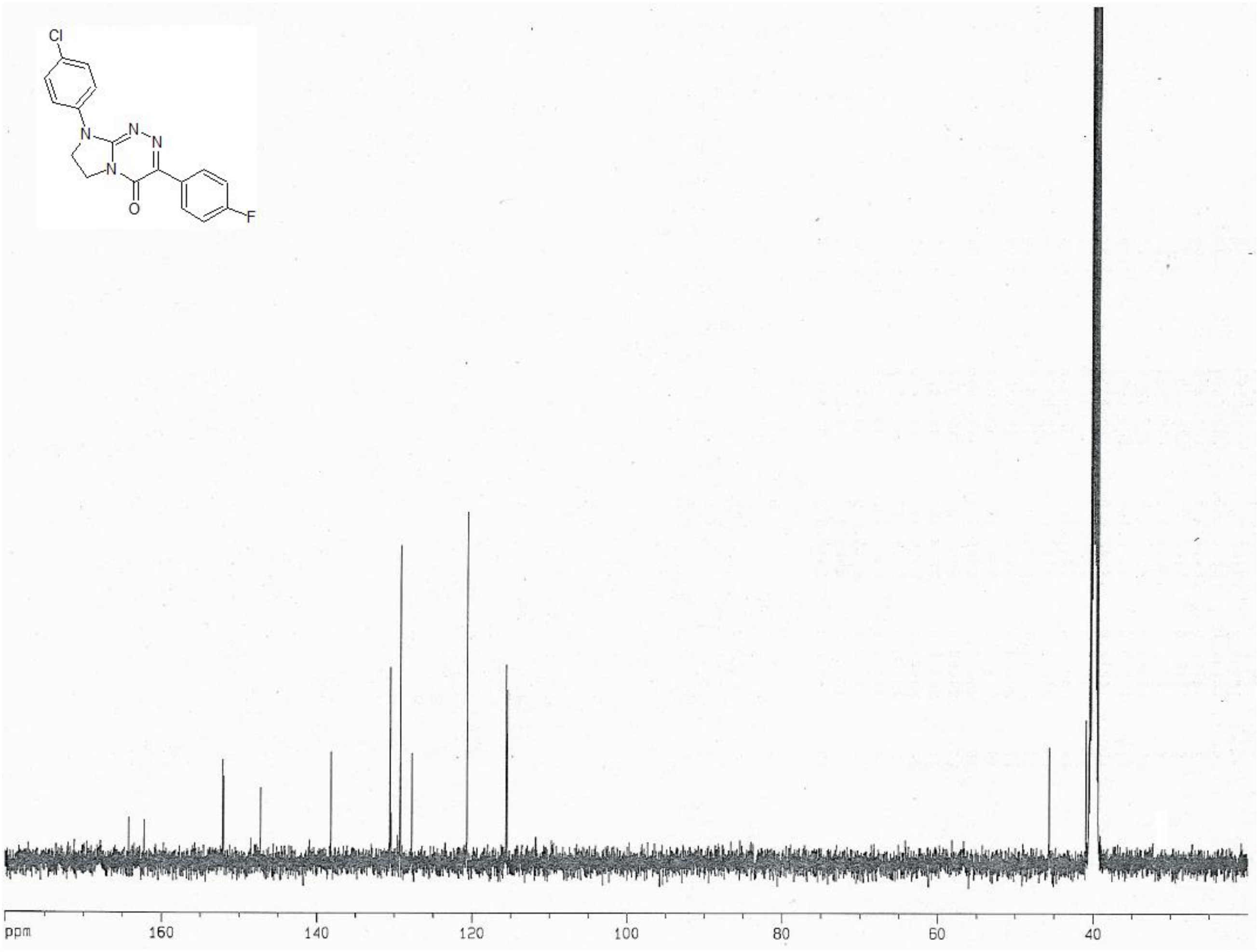

B.

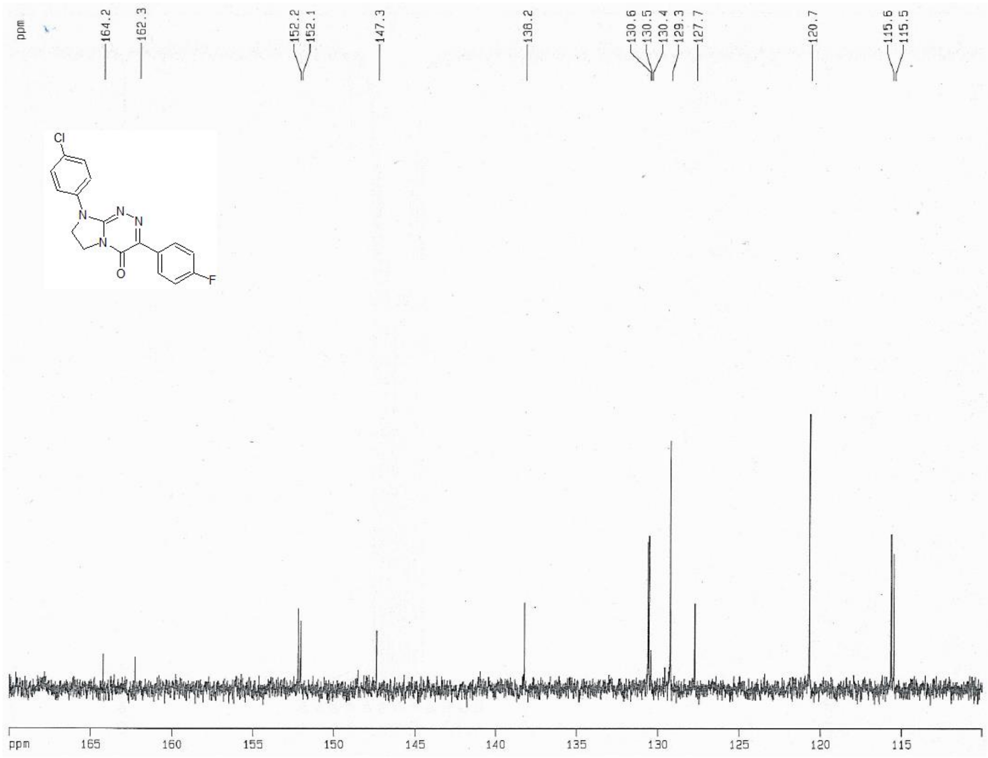

C.

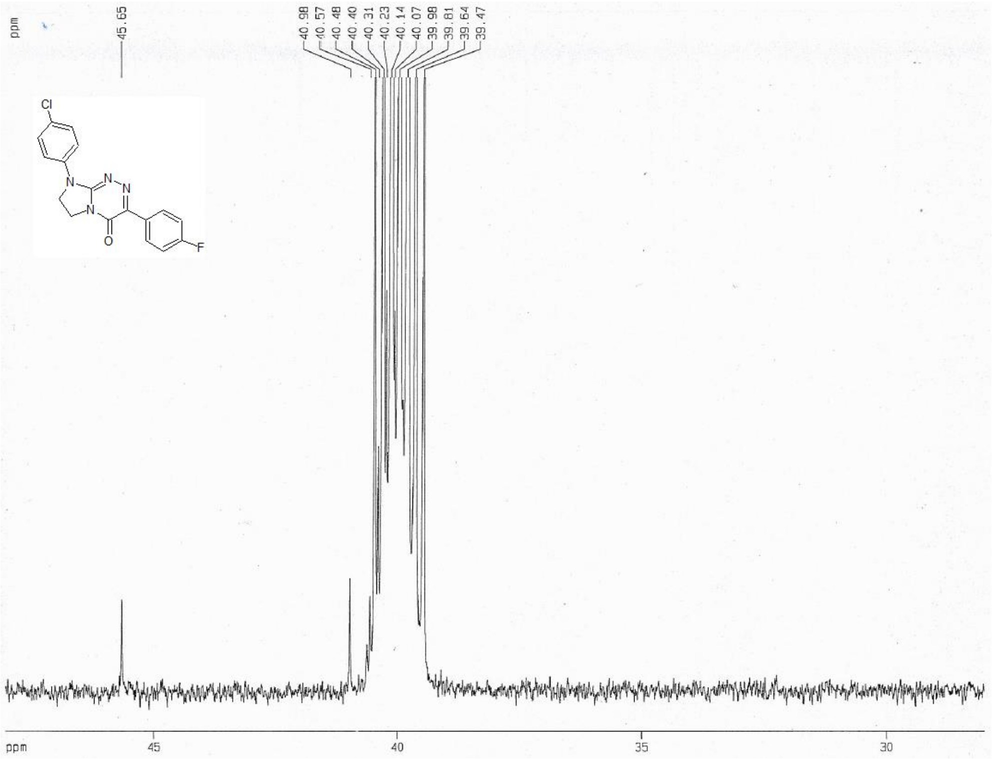

Figure S26. ^{13}C NMR spectrum in DMSO-d_6 for compound **17** (A), and expanded regions of the aromatic (B) and aliphatic (C) ranges with peak values.

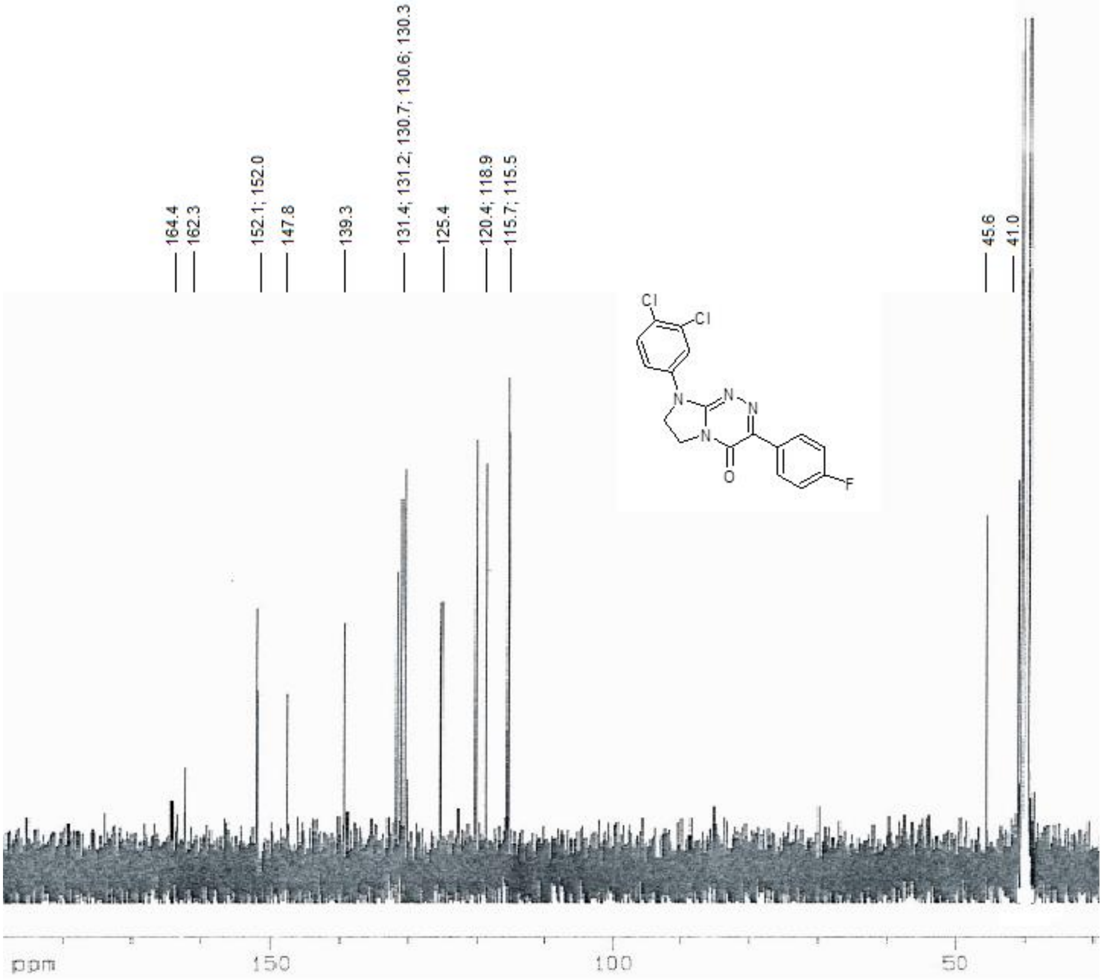

Figure S27. ^{13}C NMR spectrum in DMSO-d_6 for compound **18** with peak values.

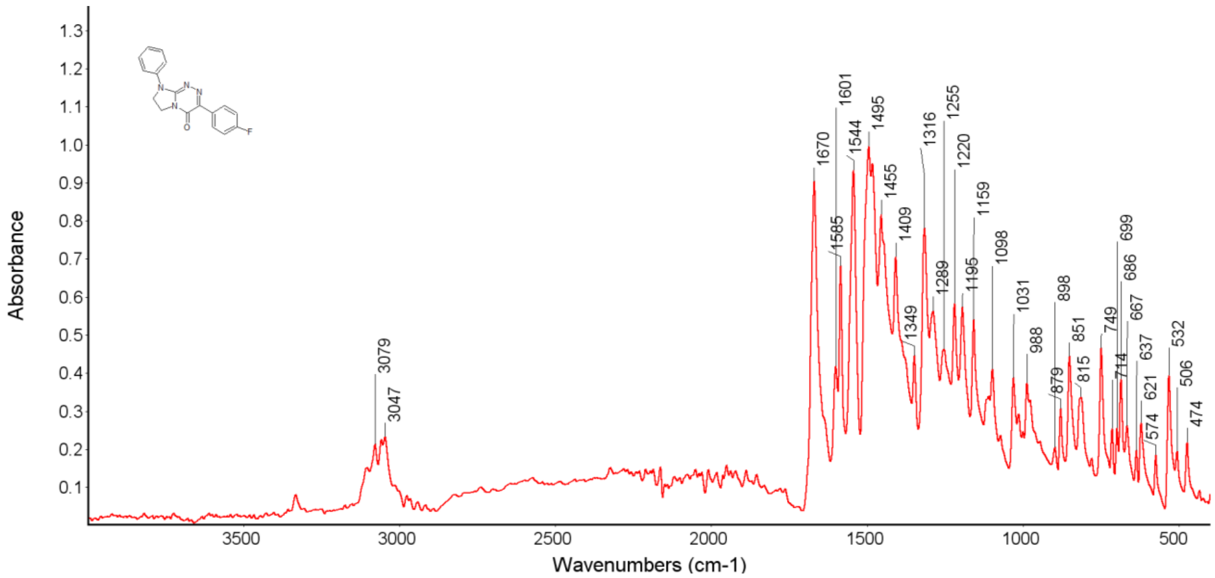

Figure S28. ATR-IR spectrum for compound **10.**

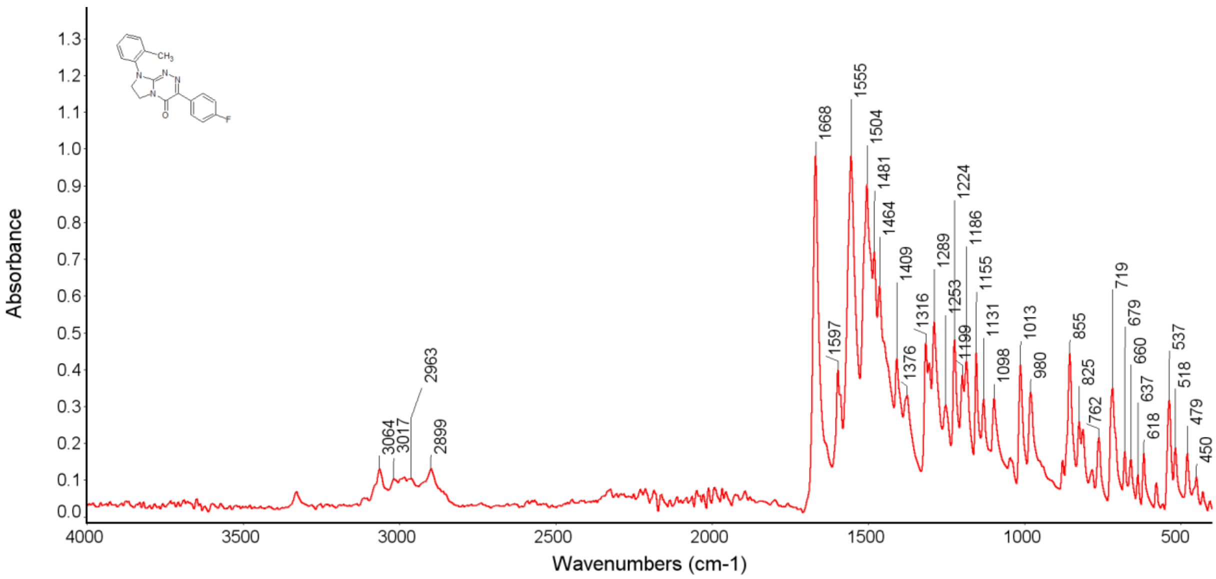

Figure S29. ATR-IR spectrum for compound **11.**

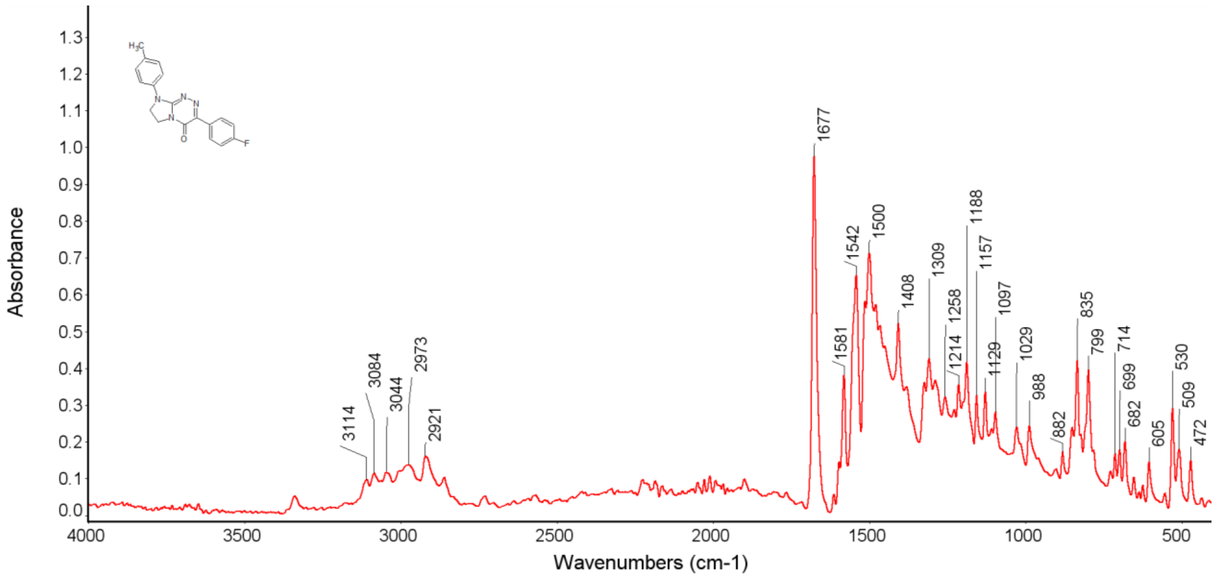

Figure S30. ATR-IR spectrum for compound **12.**

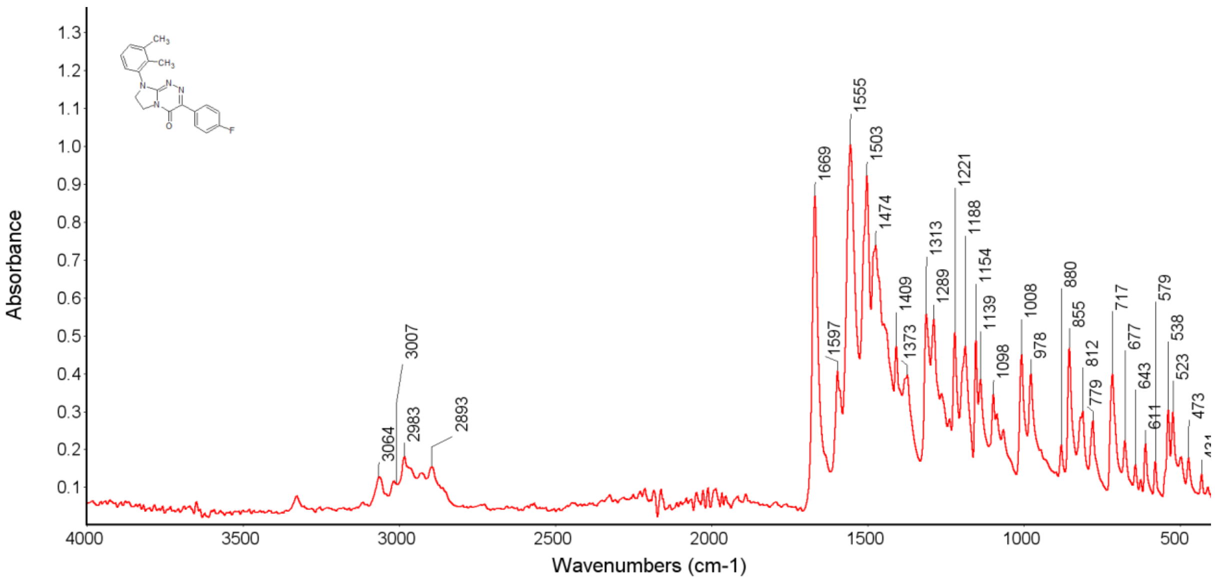

Figure S31. ATR-IR spectrum for compound **13.**

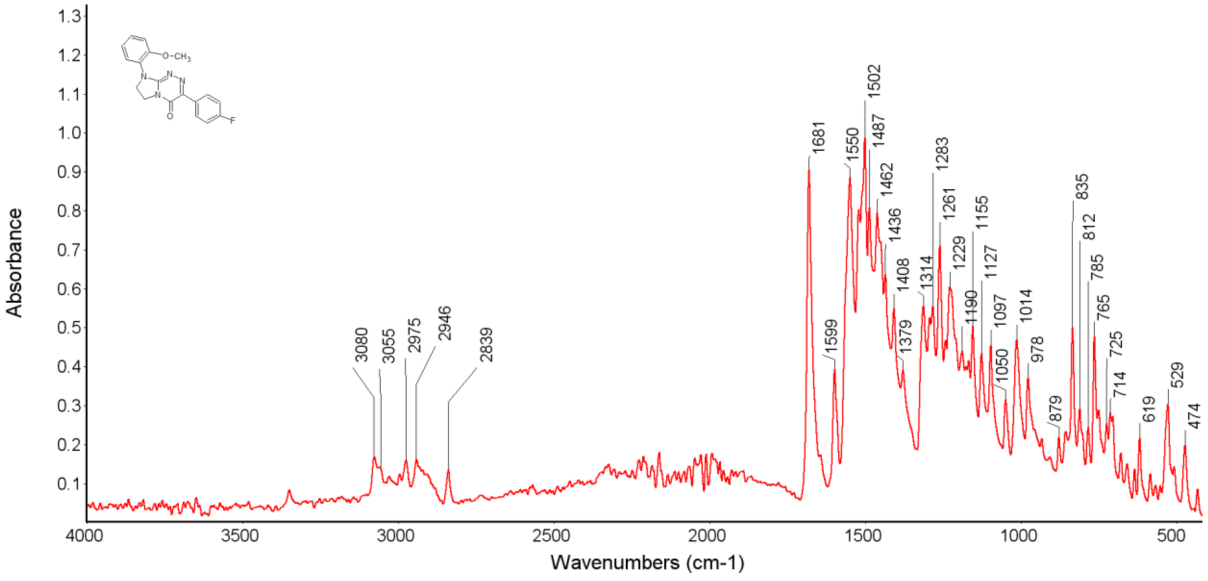

Figure S32. ATR-IR spectrum for compound **14.**

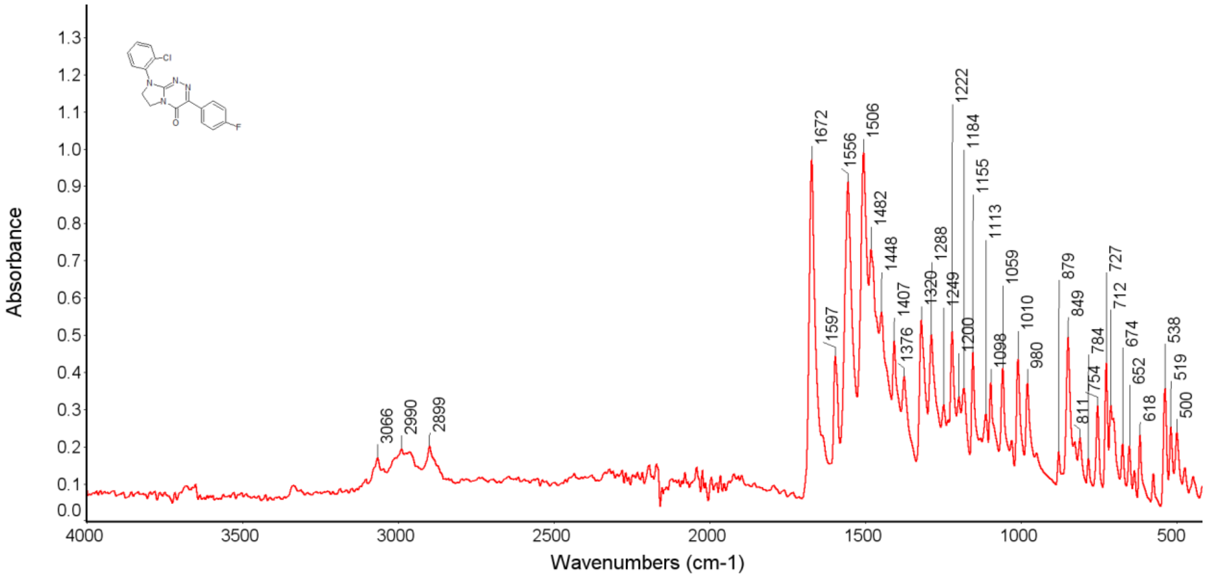

Figure S33. ATR-IR spectrum for compound **15.**

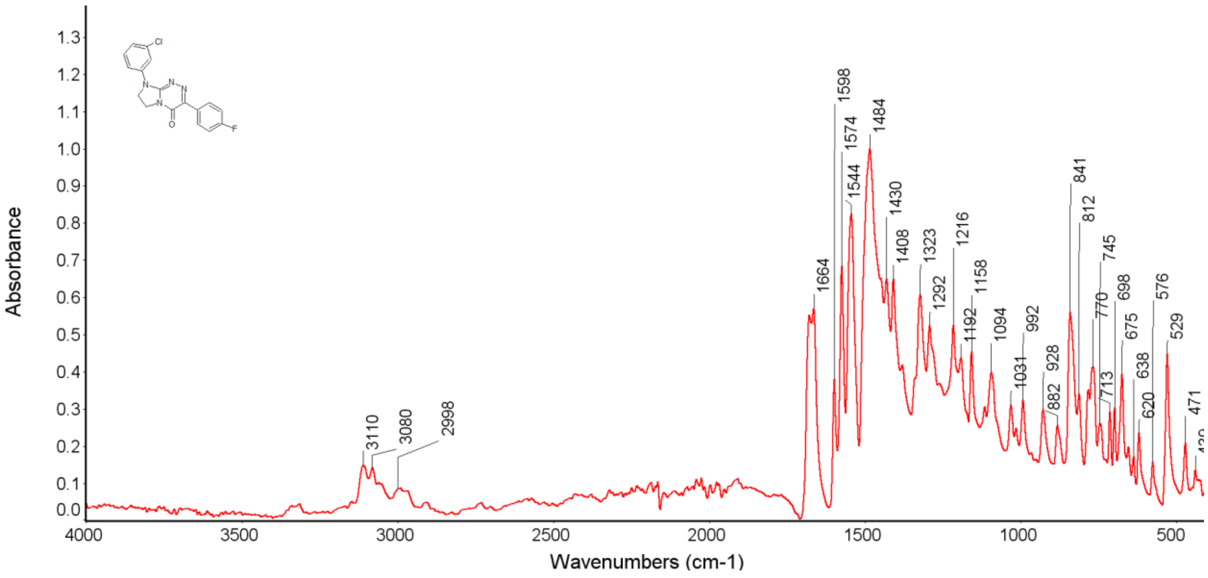

Figure S34. ATR-IR spectrum for compound **16.**

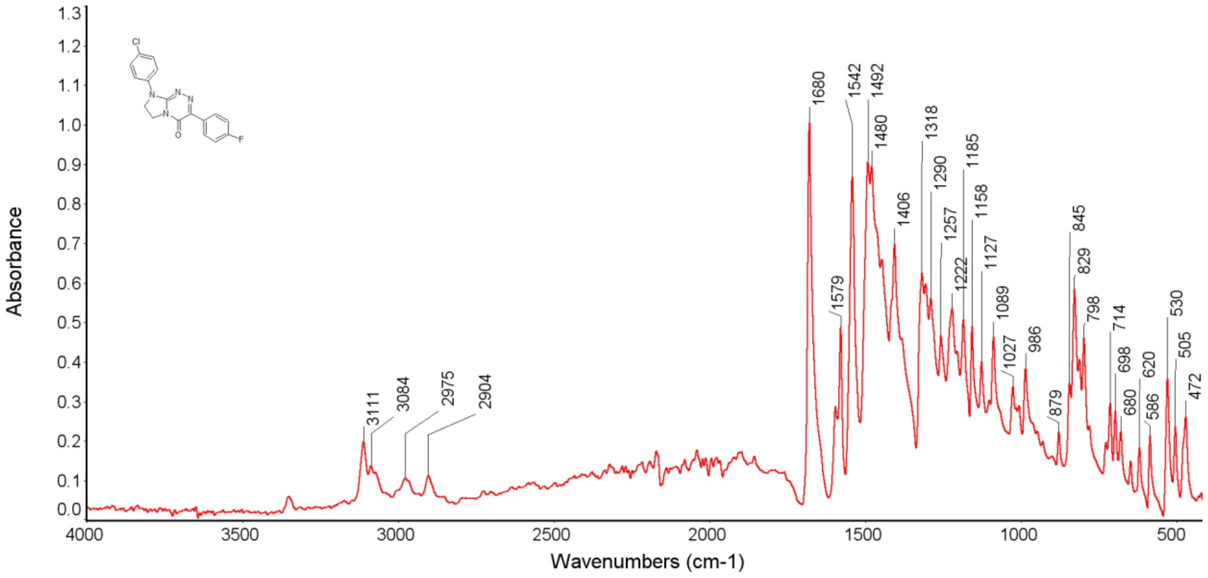

Figure S35. ATR-IR spectrum for compound **17.**

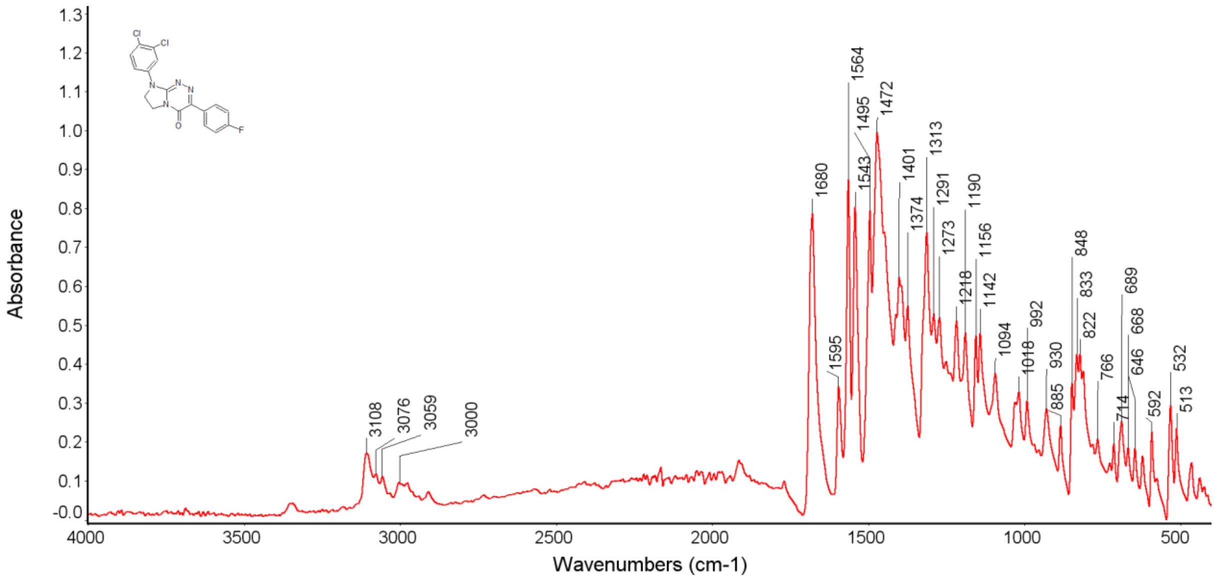

Figure S36. ATR-IR spectrum for compound **18.**

Table S1. Cytotoxicity of fluorophenylated fused 1,2,4-triazinones **10-18** in non-tumour and tumour cells of epithelial origin.

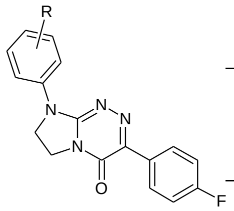

Compound	R	Incubation time	Cytotoxicity expressed as the percentage of growth inhibition			
			GMK	A549	HeLa	T47D
**10**	H	24 h	50 ± 5.4*	10 ± 1.6	95 ± 7.5**	75 ± 5.4**
		48 h	55 ± 4.8*	45 ± 3.8*	98 ± 8.8**	85 ± 7.8**
		72 h	90 ± 9.8**	55 ± 4.6*	100 ± 10.2**	100 ± 9.2**
**11**	2-CH_3	24 h	10 ± 1.6	10 ± 0.9	60 ± 4.4*	5 ± 0.2
		48 h	20 ± 2.2	20 ± 1.8	75 ± 5.5**	45 ± 3.6*
		72 h	35 ± 3.8*	25 ± 2.7	95 ± 4.9**	85 ± 7.5**
**12**	4-CH_3	24 h	90 ± 7.6**	85 ± 9.2**	85 ± 7.3**	70 ± 6.5**
		48 h	95 ± 9.2**	90 ± 8.2**	90 ± 8.9**	85 ± 8.1**
		72 h	98 ± 10.2**	100 ± 9.5**	100 ± 9.5**	100 ± 10.2**
**13**	2,3-$(CH_3)_2$	24 h	15 ± 0.9	15 ± 1.2	30 ± 2.8*	5 ± 0.4
		48 h	30 ± 2.8*	45 ± 3.6*	50 ± 4.7*	15 ± 0.9
		72 h	40 ± 3.5*	90 ± 8.8**	85 ± 5.9**	50 ± 4.6*
**14**	2-OCH_3	24 h	20 ± 0.8	25 ± 1.4	60 ± 3.8*	15 ± 0.9
		48 h	40 ± 2.2*	55 ± 4.7*	65 ± 5.2**	35 ± 3.2*
		72 h	45 ± 3.6*	80 ± 8.2**	80 ± 7.2**	45 ± 4.4*
**15**	2-Cl	24 h	2 ± 0.1	45 ± 2.8*	45 ± 3.7*	5 ± 0.3
		48 h	15 ± 0.9	55 ± 4.6*	75 ± 5.8**	30 ± 2.8*
		72 h	20 ± 1.8*	65 ± 5.5**	90 ± 8.2**	45 ± 4.2*
**16**	3-Cl	24 h	20 ± 2.5	60 ± 4.7**	75 ± 7.1**	45 ± 3.8*
		48 h	50 ± 2.5*	75 ± 6.2**	90 ± 8.3**	55 ± 4.7*
		72 h	90 ± 10.2**	85 ± 7.2**	100 ± 8.8**	75 ± 6.4**
**17**	4-Cl	24 h	5 ± 0.6	15 ± 0.9	80 ± 7.6**	35 ± 2.8*
		48 h	10 ± 0.8	55 ± 3.2*	95 ± 9.1**	75 ± 7.2**
		72 h	25 ± 2.5*	85 ± 7.9**	100 ± 9.9**	100 ± 7.0**
**18**	3,4-Cl_2	24 h	65 ± 5.8**	85 ± 8.2**	90 ± 6.3**	30 ± 2.2*
		48 h	80 ± 7.5**	98 ± 8.8**	98 ± 8.1**	85 ± 7.8**
		72 h	90 ± 10.2**	100 ± 9.5**	100 ± 9.4**	100 ± 9.6**
Pemetrexed		24 h	4 ± 0.2	13 ± 0.8	13 ± 1.0	4 ± 0.3
		48 h	18 ± 1.1*	25 ± 1.5*	42 ± 2.1*	13 ± 0.6
		72 h	21 ± 1.2*	42 ± 2.7*	50 ± 3.8**	21 ± 0.8*
Gemcitabine		24 h	0	25 ± 0.7	25 ± 0.8*	2 ± 0.1
		48 h	45 ± 1.3*	80 ± 1.6**	80 ± 1.2**	5 ± 0.3
		72 h	55 ± 0.9	95 ± 1.8**	100 ± 1.9**	55 ± 0.8**

Non-tumour cell line (from the European Collection of Authenticated Cell Cultures): GMK (ECACC 88020401) – African green monkey kidney cells. Cancer cell lines (from the European Collection of Authenticated Cell Cultures): A549 (ECACC 86012804) – human Caucasian lung carcinoma cells; HeLa (ECACC 93021013) – human Negroid cervix epithelioid carcinoma cells; T47D (ECACC 85102201) – human breast carcinoma cells. All compounds, pemetrexed and gemcitabine were tested at a concentration of 150 μM. Data (from five independent experiments) are provided as the mean ± standard deviation. *, ** – statistically different from the control (* $p < 0.05$; ** $p < 0.001$, Student's *t*-test).

Table S2. Cytotoxicity of fluorophenylated fused 1,2,4-triazinones **10-18** in MM.1R, Jurkat E6-1 and HL-60 cells.

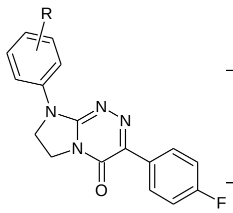

Compound	R	Incubation time	Cytotoxicity expressed as the percentage of dead tumour cells		
			**MM.1R**	**Jurkat E6-1**	**HL-60**
**10**	H	24 h	50 ± 2.8*	45 ± 3.2*	25 ± 1.3*
		48 h	60 ± 2.4**	60 ± 1.5**	40 ± 1.5*
		72 h	85 ± 5.6**	75 ± 4.2**	50 ± 0.6*
**11**	2-CH_3	24 h	55 ± 4.6*	30 ± 2.4*	50 ± 4.5*
		48 h	65 ± 2.2**	40 ± 2.2*	55 ± 2.2*
		72 h	85 ± 4.8**	50 ± 2.2*	60 ± 2.2**
**12**	4-CH_3	24 h	50 ± 3.5*	40 ± 3.8	60 ± 2.8*
		48 h	60 ± 3.6*	50 ± 4.5*	75 ± 4.6**
		72 h	80 ± 5.2**	60 ± 4.2*	85 ± 4.4**
**13**	2,3-$(CH_3)_2$	24 h	65 ± 2.6**	25 ± 2.2*	75 ± 4.2**
		48 h	70 ± 4.2**	40 ± 2.4*	100 ± 4.5**
		72 h	75 ± 4.6**	50 ± 1.8*	100 ± 2.8**
**14**	2-OCH_3	24 h	60 ± 5.4*	20 ± 1.8	75 ± 3.6**
		48 h	80 ± 2.8**	25 ± 0.9*	80 ± 4.4**
		72 h	90 ± 2.5**	40 ± 2.2*	100 ± 3.6**
**15**	2-Cl	24 h	45 ± 3.1*	10 ± 0.7	80 ± 2.8**
		48 h	60 ± 4.5*	15 ± 0.8	100 ± 3.8**
		72 h	70 ± 1.8**	35 ± 1.6*	100 ± 4.2**
**16**	3-Cl	24 h	10 ± 0.6	10 ± 0.5	25 ± 1.2*
		48 h	15 ± 0.9	20 ± 1.1	40 ± 1.6*
		72 h	25 ± 0.8	45 ± 2.5*	70 ± 2.5**
**17**	4-Cl	24 h	55 ± 4.2*	50 ± 3.6*	50 ± 2.6*
		48 h	70 ± 3.6**	60 ± 4.6*	60 ± 3.2**
		72 h	90 ± 3.6**	80 ± 3.5**	80 ± 3.6**
**18**	3,4-Cl_2	24 h	15 ± 1.1	20 ± 1.2	75 ± 3.2**
		48 h	20 ± 0.9*	25 ± 1.2*	90 ± 3.8**
		72 h	30 ± 1.1*	50 ± 2.2*	100 ± 4.8**
Gemcitabine		24 h	-	-	15 ± 0.5
		48 h	-	-	25 ± 1.4*
		72 h	-	-	35 ± 0.9*

Tumour cell line derived from human peripheral blood lymphoblast B cells (from the American Type Culture Collection): MM.1R (ATCC CRL2975) – human multiple myeloma cells. Leukaemic cell lines (from the European Collection of Authenticated Cell Cultures): Jurkat E6-1 (ECACC 88042803) – human acute T-lymphocytic leukaemia cells; HL-60 (ECACC 98070106) – human acute promyelocytic leukaemia cells. All compounds and gemcitabine were tested at a concentration of 150 μM. Data (from two independent experiments) are shown as the mean ± standard deviation. *, ** – statistically different from the control (* $p < 0.05$; ** $p < 0.001$, Student's *t*-test).
